# Calreticulin exposure correlates with robust adaptive antitumor immunity and favorable prognosis in ovarian carcinoma patients

**DOI:** 10.1186/s40425-019-0781-z

**Published:** 2019-11-20

**Authors:** Lenka Kasikova, Michal Hensler, Iva Truxova, Petr Skapa, Jan Laco, Lucie Belicova, Ivan Praznovec, Sarka Vosahlikova, Michael J. Halaska, Tomas Brtnicky, Lukas Rob, Jiri Presl, Jan Kostun, Isabelle Cremer, Ales Ryska, Guido Kroemer, Lorenzo Galluzzi, Radek Spisek, Jitka Fucikova

**Affiliations:** 1 Department of Immunology, Charles University, 2nd Faculty of Medicine and University Hospital Motol, V Uvalu 84, 150 00 Prague 5, Czech Republic; 2 Sotio, Prague, Czech Republic; 3 Department of Pathology and Molecular Medicine, Charles University, 2nd Faculty of Medicine and University Hospital Motol, Prague, Czech Republic; 4 The Fingerland Department of Pathology, Charles University, Faculty of Medicine and University Hospital, Hradec Kralove, Czech Republic; 5 Department of Gynecology and Obstetrics, Charles University, Faculty of Medicine and University Hospital, Hradec Kralove, Czech Republic; 6 Department of Gynecology and Obstetrics, Charles University, 3rd Faculty of Medicine and University Hospital Kralovske Vinohrady, Prague, Czech Republic; 7 Department of Gynecology and Obstetrics, Charles University, 2nd Faculty of Medicine and University Hospital Motol, Prague, Czech Republic; 8 Department of Gynecology and Obstetrics, Faculty of Medicine and University Hospital Plzen, Pilsen, Czech Republic; 9 Inflammation, Complement and Cancer, INSERM, U1138, Centre de Recherche des Cordeliers, Paris, France; 10 Sorbonne Université, Paris, France; 11 Université Paris Descartes, Paris, France, Paris, France; 12 Metabolomics and Cell Biology Platforms, Institut Gustave Roussy, Villejuif, France; 13 Pôle de Biologie, Hôpital Européen Georges Pompidou, AP-HP, Paris, France; 14 Suzhou Institute for Systems Biology, Chinese Academy of Sciences, Suzhou, China; 15 Karolinska Institute, Department of Women’s and Children’s Health, Karolinska University Hospital, Stockholm, Sweden; 16 Department of Radiation Oncology, Weill Cornell Medical College, New York, NY USA; 17 Sandra and Edward Meyer Cancer Center, New York, NY USA; 18 Department of Dermatology, Yale School of Medicine, New Haven, CT USA

**Keywords:** B cells, Cancer immunotherapy, CD20, DC-LAMP, Dendritic cells, Immunogenic cell death

## Abstract

**Background:**

Adjuvanticity, which is the ability of neoplastic cells to deliver danger signals, is critical for the host immune system to mount spontaneous and therapy-driven anticancer immune responses. One of such signals*,* i.e., the exposure of calreticulin (CALR) on the membrane of malignant cells experiencing endoplasmic reticulum (ER) stress, is well known for its role in the activation of immune responses to dying cancer cells. However, the potential impact of CALR on the immune contexture of primary and metastatic high-grade serous carcinomas (HGSCs) and its prognostic value for patients with HGSC remains unclear.

**Method:**

We harnessed a retrospective cohort of primary (no = 152) and metastatic (no = 74) tumor samples from HGSC patients to investigate the CALR expression in relation with prognosis and function orientation of the tumor microenvironment. IHC data were complemented with transcriptomic and functional studies on second prospective cohort of freshly resected HGSC samples. In silico analysis of publicly available RNA expression data from 302 HGSC samples was used as a confirmatory approach.

**Results:**

We demonstrate that CALR exposure on the surface of primary and metastatic HGSC cells is driven by a chemotherapy-independent ER stress response and culminates with the establishment of a local immune contexture characterized by T_H_1 polarization and cytotoxic activity that enables superior clinical benefits.

**Conclusions:**

Our data indicate that CALR levels in primary and metastatic HGSC samples have robust prognostic value linked to the activation of clinically-relevant innate and adaptive anticancer immune responses.

## Introduction

It is now accepted that tumors form, progress and respond to therapy in the context of an intimate, bidirectional interaction with the immune system [1, 2]. In this context, malignant cells progressively escape immunosurveillance by losing their antigenicity, i.e., the exposure on the cell surface of antigens not covered by central thymic tolerance [3, 4] and adjuvanticity, i.e., the emission of immunostimulatory signals through molecules commonly known as damage-associated molecular patterns (DAMPs) [5, 6]. In physiological conditions, DAMPs are sequestered in the intracellular microenvironment, where they cannot be detected by the host immune system [5, 6]. However, cells experiencing sub-lethal or lethal stress conditions passively release, actively secrete, or expose on the outer leaflet of the plasma membrane, several DAMPs, hence enabling the latter to mediate a variety of immunomodulatory functions [7–9].

Endoplasmic reticulum (ER) chaperones including calreticulin (CALR) and various heat-shock proteins (HSPs) are well known for their key role as pro-phagocytic DAMPs in the successful activation of anticancer immunity by malignant cells undergoing immunogenic cell death [5, 6]. In line with this notion, high expression levels of CALR and/or CALR exposure on the membrane of cancer cells have been linked with superior disease outcome in patients with colorectal carcinoma (CRC) [10], non-small cell lung carcinoma (NSCLC) [11, 12], acute myeloid leukemia (AML) [13], and ovarian cancer [11] generally in association with improved anticancer immunity. Conversely, the impact of CALR levels on the composition and functional orientation of the HGSC microenvironment remain unclear.

Here, we investigated the impact of CALR levels on disease outcome in a retrospective cohort of 152 patients with resectable high-grade serous carcinoma (HGSC) who did not receive neoadjuvant chemotherapy. Our data suggest that increased CALR levels in both primary and metastatic tumor tissues are associated with superior disease outcome linked to the establishment of a tumor microenvironment (TME) exhibiting T_H_1 polarization and activation of immune effectors.

## Materials and methods

### Patients

***Study group 1.*** Two retrospective series of 152 primary and 74 metastatic formalin-fixed paraffin-embedded (FFPE) samples were obtained from patients with HGSC who underwent surgery without neoadjuvant chemotherapy between 2008 and 2014 at University Hospital Hradec Kralove (Czech Republic). Baseline characteristic of these patients are summarized in Table 1. From those 24 patients samples were further analyzed using RNA-seq technology. ***Study group 2*.** A retrospective cohort of 45 patients with HGSC who received neoadjuvant chemotherapy followed by curative resection between 2008 and 2014 was obtained from University Hospital Hradec Kralove (Czech Republic). Baseline characteristics of these patients are summarized in Additional file 1: Table S1. ***Study group 3*.** An additional series of 35 samples from HGSC patients who did not receive neoadjuvant chemotherapy was prospectively collected at Hospital Motol (Czech Republic). Written informed consent was obtained from the patients before inclusion in the prospective study. The protocol was approved by the local ethics committee. Baseline characteristic of these patients are summarized in Additional file 1: Table S2. Pathologic staging was performed according to the 8th TNM classification (2017), and histologic types were determined according to the current WHO classification [14, 15]. Data on long-term clinical outcome were obtained retrospectively by interrogation of municipality registers or patients’ families. The experimental design of the study is summarized in Additional file 1: Figure S1.

**Table 1:** Main clinicopathological features of Study Group 1

Variable	Study Group 1
	(*n* = 152)
Age:	
Mean age ± SEM	65 ± 0.81
Range	41–85
pTNM stage:	
Stage I	20 (13.2%)
Stage II	11 (7.2%)
Stage III	111 (73%)
Stage IV	10 (6.6%)
Debulking	
R0	75 (49.4%)
R1	12 (7.9%)
R2	65 (42.7%)
Vital status of patients	70 (46.1%)

### Immunohistochemistry

Tumor specimens from Study Group 1 and Study Group 2 were fixed in neutral buffered 10% formalin solution and embedded in paraffin as per standard procedures. Immunostaining with antibodies specific for lysosomal associated membrane protein 3 (LAMP3; best known as DC-LAMP), CD8, CD20, CALR and natural cytotoxicity triggering receptor 1 (NCR1; best known as NKp46) (Additional file 1: Table S3) was performed according to conventional protocols. Briefly, tissue sections were deparaffinized and rehydrated descending alcohol series (100, 96, 70, and 50%), followed by antigen retrieval with Target Retrieval Solution (Leica) in EDTA pH 8.0 (for DC-LAMP/CD20, CD8, NKp46) or in citrate buffer at pH 6.0 (for CALR), in preheated water bath (97 °C, 30 min). Sections were allowed to cool down to RT for 30 min, and endogenous peroxidase was blocked with 3% H_2_O_2_ for 15 min. For co-staining, endogenous alkaline phosphatase was blocked by levamisole (Vector). Sections were then treated with protein block (DAKO) for 15 min and incubated with primary antibodies, followed by the revelation of enzymatic activity. Images were acquired using a Leica Aperio AT2 scanner (Leica).

### Scoring

CALR expression in the tumor microenvironment was quantified as a function of CALR^+^ positive tumor cells, as published previously [12]. Scores were calculated on 10 different fields visually inspected at 20x magnification under a light microscope (DM2000LED; Leica), and classified into (1) score 1, < 10% CALR^+^ cells; (2) score 2, 10–25% CALR^+^ cells, (3) score 3, 26–50% CALR^+^ cells; (4) score 4, 51–75% CALR^+^ cells; and (5) score 5, > 75% positive cells (Additional file 1: Figure S2.). Quantification was done performed by two independent observers (LK, JF) and reviewed by two expert pathologists (JL, PS). DC-LAMP^+^, CD8^+^, CD20^+^ and NKp46^+^ cells were quantified in the tumor stroma and tumor nests of the whole tumor sections with Calopix (Tribvn). Data are reported as absolute number of positive cells/mm^2^ (for DC-LAMP^+^, CD8^+^ and NKp46^+^ cells) or cell surface/total tumor section surface (for CD20^+^ cells), as previously described [16–19]. Immunostaining and quantifications were reviewed by at least three independent observers (IT, LK, JF, PS, JL) and two expert pathologists (JL, PS).

### Flow cytometry

As previously described, fresh ovarian tumor specimens were minced with scissors, digested in PBS containing 1 mg/ml of Collagenase D (Roche) and 0.2 mg/ml DNase I at 37 °C for 30 min mechanically dissociated using the gentleMACS dissociator (Miltenyi Biotec) and passed through a 70 μm nylon cell strainer (BD Biosciences) [16]. To determine the ecto-CALR exposure, mononuclear cells were stained with primary antibodies against CD45, cytokeratin, human epithelial antigen, CD227 to distinguish the population of leukocytes and malignant cells, and antibodies against CALR or isotype control (Additional file 1: Table S4) for 20 min at 4 °C in the dark, following by washing and acquisition on a Fortessa flow cytometer (BD Bioscience). Flow cytometry data were analyzed with the FlowJo software (TreeStar). Gating strategy is depicted in Additional file 1: Figure S3.

### Degranulation and IFN-γ production after in vitro stimulation

Mononuclear cells isolated from fresh tumor specimens were stimulated with 50 ng/ml of phorbol 12-myristate 13-acetate (PMA) + 1 μg/ml of ionomycin for 1 h followed by 3 h incubation with brefeldin A (BioLegend). Unstimulated cells were used as a control. The cells were then washed in PBS, stained with anti-CD3 Alexa Fluor 700 (EXBIO), anti-CD4 ECD (Beckman Coulter) and anti-CD8 HV500 (BD Biosciences), fixed using fixation/permeabilization buffer (eBioscience), permeabilized with permeabilization buffer (eBioscience) and intracellularly stained with an anti-IFN-γ PE-Cy7 (eBioscience), anti-granzyme B Brilliant Violet 421 (BD Biosciences) (Additional file 1: Table S4). The percentage of CD3^+^CD8^+^ T cells producing IFN-γ and degranulating upon PMA/ionomycin stimulation were determined by flow cytometry. The data were analyzed with the FlowJo software package (Tree Star, Inc.). Gating strategy is depicted in Additional file 1: Figure S4.

### TCGA data analysis

Patients with HGSC (*n* = 302) were identified in The Cancer Genome Atlas (TCGA) public database (https://cancergenome.nih.gov/). Differentially expressed genes (DEGs) between the *CALR*^*Hi*^ and *CALR*^*Lo*^ groups were determined using the LIMMA-R package [20]. Hierarchical clustering analysis was conducted using the ComplexHeatmap package, based on the Euclidean distance and complete clustering method [21]. Immune analyses were performed using ClueGo [22]. The MCP-counter R package was used to estimate the abundance of tissue-infiltrating immune cell populations (Additional file 1: Table S5) [23].

### Statistical analysis

Survival analysis was performed using the R package survival analysis. The overall prognostic value of continuous variables was assessed (1) by Wald tests for univariate COX regression models, (2) by log-rank tests using median-based cutoffs. The prognostic value of CALR and immune density was assessed by multivariate Cox regression. Student’s *t* tests, Wilcoxon tests and Mann-Whitney tests were used to assess statistical significance, *p* values are reported (considered not significant when > 0.05).

## Results

### Prognostic impact of CALR expression in TME of primary and metastatic HGSC

**Fig. 1:**
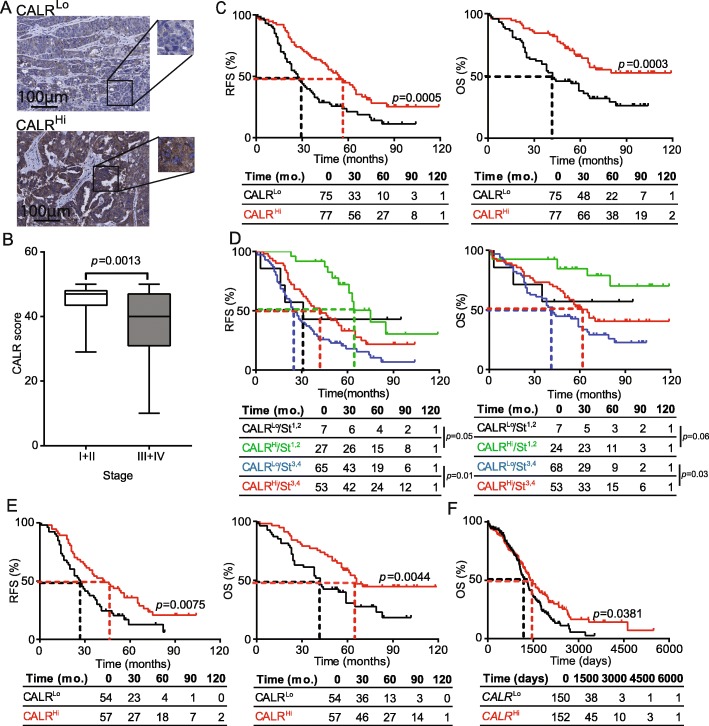
Prognostic impact of CALR expression in the primary TME of HGSC patients. **a** Representative images of CALR immunostaining in CALR^Lo^ and CALR^Hi^ patients. Scale bar = 100 μm. **b** CALR expression levels among different pathological disease stages. Box plots: lower quartile, median, upper quartile; whiskers, minimum, maximum. RFS (**c**) and OS (**d**) of 152 HGSC patients who did not receive neoadjuvant chemotherapy, upon stratification based on median CALR expression. **d** RFS and OS of 152 HGSC patients who did not receive neoadjuvant chemotherapy, upon stratification based on median CALR expression and stage. **e** RFS and OS of 111 HGSC patients stage III who did not receive neoadjuvant chemotherapy, upon stratification based on median CALR expression (**f**) OS of 302 HGSC patients from the TCGA public database upon stratification based on median *CALR* expression. Survival curves were estimated by the Kaplan-Meier method, and difference between groups were evaluated using log-rank test. Number of patients at risk are reported

Primary tumor (PT) samples from a retrospective series of 152 patients with HGSC who did not receive neoadjuvant chemotherapy (Study Group 1) (Table 1) were analyzed for CALR expression by immunohistochemistry (IHC) (Fig. 1a). CALR levels were rather heterogeneous within samples from the same TNM stage, with a trend for decreased CALR expression in Stage III-IV lesions that was statistically significant as compared to Stage I-II lesions (*p* = 0.0013) (Fig. 1b). To evaluate the prognostic impact of CALR expression in primary HGSC tissues, we investigated relapse-free survival (RFS) and overall survival (OS) upon stratifying the entire patient cohort based on the median CALR expression score. We found that CALR^Hi^ patients had a significantly improved RFS and OS as compared with their CALR^Lo^ counterparts (median RFS: 54 mo. versus 27 mo.; *p* = 0.0005; median OS; > 120 mo. versus 42 mo.; *p* = 0.0003) (Fig. 1c). As CALR levels tend to correlate with disease stage and both these factors have prognostic significance (Fig. 1d, Additional file 1: Figure S5A), we harnessed univariate and multivariate Cox regression models to demonstrate that such significance is mutually independent (Tables 2 and 3). Consistent with this, survival curves of the patient cohort stratified for stage (I,II versus III/IV) and CALR expression (CALR^Lo^ versus CALR^Hi^) documented significantly improved OS for CALR^Hi^/Stage^III,IV^ patients over their CALR^Lo^/Stage^III,IV^ counterparts (*p* = 0.03) (Fig. 1d). A similar trend not reaching statistical significance (potentially due to the limited amount of patients in this subset) was observed for CALR^Hi^/Stage^I,II^ patients compared to their CALR^Lo^/Stage^I,II^ counterparts (*p* = 0.06) (Fig. 1d). RFS data further comforted these findings (Fig. 1d). We therefore decided to focus on patients with Stage III HGSC (*n* = 111), the majority of patients from Study Group 1, to remove the potential confounding effect linked to disease stage, thus eliminating patients at other stages from further analyses. Importantly, CALR levels in both PT (Fig. 1e) and metastatic tumors (MT) (Additional file 1: Figure S5B) were significantly associated with improved RFS and OS (median RFS PT: 43 mo. versus 27 mo.; *p* = 0.0075; median OS PT; 66 mo. versus 42 mo.; *p* = 0.0044; median RFS MT: 41.5 mo. versus 21 mo.; *p* = 0.01; median OS MET; > 120 mo. versus 34 mo.; *p* = 0.0012). Both univariate and multivariate Cox analyses confirmed the prognostic impact of CALR levels in patients with Stage III HGSC (Tables 2 and 3). To validate these findings in a larger patient cohort, we analyzed the prognostic role of *CALR* mRNA levels in 302 patients with primary ovarian cancer from The Cancer Genome Atlas (TCGA) database, based on the median cutoff approach [12, 13]. High intratumoral *CALR* mRNA levels were strongly associated with improved OS (*p* = 0.0381) (Fig. 1f). Altogether, these results demonstrate that CALR expression in both primary and metastatic lesions constitutes a strong prognostic biomarker for the identification of chemotherapy-naïve HGSC patients with favorable disease outcome upon tumor resection.

**Table 2:** Univariate Cox proportional hazard analysis

Variable	Overall survival		Relapse-free survival	
	HR (95% Cl)	*p*	HR (95% Cl)	*p*
CA125	1 (1–1)	0.14	1 (1–1)	0.31
Stage		0.55		0.8
Stage I	1		1	
Stage II	0.61 (0.12–3.14]	0.52	1.14 (0.4–3.19)	0.808
Stage III	2.99 (1.21–7.43]	**0.018**	2.64 (1.33–5.26)	**0.006**
Stage IV	5.35 (1.63–17.57)	**0.006**	4.84 (1.79–13.09)	**0.002**
Debulking		0.22		0.08
Debulking R0	1		1	
Debulking R1	1.67 (0.73–3.8)	0.224	1.84 (0.92–3.67)	0.084
Debulking R2	2.17 (1.36–3.47)	**0.001**	2.76 (1.85–4.13)	**< 0.0001**
Age	1 (0.98–1)	0.73	1 (0.99–1)	0.48
CALR	0.96 (0.94–0.98)	**< 0.0001**	0.97 (0.95–0.98)	**< 0.0001**
DC-LAMP summary	0.86 (0.76–0.96)	**0.0097**	0.98 (0.94–1)	0.19
CD8 summary	1 (1–1)	**0.011**	1 (1–1)	0.057
CD20	0.2 (0.039–0.97)	**0.046**	0.5 (0.23–1.1)	0.087
NKp46	1 (0.82–1.2)	0.98	0.91 (0.73–1.1)	0.42

**Table 3:** Multivariate Cox proportional hazard analysis

Variable	Overall survival		Relapse-free survival	
	HR (95% Cl)	*p*	HR (95% Cl)	p
CA125	1 (1–1)	0.495	1 (1–1)	0.98
Stage		0.5		0.98
Stage I	1		1	
Stage II	0.76 (0.14–4.18)	0.755	1.3 (0.45–3.77)	0.627
Stage III	2.21 (0.76–6.41)	0.145	1.75 (0.79–3.87)	0.165
Stage IV	4.89 (1.33–17.9)	**0.017**	2.84 (0.96–8.4)	0.06
Debulking		0.39		0.9
Debulking R0	1		1	
Debulking R1	0.62 (0.21–1.84)	0.386	0.95 (0.4–2.24)	0.91
Debulking R2	1.35 (0.8–2.27)	0.262	1.92 (1.21–3.06)	**0.006**
Age	1 (0.98–1.03)	0.743	1.01 (0.99–1.03)	0.508
CALR	0.96 (0.94–0.99)	**0.0003**	0.97 (0.96–0.99)	**0.002**
DC-LAMP summary	0.8 (0.7–0.91)	**0.001**	0.97 (0.94–1.0)	0.12
CD8 summary	0.99 (0.99–0.99)	**0.0007**	0.99 (0.99–1)	0.13
CD20	0.23 (0.3–1.54)	0.13	1 (0.33–1.5)	0.86
NKp46	1.08 (0.9–1.3)	0.37	1.11 (0.93–1.35)	0.24

### CALR levels in HGSC correlate with signs of an ongoing ER stress response

**Fig. 2:**
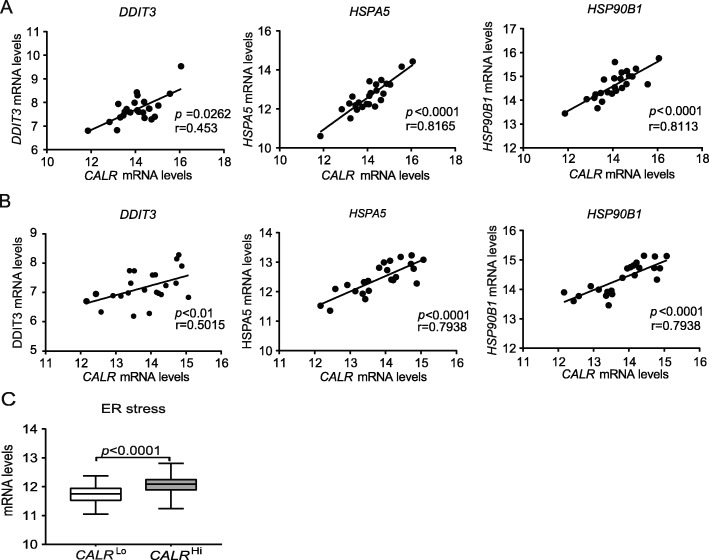
CALR exposure correlate with robust intracellular stress response in the TME. Correlation between *CALR* mRNA levels and *DDIT3, HSPA5*, or *HSP90B1* mRNA levels in PT (**a**) and MT (**b**) samples of 24 patients with HGSC from study group 1 and in (**c**) 302 patients with HGSC from TCGA public database. Box plots: lower quartile, median, upper quartile; whiskers, minimum, maximum

CALR expression on the surface of cells undergoing ICD relies on the activation of the ER stress response in dying cells [24, 25]. We therefore checked whether the mRNA levels encoding 3 distinct components of the canonical ER stress response, namely DNA damage inducible transcript 3 (DDIT3, best known as CHOP), heat shock protein family A (Hsp70) member 5 (HSPA5, best known as BIP), and heat shock protein 90 beta family member 1 (HSP90B1) [26], would correlate with *CALR* mRNA levels in samples from Study Group 1. We observed a statistically significant positive correlation between *CALR* levels and *DDIT3*, *HSPA5* and *HSP90B1* in both PT and MT samples (Fig. 2a and b). To validate our findings in an independent patient cohort, we retrieved normalized expression data on *DDIT3*, *HSPA5* and *HSP90B1*, as well as on transcripts encoding the ER stress-relevant proteins activating transcription factor 6 (ATF6) and X-box binding protein 1 (XBP1) for 302 patients with primary ovarian cancer from the TCGA database, and analyzed their correlation with *CALR* abundance. Also in this setting, *DDIT3*, *HSPA5, HSP90B1*, *ATF6*, and *XBP1* levels exhibited a highly significant positive correlation with *CALR* expression (Fig. 2c), corroborating the notion that ovarian cancer cells are subjected to ER stress irrespective of treatment, resulting in spontaneous CALR upregulation in a majority of patients. Next, we decided to evaluate the potential impact of platinum- and paclitaxel-based chemotherapy, which is a common standard of care for patients with ovarian cancer [27], on the adjuvanticity of HGSC cells. To this aim, we analyzed CALR expression in PT samples from an independent cohort of 45 patients who received neoadjuvant chemotherapy before surgery (Study Group 2) (Additional file 1: Table S1). We observed no difference in CALR levels in PT samples from chemotherapy-naïve patients versus patients who underwent neoadjuvant chemotherapy (Additional file 1: Figure S5C). Moreover, OV90 ovarian cancer cells exposed to carboplatin plus paclitaxel for 24 h failed to manifest increased CALR exposure on the plasma membrane, at odds with OV90 cancer cells exposed to idarubicin (an anthracycline that triggers ICD) (Additional file 1: Figure S5D). Taken together, these findings lend further support to the notion that HGSC cells are exposed to microenvironmental conditions that favor CALR upregulation irrespective of chemotherapy.

### High CALR levels are associated with a T_H_1-polarized, cytotoxic CD8^+^ T-cell response

**Fig. 3:**
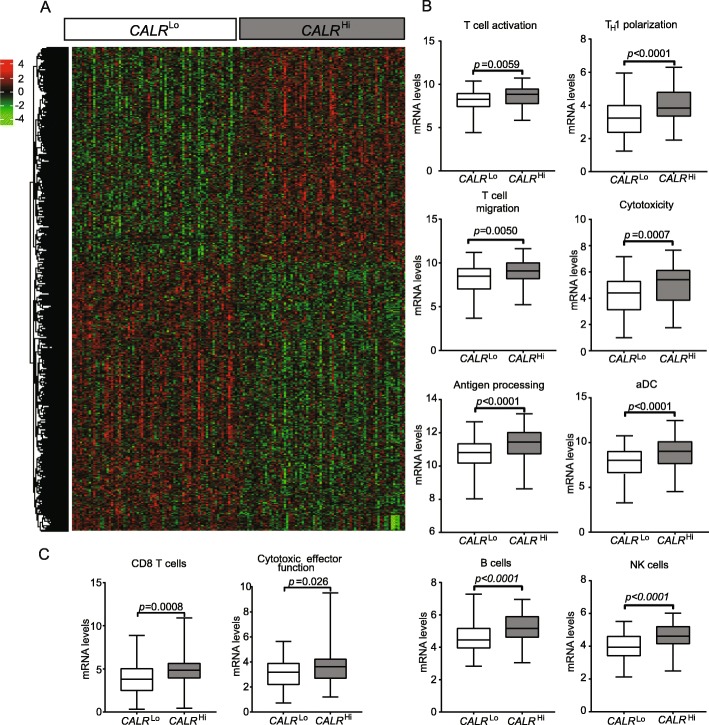
Transcriptional signatures of the tumor microenvironment of CALR^Hi^ versus CALR^Lo^ HGSCs. **a** Hierarchical clustering of significantly upregulated and downregulated genes in 77 *CALR*^Hi^ versus 77 *CALR*^Lo^ HGSC patients from the TCGA public database (302 patients were divided into 4 groups using quartile stratification, only lower (no = 77) and upper (no = 77) quartile is presented). **b** Relative expression levels of genes linked to T cells activation, T_H_1 polarization, T cell migration, cytotoxicity, antigen processing, activated DCs (aDCs), B cells and NK cells in 77 *CALR*^Hi^ versus 77 *CALR*^Lo^ TCGA HGSCs (302 patients were divided into 4 groups using quartile stratification, only lower (no = 77) and upper (no = 77) quartile is presented). Box plots: lower quartile, median, upper quartile; whiskers, minimum, maximum. **c** Relative abundance of CD8^+^ T cells and cytotoxic effector functions across 77 *CALR*^Hi^ and 77 *CALR*^Lo^ TCGA HGSCs (302 patients were divided into 4 groups using quartile stratification, only lower (no = 77) and upper (no = 77) quartile is presented). Box plots: lower quartile, median, upper quartile; whiskers, minimum, maximum

To characterize the impact of CALR expression on the composition and functional polarization of the HGSC immune infiltrate, we compared transcriptional signatures of 77 *CALR*^Hi^ patients and 77 *CALR*^Lo^ patients from the TCGA database. We identified a set of 1563 genes that were significantly over-represented in *CALR*^Hi^ PTs as compared to their *CALR*^Lo^ counterparts (Fig. 3a) (Additional file 1: Table S6). Bioinformatic analyses revealed a strong association between such DEGs and T cell activation, T_H_1 polarization, T cell migration, cytotoxicity, antigen processing, dendritic cell (DC) activation as well as B and natural killer (NK) cell function (Fig. 3b and Additional file 1: Figure S6A; Table S7). Alongside, we used the MCP-counter R package to estimate the relative abundance of different immune cell population in the TME of *CALR*^Hi^ versus *CALR*^Lo^ patients. Compared to their *CALR*^Lo^ counterparts, *CALR*^Hi^ PTs exhibited were enriched in gene sets specific for CD8^+^ T cells (*p* = 0.008) and cytotoxic effector functions (*p* = 0.026) (Fig. 3c; Additional file 1: Table S5). To further characterize the impact of CALR expression on the composition of the immune infiltrate in HGSC metastases, we used RNAseq to characterize the expression profile of 13 CALR^Lo^ versus 11 CALR^Hi^ patients from Study Group 1. We identified a set of 406 genes that were significantly overrepresented in samples from CALR^Hi^ patients as compared to their CALR^Lo^ counterparts (Additional file 1: Figure S6B). Bioinformatic analyses revealed a strong association between such DEGs with B cell-dependent immunity and complement activation (Additional file 1: Figure S6C). Thus, in both primary and metastatic HGSC samples, high CALR levels are associated with biomarkers of a T_H_1-polarized, cytotoxic immune response.

### CALR expression is associated with HGSC infiltration by activated DCs and B cells

**Fig. 4:**
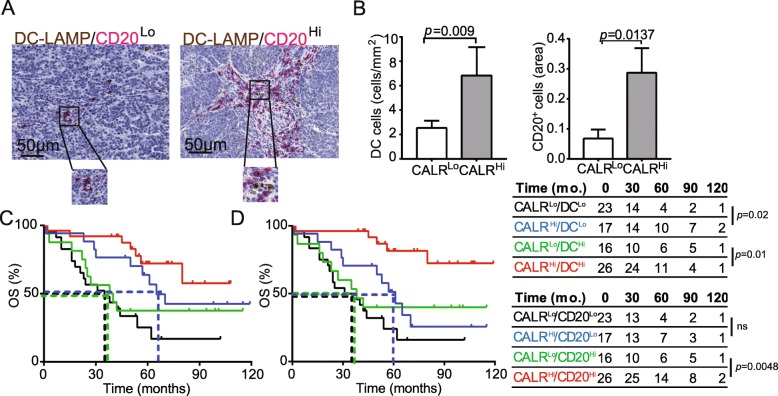
CALR expression positively correlate with the frequency of mature DC-LAMP^+^ DCs and CD20^+^ B cells. **a** Representative images of DC-LAMP and CD20 immunostaining. Scale bar = 50 μm. **b** Density of DC-LAMP^+^ cells and CD20^+^ B cells in TME of CALR^Lo^ versus CALR^Hi^ HGSCs (*n* = 82). Box plots: lower quartile, median, upper quartile; whiskers, minimum, maximum. OS of HGSC patients (study group 1) who did not receive neoadjuvant chemotherapy, upon stratification based on median expression of CALR and density of DC-LAMP^+^ cells (**c**) or CD20^+^ B cells (**d**)

Surface-exposed CALR acts as a pro-phagocytic signal for antigen-presenting cells (APCs), promoting the efficient uptake of dying cells in the context of immunostimulatory signals [28]. As we observed a positive correlation between *CALR* levels and the levels of several transcripts associated with DC and B cell activation (Fig. 3b), we set to evaluate the abundance of mature DC-LAMP^+^ DCs and CD20^+^ B cells in PT lesions from HGSC patients (Fig. 4a). We found a higher density of mature DC-LAMP^+^ DCs and CD20^+^ B cells in the TME of CALR^Hi^ patients compared to their CALR^Lo^ counterparts (DC-LAMP: *p* = 0.009; CD20: *p* = 0.0137) (Fig. 4B). Using biomolecular analyses, we demonstrated that the expression of C-C motif chemokine ligand 4 (*CCL4*), *CCL5*, *CCL7, CCL8, CCL13, CCL23, CCL25* and C-X-C motif chemokine ligand 5 (*CXCL5*), *CXCL6, CXCL9, CXCL10, CXCL11, CXCL13 and CXCL17* is more pronounced in *CALR*^*Hi*^ samples as compared to their *CALR*^*Lo*^ counterparts (Additional file 1: Figure S7A). Bioinformatic analyses revealed that such DEGs are mainly involved in tumor infiltration by lymphocytes and leukocytes chemotaxis and migration (Additional file 1: Figure S7B). Tumor infiltration by mature DC-LAMP^+^ DCs and CD20^+^ B cells impact disease outcome in chemotherapy-naïve patients with HGSC undergoing surgical tumor resection [16]. Indeed, stratifying patients from Study Group 1 into four subsets based on CALR score and the frequency of tumor-infiltrating DC-LAMP^+^ DCs (Fig. 4c) or CD20^+^ B cells (Fig. 4d) revealed a superior survival for CALR^Hi^ patients as compared to their CALR^Lo^ amongst all patients subgroups (DC-LAMP^Hi^: *p* = 0.01; DC-LAMP^Lo^: *p* = 0.02; CD20^Hi^: *p* = 0.0048; CD20^Lo^: *p* = 0.06). These results suggest that CALR expression can be harnessed to ameliorate the prognostic stratification of patients with HGSC based on DC-LAMP and CD20 only.

### CALR levels are associated with HGSC infiltration by IFN-γ producing CD8^+^ T cells

**Fig. 5:**
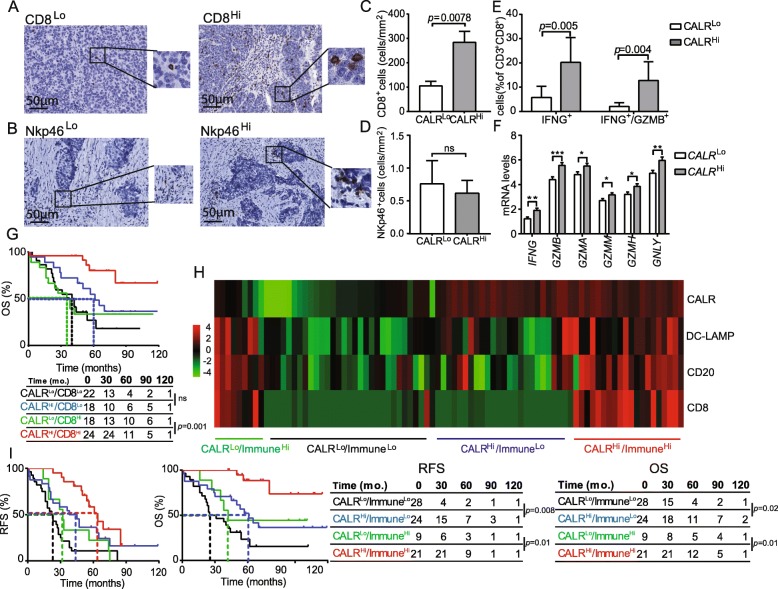
Impact of CALR on the frequency and cytotoxicity of CD8 T cells in HGSC and immune contexture of HGSC. Representative images of CD8 (**a**) and Nkp46 (**b**) immunostaining. Scale bar = 50 μm. Density of CD8^+^ (**c**) and NK (**d**) cells in TME of CALR^Lo^ versus CALR^Hi^ HGSCs (*n* = 82). Box plots: lower quartile, median, upper quartile; whiskers, minimum, maximum. **e** Percentage of IFN-γ^+^ and IFN-γ^+^ /GZMB^+^ cells among CD8^+^ T cells from the HGSC of 17 CALR^Lo^ and 18 CALR^Hi^ patients after non-specific stimulation. Box plots: lower quartile, median, upper quartile; whiskers, minimum, maximum. **f** Expression levels of *IFNG, GZMB, GZMA, GZMM, GZMH, GNLY* in *CALR*^*Hi*^ patients from the TCGA database as compared to their *CALR*^*Lo*^ counterparts. (302 patients were divided into 4 groups using quartile stratification, only lower (no = 77) and upper (no = 77) quartile is presented). Box plots: lower quartile, median, upper quartile; whiskers, minimum, maximum. **g** OS of HGSC patients (study group 1) who did not receive neoadjuvant chemotherapy, upon stratification based on median expression of CALR and density of CD8^+^ cells. Survival curves were estimated by the Kaplan-Meier method, and difference between groups were evaluated using log-rank test. Number of patients at risk are reported. **h** Clustering of HGSC patients from study group 1 based on median stratification of CALR expression and median densities of DC-LAMP^+^, CD8^+^ and CD20^+^ cells as determined by immunohistochemistry. **i** RFS and OS of HGSC patients from study group 1 who did not receive neoadjuvant chemotherapy, upon stratification based on median expression of CALR and median density of immune infiltrate as indicated by clustering heatmap. Survival curves were estimated by the Kaplan-Meier method, and differences between groups were evaluated using log-rank test. Number of patients at risk are reported

CALR expression has been positively correlated with CD8^+^ T cell infiltration in multiple human tumors, but not HGSC [25]. Moreover, little is known on the potential links between CALR levels and tumor infiltration by NK cells [29]. Driven by these premises and by the transcriptional signature of *CALR*^Hi^ versus *CALR*^Lo^ patients, we decided to investigate PT and MT samples from Study Group 1 for CD8^+^ T cell and NK cell infiltration by IHC (Fig. 5a, b). We observed a higher density of CD8^+^ T cells in PT samples from CALR^Hi^ patients as compared to the their CALR^Lo^ counterparts (*p* = 0.0078) (Fig. 5c). A similar trend that failed to reach statistical significance was documented for MT samples (Additional file 1: Figure S8A). Conversely, CALR expression had no impact on the abundance of NK cells in PT (Fig. 5d) and MT (Additional file 1: Figure S8B) samples. To address the functional capacity of CD8^+^ T cells from the TME of CALR^Hi^ versus CALR^Lo^ patients, we used flow cytometry on freshly resected PTs. Non-specific stimulation caused a more pronounced increase in CD8^+^ T cells staining positively for the effector molecule interferon gamma (IFNG, best known as IFN-γ) alone (*p* = 0.005) or together with the cytolytic enzyme granzyme B (GZMB) (*p* = 0.004) in CALR^Hi^ versus CALR^Lo^ samples (Fig. 5e). In line with this notion, the mRNA levels of *IFNG, GZMB*, *GZMA*, *GZMM*, *GZMH*, and granulysin (*GNLY*, coding for yet another effector molecule of T cells) are higher in *CALR*^Hi^ patients from the TCGA database as compared to their *CALR*^Lo^ counterparts (Fig. 5f). Univariate and multivariate Cox analyses confirmed prior observations from us and others [16, 30] indicating that high densities of CD8^+^ T cells have a positive impact on the OS of patients with HGSC (Tables 2 and 3). Next, we assessed the combined prognostic impact of CALR expression and CD8^+^ T cells by stratifying patients from Study Group 1 based on CALR score and median CD8^+^ T cell density into 4 subgroups (CALR^HI^/CD8^Hi^, CALR^Lo^/CD8^Hi^, CALR^Hi^/CD8^Lo^; CALR^Lo^/CD8^Lo^). We were unable to document a statistically significant difference in the survival of CALR^Hi^/CD8^Lo^ patients as compared to their CALR^Lo^/CD8^Lo^ counterparts (Fig. 5g). However, CALR^Hi^/CD8^Hi^ patients had a robust survival advantage over their CALR^Lo^/CD8^Hi^ counterparts (*p* = 0.001) (Figs. 5g), indicating that CALR expression can be employed to identify HGSC patients with extensive tumor infiltration by CD8^+^ T cells but relatively poor disease outcome.

As we observed a positive correlation between CALR levels and tumor infiltration by diverse immune cell subsets, we next evaluated the global immunological profile of the TME of CALR^Lo^ versus CALR^Hi^ PT samples from Study Group 1 by IHC. This approach identified 4 different clusters of patients corresponding to high versus low CALR expression in the context of elevated versus reduced tumor infiltration by DC-LAMP^+^ mature DCs, CD20^+^ B cells and CD8^+^ T cells (Immune^Hi^ and Immune^Lo^, respectively) (Fig. 5h). Importantly, CALR status improved the prognostic assessment on RFS and OS amongst both Immune^Hi^ (RFS: *p* = 0.01; OS: *p* = 0.01) and Immune^Lo^ (RFS: *p* = 0.008; OS: *p* = 0.02) patient subgroups (Fig. 5i). Altogether, our findings document a robust independent prognostic value for CALR levels of chemotherapy-naïve patients with HGSC, linked to the impact of CALR on the establishment of a T_H_1-polarized TME that supports anticancer immunity.

## Discussion

Despite recent developments in diagnostic and treatment modalities leading to an improvement in the short-term survival of patients with ovarian cancer, most of patients are diagnosed at advanced stage of the disease with metastatic spreading, due to the non-specific symptoms and the absence of effective screening methods [31]. Therefore, there is an urgent need for new diagnostic, including prognostic and predictive biomarkers and therapeutic tools for a clinical management of cancer patients, which still represents the principal cause of mortality from gynecologic malignancies. Accumulating preclinical and clinical evidence indicates that DAMPs and DAMP-associated processes impact disease outcome in patients with various malignancies [25]. In particular, the prognostic relevance of CALR expression levels or exposure on the membrane of cancer cells has been investigated by us and others in the context of multiple malignancies [10–13, 32–34]. Nevertheless, the influence of CALR levels on the composition and functional orientation of the immune infiltrate of HGSCs and their link with disease outcome in chemotherapy-naïve patients remain have not been elucidated until now.

As documented in numerous in vitro and in vivo models, ecto-CALR serves as a signal to facilitate the engulfment of tumor cells by DCs, which leads to tumor antigen presentation and stimulation tumor-specific cytotoxic T lymphocytes responses [35, 36]. Here, we analyzed 3 different cohorts of primary and metastatic samples from patients with HGSCs who did not receive chemotherapy prior to tumor resection. By combining IHC and biomolecular analyses, we demonstrated that a high CALR expression is strongly associated with higher density of both mature DC-LAMP^+^ DCs and CD20^+^ B cells resulting in T_H_1-polarized immune contexture that acquired effector functions. These findings recapitulate previous findings by us and others demonstrating that CALR exposure by neoplastic cells is associated with increased tumor infiltration by myeloid cells and effector memory CD8^+^ T cells in patients with NSCLC [12], increased frequency of T cells in TME of colorectal carcinoma [10] and increased proportion of LAA-specific CD4^+^ and CD8^+^ T cells in patients with AML [13]. Moreover, here we observed correlation between high CALR expression in the TME and higher cytotoxic functions of effector tumor infiltrating CD8^+^ T cells and NK cells, although the number of later population was not significantly increased in CALR^Hi^ patients, suggesting the impact of CARL exposure on enhanced NK cell cytotoxic and secretory functions. These results are in line with our recent findings demonstrating that spontaneous CALR exposure on malignant blasts supports innate anticancer immunity by NK cells via and indirect mechanism relying on myeloid CD11c^+^CD14^+^ cells resulting in overall superior survival of AML patients [37, 38]. Altogether, we demonstrated that high CALR levels bear independent positive prognostic value and hence can be harnessed to improve patient stratification based on previously identified factors including DC-LAMP^+^ DC, CD20^+^ B cell and CD8^+^ T cell infiltration. These findings extend previous data by us and others on the improved immunological functions linked to increased CALR levels in the context of AML [13], NSCLC [12] and CRC [10].

We also demonstrate that CALR is expressed by HGSC cells independent of standard-of-care chemotherapy, possibly reflecting malignant transformation itself [39] and/or the limited immunogenicity of carboplatin-based chemotherapy [40]. Accordingly, we identified a robust correlation between CALR expression and 3 distinct genes involved in ER stress responses in two independent HGSC patient cohorts. Similar observations have been made by us and others in the context of AML [13, 41] and NSCLC [12]. Interestingly, we also identified a significant decreased in CALR expression in samples from advanced stages of disease, which is in line with the notion that progressing tumors tend to lose both antigenicity and adjuvanticity [3, 5, 42].

In conclusion, CALR stand out as a robust prognostic biomarker for chemotherapy-naïve patients with HGSC. It can be speculated that CALR^Lo^ patients would benefit from neoadjuvant or adjuvant chemotherapeutic regimens that are known to drive robust ER stress responses in the context of ICD, such as oxaliplatin, doxorubicin and other anthracyclines [6]. As ovarian cancer still represents one of the top 5 leading causes of cancer-related death amongst women in the US (source https://www.cdc.gov/cancer/uscs/index.htm), clinical trials specifically addressing this possibility are urgently awaited.

## Supplementary information

**Additional file 1: Figure S1.** Experimental design of the study. **Figure S2.** Representative images of CALR immunostaining. Scale bar = 100 μm. **Figure S3.** Flow cytometry-assisted quantification of surface exposed CALR. **Figure S4**. Degranulation and IFN-γ production after in vitro stimulation. **Figure S5.** Prognostic impact of CALR expression in the metastatic TME of HGSC patients and impact of chemotherapy on the final CALR exposure. **Figure S6.** Transcriptional signatures of the tumor microenvironment of CALR^Hi^ versus CALR^Lo^ PT and MT samples of HGSCs patients. **Figure S7.** Chemokine signatures of the tumor microenvironment of CALRHi versus CALRLo of HGSCs patients. **Figure S8.** Impact of CALR on the frequency of CD8^+^ T cells and NKp46^+^ NK cells in MT samples of HGSC patients. **Table S1.** Main clinical and biological characteristics of 45 HGSC patients after neo-adjuvant chemotherapy treatment (study group 2) (University Hospital Hradec Kralove). **Table S2.** Main clinical and biological characteristics of 35 HGSC patients without neo-adjuvant chemotherapy treatment prospectively collected (study group 3) (University Hospital Motol). **Table S3.** The list of antibodies use for IHC staining. **Table S4.** The list of antibodies used for flow cytometry. **Table S5.** The list of genes used by MCP counter for identification of distinct cell populations. **Table S6.** List of genes significantly overrepresented in CALR^Hi^ versus CALR^Lo^ HGSC samples from TCGA public database. **Table S7.** List of genes in boxplot significantly overrepresented in CALR^Hi^ versus CALR^Lo^ HGSC samples from TCGA public database.

## Data Availability

The datasets used and/or analysed during the current study are available from the corresponding author on reasonable request.
