# Supplementary material for: Calreticulin exposure correlates with robust adaptive antitumor immunity and favorable prognosis in ovarian carcinoma patients
Source: J Immunother Cancer. 2019 Nov 20;7:312. doi: 10.1186/s40425-019-0781-z (PMC6868694; doi:10.1186/s40425-019-0781-z)
Supplement: Supplementary file 1 — Additional file 1: Figure S1. Experimental design of the study. Figure S2. Representative images of CALR immunostaining. Scale bar = 100 μm. Figure S3. Flow cytometry-assisted quantification of surface exposed CALR. Figure S4. Degranulation and IFN-γ production after in vitro stimulation. Figure S5. Prognostic impact of CALR expression in the metastatic TME of HGSC patients and impact of chemotherapy on the final CALR exposure. Figure S6. Transcriptional signatures of the tumor microenvironment of CALRHi versus CALRLo PT and MT samples of HGSCs patients. Figure S7. Chemokine signatures of the tumor microenvironment of CALRHi versus CALRLo of HGSCs patients. Figure S8. Impact of CALR on the frequency of CD8+ T cells and NKp46+ NK cells in MT samples of HGSC patients. Table S1. Main clinical and biological characteristics of 45 HGSC patients after neo-adjuvant chemotherapy treatment (study group 2) (University Hospital Hradec Kralove). Table S2. Main clinical and biological characteristics of 35 HGSC patients without neo-adjuvant chemotherapy treatment prospectively collected (study group 3) (University Hospital Motol). Table S3. The list of antibodies use for IHC staining. Table S4. The list of antibodies used for flow cytometry. Table S5. The list of genes used by MCP counter for identification of distinct cell populations. Table S6. List of genes significantly overrepresented in CALRHi versus CALRLo HGSC samples from TCGA public database. Table S7. List of genes in boxplot significantly overrepresented in CALRHi versus CALRLo HGSC samples from TCGA public database. [file 40425_2019_781_MOESM1_ESM.pdf]

Supplemental Material

Supplemental Material and Methods

Library preparation and sequencing

24 FFPE samples suitable for library preparation according to quality/quantity evaluations were processed following the manufacturer's specifications with minor modifications by using the Illumina TruSeq® RNA Access Library Prep (Illumina) along with purification steps employing SPRI beads. DV200 values for all samples exceeded 40%, and 100 ng of total RNA was used for the cDNA synthesis. Each library was quantified with the fluorimeter Qubit 2.0 (dsDNA HS kit; Thermo Fisher), and the size distribution was determined using a DNA 1000 kit on a 2100 Bioanalyzer instrument prior to pooling. All libraries had a similar size distribution of approximately 260 bp. A 4-plex pool of libraries was made by combining 200 ng of each DNA library. The libraries were sequenced on an Illumina NextSeq 500. At least 180 M pair-end 2x75 bp reads were generated per library. The libraries were prepared and sequenced at EMBL Genomics Core Facility (Heidelberg, Germany).

NGS data analysis. The raw FASTQ sequencing files were aligned to human reference genome (build h19) with bowtie2 (version 2.3.2) and tophat2 (version 2.1). Differentially expressed genes (DEGs) between groups were determined using LIMA-R package with default settings. The MCP-counter R package was used to estimate the abundance of tissue-infiltrating immune cell populations.

Supplemental figures

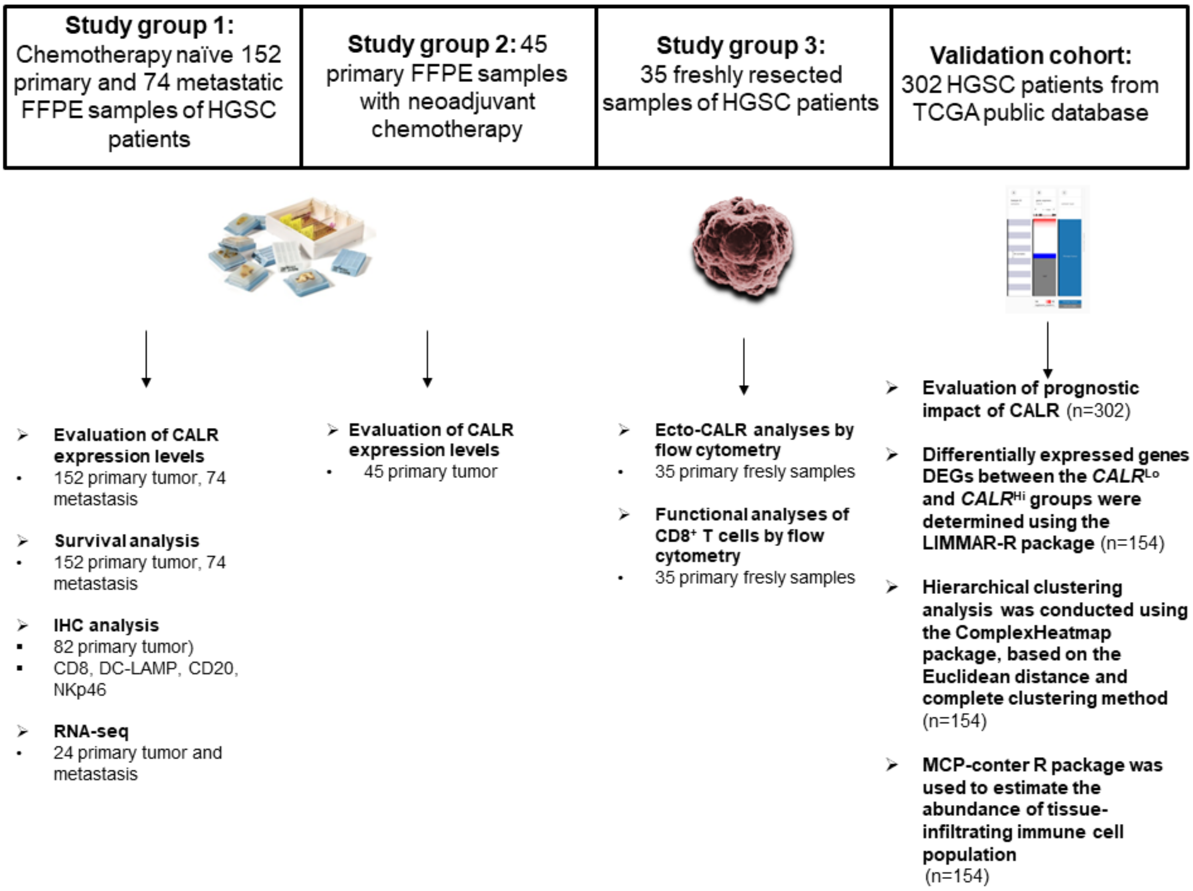

Suppl. Fig. 1. **Experimental design of the study.**

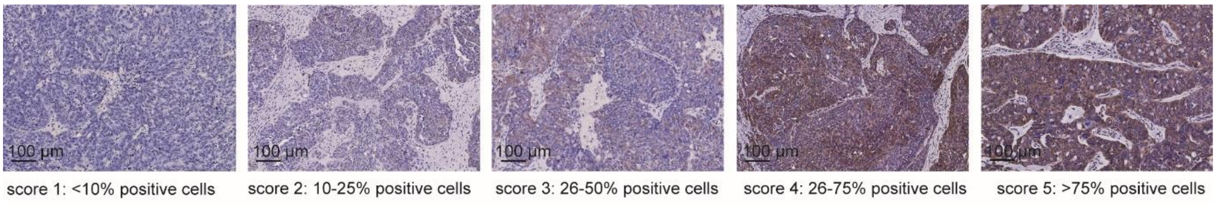

Suppl. Fig. 2 **Representative images of CALR immunostaining. Scale bar = 100 µm.**

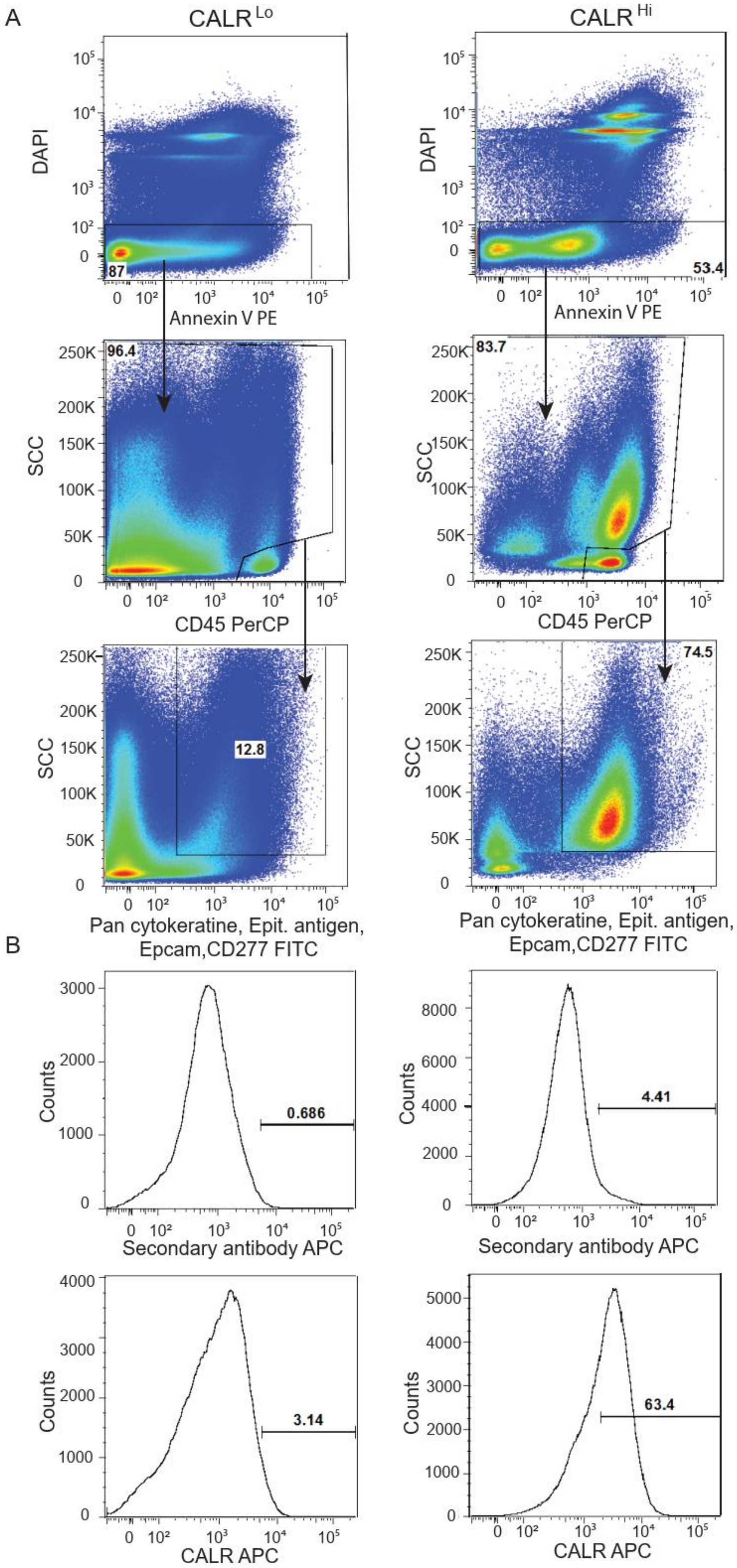
A
CALR Lo
CALR Hi
DAPI
Annexin V PE
87
53.4
SCC
CD45 PerCP
96.4
83.7
12.8
74.5
Pan cytokeratine, Epit. antigen, Epcam,CD277 FITC
B
Counts
Secondary antibody APC
0.686
4.41
CALR APC
3.14
63.4

Suppl. Fig. 3. **Flow cytometry-assisted quantification of surface exposed CALR.**

(A) Gating strategy. The percentage of cells in each gate is reported.

(B) Representative staining of surface exposed CALR for representative $CALR^{Lo}$ (left) and $CALR^{Hi}$ (right) patients.

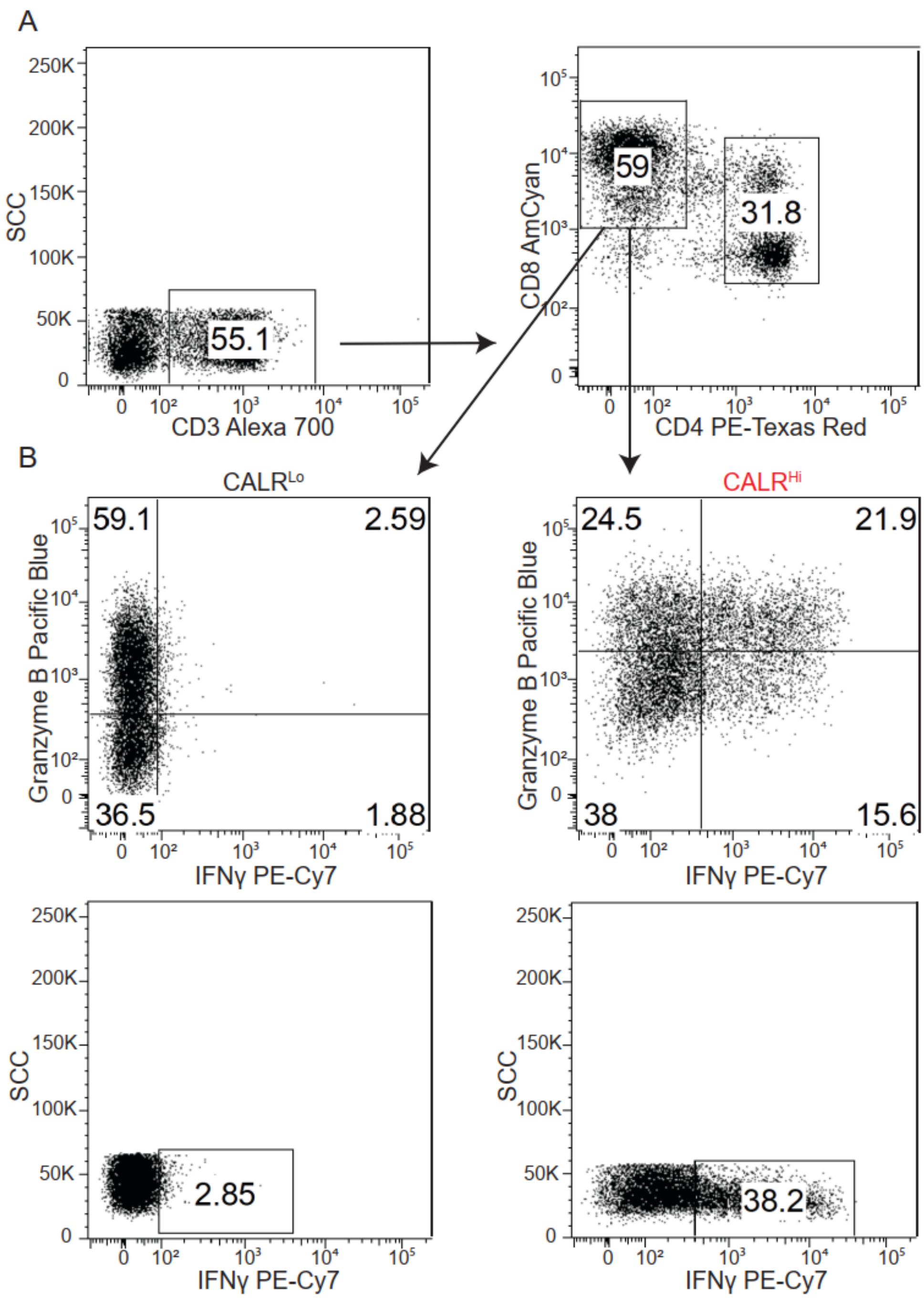

***Suppl. Fig. 4*. Degranulation and IFN-γ production after *in vitro* stimulation**

(A) Gating strategy for CD8$^+$ T cells. The percentage of cells in each gate is reported.

(B) Representative staining of CD8$^+$ T cells degranulatin and IFN-γ production after *in vitro* stimulation for representative CALR^{Lo} (left) and CALR^{Hi} (right) patients.

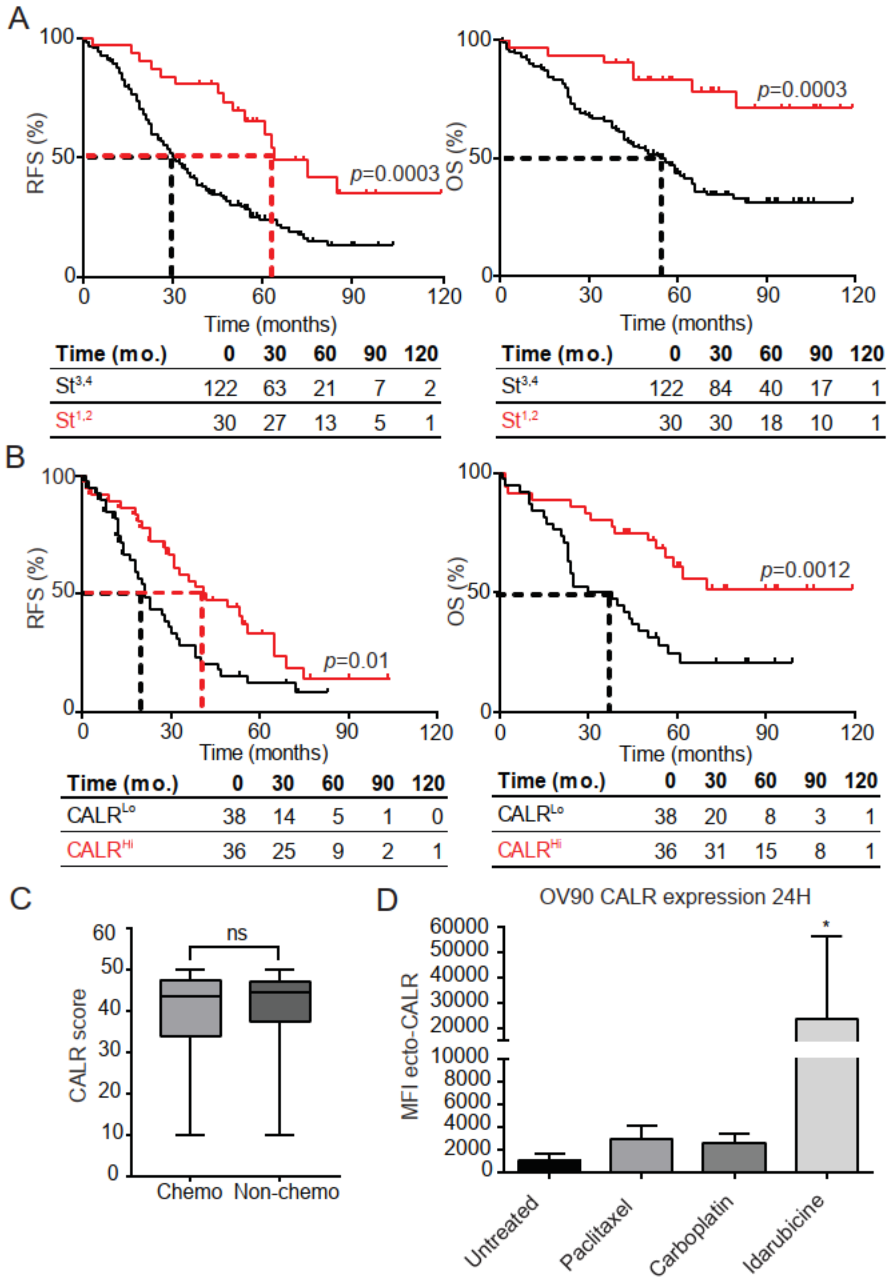

***Suppl. Fig. 5.* Prognostic impact of CALR expression in the metastatic TME of HGSC patients and impact of chemotherapy on the final CALR exposure.**

(A) RFS and OS of 152 HGSC patients from Study Group 1 who did not receive neoadjuvant chemotherapy, upon stratification based on disease stage **(B)** RFS and OS of MT samples from 74 HGSC Study Group 1 patients who did not receive neoadjuvant chemotherapy, upon stratification based on CALR expression. Survival curves were estimated by the Kaplan-Meier

method, and difference between groups were evaluated using log-rank test. Number of patients at risk are reported.

(C) CALR expression levels among 152 chemotherapy-naïve HGSC patients (Study group 1) and 45 HGSC patients receiving neoadjuvant chemotherapy (Study group 2). Box plots: lower quartile, median, upper quartile; whiskers, minimum, maximum.

(D) CALR exposure on OV90 cells left untreated or exposed to 70 μM paclitaxel, 5 mM carboplatin and 20 μM idarubicin for 24 hours. Mean ± SEM (n = 5); **p*<0.05 as compared to untreated cells.

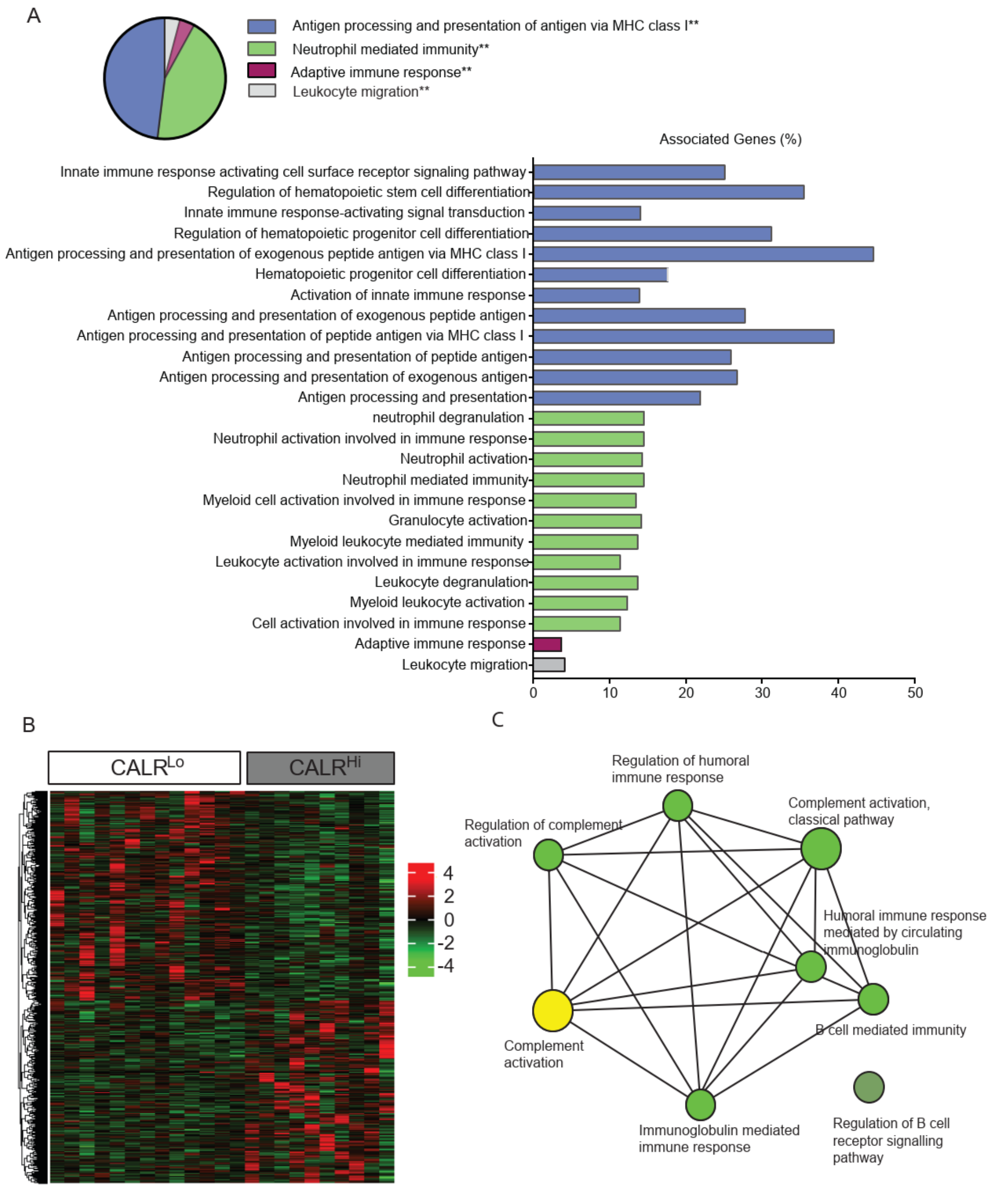

***Suppl. Fig. 6.* Transcriptional signatures of the tumor microenvironment of *CALR*^{Hi} *versus* *CALR*^{Lo} PT and MT samples of HGSCs patients.**

(A) ClueGo analyses of significantly upregulated genes in 77 *CALR*^{Hi} *versus* 77 *CALR*^{Lo} HGSC patients from the TCGA public database (302 patients were divided into 4 groups using quartile stratification, only lower (no=77) and upper (no=77) quartile is presented).

(B) Hierarchical clustering and **(C)** ClueGo analyses of genes significantly upregulated and downregulated in MT samples of 11 CALR^{Hi} *versus* 13 CALR^{Lo} HGSC patients.

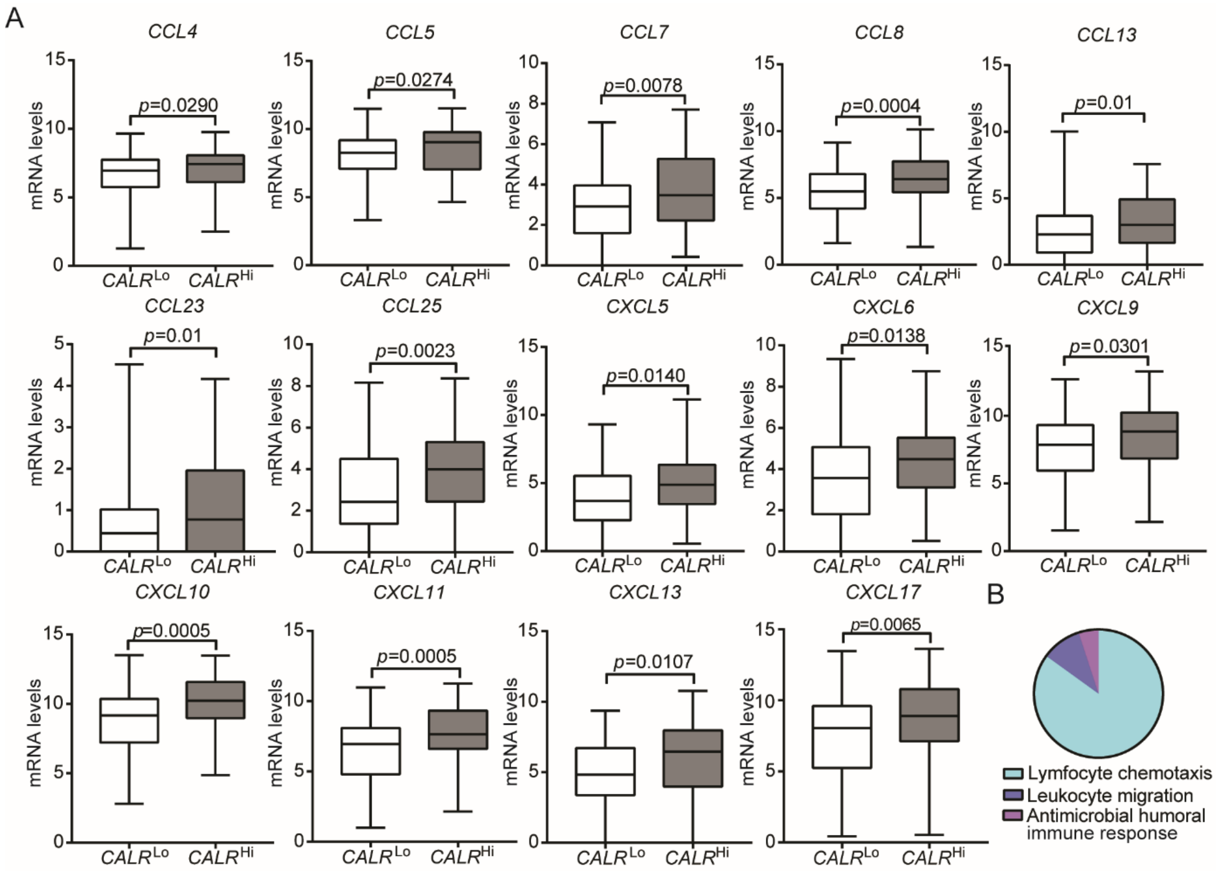

Suppl. Fig. 7. **Chemokine signatures of the tumor microenvironment of *CALR*$^{\mathbf{Hi}}$ *versus* *CALR*$^{\mathbf{Lo}}$ of HGSCs patients.**

(A) Relative expression levels of *CCL4*, *CLL5*, *CCL7*, *CCL8*, *CCL13*, *CCL23*, *CCL25*, *CXCL5*, *CXCL6*, *CXCL9*, *CXCL10*, *CXCL11*, *CXCL13* and *CXCL17* in 77 *CALR*^{Hi} versus 77 *CALR*^{Lo} PT samples from HGSC TCGA patients and **(B)** ClueGo analyses of respective genes (302 patients were divided into 4 groups using quartile stratification, only lower (no=77) and upper (no=77) quartile is presented).

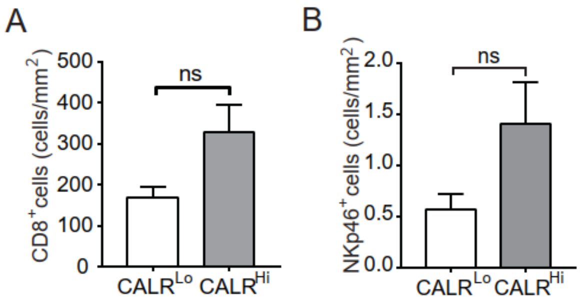

Suppl. Fig. 8. **Impact of CALR on the frequency of $CD8^+$ T cells and $NKp46^+$ NK cells in MT samples of HGSC patients.**

Density of $CD8^+$ T cells **(A)** and $NKp46^+$ cells **(B)** in MT samples of $CALR^{Lo}$ versus $CALR^{Hi}$ HGSC patients (n=74) (Study group 1). Box plots: lower quartile, median, upper quartile; whiskers, minimum, maximum.

Supplemental tables

Supplemental Table 1. **Main clinical and biological characteristics of 45 HGSC patients after neo-adjuvant chemotherapy treatment (study group 2) (University Hospital Hradec Kralove)**

Variable	study group 2 (n=45)
**Age:**	
Mean age (y) ± SEM	65 ± 1.4
Range	33-82
**pTNM stage:**	
Stage I	3 (6.7%)
Stage II	0 (0%)
Stage III and IV	42 (93.3%)
**Debulking**	
R0	22 (48.8%)
R1	3 (6.6%)
R2	20 (44.6%)
**Type of chemotherapy**	
CBDCA+PTX	41 (91.1%)
others	4 (8.9%)
**Vital status of patients**	7 (14,6%)

Supplemental Table 2: **Main clinical and biological characteristics of 35 HGSC patients without neo-adjuvant chemotherapy treatment prospectively collected (study group 3) (University Hospital Motol).**

**Variable**	**Study group 3** (n=35)
**Age:**	
Mean age (y) ± SEM	61 ± 2.484
Range	44-80
**pTNM stage:**	
Stage I	1 (2.9%)
Stage II	6 (17.1%)
Stage III	28 (80%)
**Debulking**	
R0	17 (48.5%)
R1	7 (20%)
R2	11 (31,5%)

Supplemental Table 3: **The list of antibodies use for IHC staining.**

Parameter	Source	Producer	Clone	Detection system	Revelation	Dilution	Incubation time [min]
**CD20***	mouse	Dako	L26	ImmPRESS-AP anti-mouse IgG (alkaline phosphatase) Polymer Detection Kit	ImmPACT Vector red Alkaline Phosphatase substrate kit	1:250	60
**CD8**	rabbit	Spring Bioscience	SP16	EnVision™+/HRP, Rabbit	DAB+ substrate Chromogen system	1:80	30
**DC-LAMP***	rat	Dendritics	1010E1.01	donkey anti-rat IgG-biot (Jackson ImmunoResearch)	DAB+ substrate Chromogen system	1:80	60
**NKp46**	mouse	RD systems	195314	Impress HRP anti-mouse IgG (Peroxidase) Polymer Detection kit	DAB+ substrate Chromogen system	1:100	90
**CALR**	mouse	Abcam	FMC75	donkey anti-mouse IgG-biot (Jackson ImmunoResearch)	DAB+ substrate Chromogen system	1:200	120

***CD20 and DC-LAMP were stained in double staining protokol**

***Supplemental Table 4.* The list of antibodies used for flow cytometry.**

Assay	Parameter	Source	Producer	Clone	Fluorochrome	Dilution
Exposure of CALR	**Calreticulin**	mouse	Enzo Life Sciences, Inc.	FMC 75	-	2.4:100
	**CD227**	mouse	BD Biosciences	HMPV	FITC	2:100
	**Epitelial antigen**	mouse	DAKO	Ber-EP4	FITC	2:100
	**Epcam**	mouse	BioLegend	9C4	FITC	2:100
	**Pan cytokeratin**	murine	eBioscience	AE1/AE3	A488	2:100
	**CD45**	mouse	EXBIO	MEM-28	PerCP	6:100
Stimaltion assay	**CD3**	mouse	EXBIO	MEM-57	Alexa 700	5:100
	**CD4**	mouse	Beckman Coulter	SFCI12T4D11	ECD	5:100
	**CD8**	mouse	BD Biosciences	RPA-T8	HV500	5:100
	**Granzyme**	mouse	BD Biosciences	GB11	BV421	4:100
	**IFNg**	murine	eBioscience	4S.B3	PE-Cy7	1:100

***Supplemental Table 5*. The list of genes used by MCP counter for identification of distinct cell populations.**

T cells	CD8 T cells	Cytotoxic lymphocytes	B lineage	NK cells	Monocytic lineage	Myeloid dendritic cells	Neutrophils	Endothelial cells		Fibroblasts
CD28	CD8B	CD8A	BANK1	CD160	ADAP2	CD1A	CA4	ACVRL1	KDR	COL1A1
CD3D		EOMES	CD19	KIR2DL1	CSF1R	CD1B	CEACAM3	APLN	MMRN1	COL3A1
CD3G		FGFBP2	CD22	KIR2DL3	FPR3	CD1E	CXCR1	BCL6B	MMRN2	COL6A1
CD5		GNLY	CD79A	KIR2DL4	KYNU	CLEC10A	CXCR2	BMP6	MYCT1	COL6A2
CD6		KLRC3	CR2	KIR3DL1	PLA2G7	CLIC2	CYP4F3	BMX	PALMD	DCN
CHRM3-A5		KLRC4	FCRL2	KIR3DS1	RASSF4	WFDC21P	FCGR3B	CDH5	PEAR1	GREM1
CTLA-4		KLRD1	IGKC	NCR1	TFEC		HAL	CLEC14A	PGF	PAMR1
FLT3LG			MS4A1	PTGDR			KCNJ15	CXorf36	PLXNA2	TAGLN
ICOS			PAX5	SH2D1B			MEGF9	EDN1	PTPRB	
MAL							SLC25A37	ELTD1	ROBO4	
MGC40069							STEAP4	EMCN	SDPR	
PBX4							TECPR2	ESAM	SHANK3	
SIRPG							TLE3	ESM1	SHE	
THEMIS							TNFRSF10C	FAM124B	TEK	
TNFRSF25							VNN3	HECW2	TIE1	
TRAT1								HHIP	VEPH1	
									VWF	

Supplemental Table 6. **List of genes significantly overrepresented in *CALR*^{Hi} versus *CALR*^{Lo} HGSC samples from TCGA public database.**

Gene symbol	Base mean	log2(FC)	StdErr	Wald-Stats	P-value	P-adj
MUC5B	-0.916925721	-1.184547727	7.725090422	-3.363400091	8.67E-04	1.18E-02
TMEM100	-2.453900898	-0.846440909	6.391571104	-2.835727139	4.88E-03	3.88E-02
KCNJ4	0.52803596	-0.830827597	2.861216721	-3.797335501	1.76E-04	3.93E-03
LOC648740	4.510801398	-0.828086039	3.091973214	-4.811220771	2.36E-06	2.05E-04
FAM135B	-1.402105572	-0.817215584	3.415451948	-3.205633858	1.49E-03	1.73E-02
CSMD2	-0.458468524	-0.814016234	5.396208117	-3.506417793	5.22E-04	8.36E-03
KCNQ5	-2.523058163	-0.78387987	4.197041883	-2.80980099	5.28E-03	4.12E-02
LOC283174	-1.193999777	-0.768810065	4.961617045	-3.274169592	1.18E-03	1.45E-02
AMY2B	2.805255338	-0.766977922	6.182608442	-4.402337171	1.48E-05	7.23E-04
ZFPM2	3.039215194	-0.763869156	7.523738799	-4.460348512	1.15E-05	6.10E-04
SLC9A3	2.766369192	-0.752196429	2.834474513	-4.392630165	1.54E-05	7.44E-04
C9orf45	8.00396042	-0.732597403	6.93995974	-5.568194282	5.62E-08	1.57E-05
NTNG1	-2.724220719	-0.724520455	4.414437175	-2.733063028	6.64E-03	4.80E-02
EPPK1	2.975981811	-0.723984416	10.36706104	-4.444734904	1.23E-05	6.36E-04
KIRREL3	-2.203563102	-0.714372727	3.47347013	-2.927756797	3.67E-03	3.21E-02
GOLGA8B	1.644510577	-0.71087987	8.218295779	-4.10391617	5.21E-05	1.65E-03
C12orf53	-2.666839808	-0.707371429	5.771088961	-2.755159164	6.22E-03	4.60E-02
CDH4	-0.729037861	-0.705938312	1.763449026	-3.422680013	7.04E-04	1.02E-02
CCND2	-0.227916871	-0.696417857	9.406448864	-3.576344199	4.05E-04	7.00E-03
LOC100272216	2.641191855	-0.695275325	3.556484416	-4.361253873	1.77E-05	8.26E-04
FCGBP	-0.542121053	-0.694297403	9.647622078	-3.480726803	5.72E-04	8.94E-03
KIAA1644	-1.466814679	-0.684629221	4.926795779	-3.184043007	1.60E-03	1.80E-02
PAK3	-0.537874896	-0.678852273	2.319399838	-3.482035069	5.70E-04	8.92E-03
CYP2D7P1	4.811525569	-0.674593182	3.241854058	-4.880240517	1.71E-06	1.68E-04
ARHGAP20	0.660230054	-0.667985065	5.987136039	-3.834787194	1.52E-04	3.57E-03
LOC148696	4.885647149	-0.667625974	2.371407143	-4.89712485	1.57E-06	1.58E-04
ZMAT1	2.07602386	-0.663570779	6.356092532	-4.217038776	3.26E-05	1.23E-03
FLJ42627	6.756809559	-0.658847403	5.747931494	-5.308264138	2.13E-07	4.01E-05
LOC339535	-2.27640824	-0.657570455	7.896224513	-2.901262091	3.99E-03	3.40E-02
MGAT3	1.49052307	-0.654521429	9.055044481	-4.062869462	6.16E-05	1.85E-03
DRD2	-1.940140334	-0.637869156	4.776824513	-3.021736563	2.72E-03	2.61E-02
SATB1	2.02964828	-0.635819805	8.067835552	-4.205011106	3.43E-05	1.27E-03
PRKCA	6.203566732	-0.633904221	8.994610877	-5.189546422	3.84E-07	5.91E-05
C9orf131	3.20833456	-0.63348474	3.053971916	-4.501874845	9.57E-06	5.37E-04
STRC	-0.641921229	-0.632463636	4.099272727	-3.44984612	6.39E-04	9.57E-03
SSPO	-1.859027273	-0.628676623	5.432098052	-3.050125514	2.49E-03	2.45E-02
PNPLA7	1.686432673	-0.62417987	6.21723539	-4.11502752	4.98E-05	1.61E-03
LOC400931	2.956925262	-0.620796104	6.512424026	-4.440020039	1.26E-05	6.43E-04
POLN	3.287385388	-0.613240584	4.233664123	-4.521171155	8.79E-06	5.11E-04

PLCH2	-2.411817223	-0.593655519	4.810624188	-2.85139442	4.65E-03	3.76E-02
XIST	-2.709507123	-0.592982468	10.82516006	-2.738745067	6.53E-03	4.75E-02
RUNDC2C	2.919846119	-0.592636364	3.892399351	-4.430833542	1.31E-05	6.60E-04
LONRF2	-1.364987251	-0.59241461	9.141665422	-3.217957731	1.43E-03	1.68E-02
CAPN3	1.226931363	-0.591836688	5.690640097	-3.991731538	8.21E-05	2.27E-03
BTBD19	0.837622334	-0.590401299	5.05302013	-3.884529731	1.26E-04	3.08E-03
FMN2	-1.867642563	-0.590068182	2.880453571	-3.047122026	2.51E-03	2.47E-02
LOC646851	4.289947685	-0.58950974	6.649108766	-4.759988521	2.99E-06	2.45E-04
LRRC4C	-2.279103698	-0.587370455	5.40336737	-2.900277333	4.00E-03	3.40E-02
MYO15B	1.637930102	-0.586969481	9.080215909	-4.102169593	5.25E-05	1.65E-03
RFX2	2.557039045	-0.586172403	7.494578084	-4.340048555	1.94E-05	8.76E-04
STON1-GTF2A1L	0.8278097	-0.583101623	0.69242711	-3.881793233	1.27E-04	3.10E-03
KCND3	-2.603518767	-0.580956818	3.587915097	-2.779348923	5.78E-03	4.36E-02
WDR52	2.43813754	-0.580258117	6.611594968	-4.309930567	2.20E-05	9.33E-04
CG030	4.794297452	-0.579816234	4.582452922	-4.87630896	1.74E-06	1.69E-04
ZFHX4	-2.359653865	-0.578788312	6.09782013	-2.87070187	4.38E-03	3.62E-02
FLJ13197	2.862166939	-0.577136039	5.164538474	-4.416510058	1.39E-05	6.93E-04
MYH3	0.103047341	-0.576362987	4.044752273	-3.674589116	2.81E-04	5.52E-03
LOC100190986	6.802557002	-0.574910714	7.767853084	-5.317982471	2.02E-07	3.88E-05
LOC221442	-1.175390781	-0.573303247	4.418719156	-3.280233134	1.16E-03	1.43E-02
CROCCL2	5.542859213	-0.571719481	5.858358442	-5.044731423	7.78E-07	9.40E-05
B3GALT2	2.005006627	-0.56985487	3.403395292	-4.198607772	3.52E-05	1.29E-03
PRICKLE1	-0.197408036	-0.566693506	8.208207143	-3.585503662	3.91E-04	6.85E-03
RUNX1T1	-2.647287931	-0.563312662	4.274940422	-2.762649793	6.08E-03	4.53E-02
GOLGA6L5	2.174279546	-0.561692532	4.126216721	-4.242421805	2.93E-05	1.15E-03
CRIPAK	6.085875538	-0.561162987	7.214400325	-5.163999555	4.35E-07	6.38E-05
NPIP	-1.165655934	-0.557741883	7.217245942	-3.283400981	1.14E-03	1.42E-02
TAS2R4	4.794687177	-0.557138312	1.67795487	-4.876397928	1.74E-06	1.69E-04
SPTBN5	-0.824065558	-0.554888636	5.473932955	-3.392817898	7.82E-04	1.10E-02
C15orf52	1.300211657	-0.554750974	7.954045292	-4.011621835	7.58E-05	2.16E-03
LOC641298	2.278069603	-0.554333442	6.928829383	-4.269089329	2.62E-05	1.07E-03
SFMBT2	-0.472501401	-0.553848052	4.856250649	-3.502120195	5.30E-04	8.46E-03
MAPK15	-0.909998058	-0.550743831	8.80962224	-3.365602971	8.61E-04	1.18E-02
CHAD	-0.455091014	-0.550698052	3.870500974	-3.507451439	5.20E-04	8.36E-03
KCNT2	-2.621554616	-0.548853247	4.527118831	-2.772479324	5.90E-03	4.43E-02
SYT6	1.001867941	-0.548482792	2.518676136	-3.930078265	1.05E-04	2.72E-03
LOC92973	1.412069621	-0.548459091	5.021338636	-4.041813796	6.71E-05	1.97E-03
SEC31B	0.171691949	-0.546916883	6.014824026	-3.694666574	2.61E-04	5.19E-03
LOC100132247	6.946678643	-0.546132792	9.319731656	-5.348503565	1.74E-07	3.49E-05
NBEA	-1.788667585	-0.544421429	5.582341234	-3.074551357	2.30E-03	2.32E-02
RPL23AP53	9.297277279	-0.543428247	6.138217045	-5.827748315	1.42E-08	5.13E-06
CEACAM19	0.372871742	-0.542902922	6.327054383	-3.752944028	2.09E-04	4.43E-03
SGK223	6.514751252	-0.539402597	9.13608961	-5.256595681	2.75E-07	4.69E-05
PLEKHH2	-0.060127912	-0.538507143	6.827070455	-3.626455846	3.36E-04	6.21E-03
ZNF154	5.164075809	-0.537621429	5.090578896	-4.960112168	1.17E-06	1.28E-04

TXK	-1.184654061	-0.537303896	5.204924026	-3.277216098	1.17E-03	1.44E-02
SYT12	-2.194073823	-0.536354221	7.308411851	-2.93119152	3.63E-03	3.18E-02
MASP2	2.637277644	-0.534891234	4.367335552	-4.360269559	1.77E-05	8.28E-04
C1QTNF7	-1.933660202	-0.533361688	5.389399026	-3.024013765	2.70E-03	2.60E-02
ZBTB20	6.501425436	-0.532543506	6.160160065	-5.253739012	2.79E-07	4.69E-05
NTN5	-1.608337919	-0.531237338	3.988291721	-3.1363388	1.88E-03	2.00E-02
SGSM2	13.72313581	-0.531057143	9.739434416	-6.65501865	1.30E-10	1.03E-07
LOC90834	2.621871636	-0.528640584	3.61165763	-4.356393482	1.80E-05	8.35E-04
RPL21P44	4.611570846	-0.52707987	2.390080195	-4.834442183	2.11E-06	1.89E-04
KSR2	-0.360478579	-0.524266234	6.591367532	-3.536293431	4.68E-04	7.77E-03
PCLO	-2.186420508	-0.522714286	6.52689026	-2.933958936	3.60E-03	3.17E-02
ATP8A1	2.348160125	-0.522459091	7.242363312	-4.287015168	2.43E-05	1.00E-03
MGC16384	4.287926921	-0.520985065	2.785467208	-4.759517577	2.99E-06	2.45E-04
PLEC	3.185592162	-0.520288636	13.9020336	-4.496310058	9.81E-06	5.45E-04
MYO5C	7.206874331	-0.520146429	9.453514448	-5.403246157	1.31E-07	2.90E-05
KCNIP4	-0.752258004	-0.518113636	4.923599026	-3.415405455	7.23E-04	1.04E-02
SYNE2	8.017287957	-0.517678896	10.56488653	-5.570918437	5.54E-08	1.57E-05
LOC100129034	9.710177329	-0.515015584	10.64364675	-5.908688816	9.15E-09	3.54E-06
DOK6	-0.303584337	-0.514842857	4.088526623	-3.553533616	4.40E-04	7.46E-03
RASSF8	-1.567143261	-0.514323701	8.249686851	-3.150294246	1.79E-03	1.94E-02
FLRT2	-2.25639566	-0.510082468	7.525293182	-2.908563629	3.90E-03	3.34E-02
ZNF469	-1.994479498	-0.509838312	8.559799675	-3.002577177	2.90E-03	2.72E-02
LOC100272228	0.043385125	-0.509442532	5.536327435	-3.657057168	3.00E-04	5.73E-03
AMT	-1.612287531	-0.508429545	6.802194968	-3.134997743	1.88E-03	2.01E-02
C3orf65	-0.730282418	-0.508322078	2.594095455	-3.422290472	7.05E-04	1.02E-02
KLRA1	2.187434705	-0.506964286	4.917356818	-4.245810055	2.89E-05	1.14E-03
AKAP12	-1.651339927	-0.505997403	9.898322727	-3.121708817	1.97E-03	2.07E-02
JRK	7.149816044	-0.505613636	8.970044481	-5.391280763	1.40E-07	3.02E-05
FLJ45340	5.193238365	-0.504760065	9.741621916	-4.966670247	1.13E-06	1.26E-04
GOLGA6L10	3.22341606	-0.504507143	6.537855519	-4.505561798	9.42E-06	5.34E-04
EMR4P	-1.16272976	-0.503472078	1.766786039	-3.284352641	1.14E-03	1.42E-02
SERTAD4	-2.316747937	-0.503198701	6.306990909	-2.886491224	4.17E-03	3.51E-02
FLJ10661	0.616108356	-0.500871104	4.411039123	-3.822324127	1.60E-04	3.68E-03
AGBL2	-1.756657952	-0.500647727	4.636685877	-3.085603647	2.22E-03	2.25E-02
CX3CR1	-2.441031623	-0.500600649	7.430547727	-2.840526912	4.80E-03	3.85E-02
C9orf117	-1.714721918	-0.496819805	5.564375812	-3.100027393	2.11E-03	2.18E-02
GALNTL2	-1.843708715	-0.496090584	4.868759253	-3.055459065	2.44E-03	2.43E-02
GOLGA2B	0.940542512	-0.495951948	7.107287013	-3.913127466	1.12E-04	2.86E-03
CATSPER2	1.075638785	-0.495552597	4.774947078	-3.950382205	9.68E-05	2.56E-03
PRRT2	-0.756905827	-0.492958442	6.178415584	-3.413947633	7.26E-04	1.05E-02
NR2F1	-1.345107909	-0.492395455	7.842262013	-3.224539922	1.40E-03	1.65E-02
SBF1P1	-0.178482401	-0.491639935	1.648871591	-3.591174811	3.83E-04	6.75E-03
CUBN	-0.806931597	-0.491116234	4.690952273	-3.398220115	7.68E-04	1.09E-02
RYR3	-1.773617426	-0.490433766	3.61798961	-3.079752512	2.26E-03	2.29E-02
SPINLW1	-2.557161061	-0.489570455	3.027392695	-2.796932413	5.48E-03	4.23E-02

CHRNA6	-2.240756446	-0.488190584	2.609485227	-2.914257525	3.83E-03	3.30E-02
NLRP1	-0.821713164	-0.487520779	6.515767532	-3.393560063	7.80E-04	1.10E-02
TSSK4	3.411386991	-0.485670455	3.976828409	-4.551296344	7.69E-06	4.67E-04
ALMS1P	0.766748071	-0.485253247	1.977022727	-3.864725341	1.36E-04	3.25E-03
PRINS	1.864887444	-0.482242532	5.567615097	-4.162030442	4.10E-05	1.45E-03
LOC619207	-2.360306247	-0.481521429	4.607451623	-2.87046116	4.38E-03	3.62E-02
TSHZ2	-1.338790063	-0.481018506	8.160901136	-3.226629184	1.39E-03	1.64E-02
CLN8	8.303235862	-0.480501623	7.60614789	-5.62910916	4.09E-08	1.23E-05
NPIPL3	3.913124247	-0.480014286	10.09466429	-4.671464477	4.48E-06	3.24E-04
FAM22F	3.051761202	-0.479765584	2.335070455	-4.463440698	1.13E-05	6.05E-04
PKD1	6.300916343	-0.478832143	10.44904562	-5.210598891	3.46E-07	5.46E-05
VENTX	-0.893926791	-0.478181818	3.554672078	-3.370708227	8.46E-04	1.16E-02
BZRAP1	2.484508164	-0.476322727	8.994993831	-4.321698339	2.09E-05	9.07E-04
FAM22G	2.45417322	-0.476268831	3.000996104	-4.314003246	2.16E-05	9.25E-04
RNF150	1.148770525	-0.476241883	8.447081006	-3.970417965	8.94E-05	2.42E-03
KCTD12	1.336861056	-0.475458766	11.85930114	-4.021536375	7.28E-05	2.09E-03
PLA2G6	3.3791694	-0.475458442	8.683564935	-4.54348605	7.96E-06	4.78E-04
BMS1P5	6.503592561	-0.474542532	6.033720942	-5.254203668	2.79E-07	4.69E-05
FLJ42393	1.028476871	-0.474494156	3.467156818	-3.937412708	1.02E-04	2.66E-03
NBPF10	2.530625096	-0.473205195	8.332013636	-4.333373786	1.99E-05	8.87E-04
ANKRD24	-1.071645937	-0.471754545	3.705547403	-3.313848157	1.03E-03	1.33E-02
ABCA10	-1.595853228	-0.47164026	2.575564286	-3.140574345	1.85E-03	1.99E-02
C6orf59	-0.979902172	-0.471086364	3.400470455	-3.343312961	9.30E-04	1.24E-02
ADAM28	-2.003303851	-0.471057792	7.707672403	-2.999454942	2.93E-03	2.74E-02
GNRH1	-0.106491575	-0.469774351	3.749366071	-3.612672606	3.54E-04	6.40E-03
PRICKLE4	-0.852197769	-0.467915909	5.966044643	-3.383930704	8.07E-04	1.13E-02
AHNAK	3.422173085	-0.467097403	13.73980714	-4.553908542	7.60E-06	4.63E-04
NLRC3	3.387094627	-0.466401299	6.072645455	-4.545408392	7.89E-06	4.77E-04
FSCN2	-0.545054811	-0.46532987	4.503585065	-3.47982263	5.74E-04	8.94E-03
C8orf42	-0.965833971	-0.465213636	7.341527597	-3.347809881	9.16E-04	1.22E-02
RPL23AP64	3.088135612	-0.464002597	2.391451299	-4.472395254	1.09E-05	5.87E-04
GOLGA8A	-1.5699195	-0.462975974	9.201942532	-3.149355562	1.80E-03	1.95E-02
NCRNA00201	0.063984673	-0.460746429	9.309106656	-3.663119078	2.93E-04	5.65E-03
DNAH7	-2.7682684	-0.460728896	5.377992045	-2.7159853	6.98E-03	4.98E-02
DYNC2H1	2.011069034	-0.460653896	7.512213961	-4.200183942	3.50E-05	1.29E-03
KIAA1875	-2.466403485	-0.46007987	5.819428247	-2.831056746	4.95E-03	3.92E-02
SNED1	-0.230671641	-0.458601299	8.591557143	-3.575516094	4.06E-04	7.01E-03
ANKK1	-1.982023552	-0.458278247	2.744210227	-3.00697915	2.86E-03	2.69E-02
BEAN	-1.751640461	-0.45773474	4.476889773	-3.087332722	2.20E-03	2.24E-02
MALAT1	0.27671273	-0.457039286	14.33638425	-3.725192413	2.32E-04	4.77E-03
GPRASP1	1.00168641	-0.456801948	5.928421753	-3.930028186	1.05E-04	2.72E-03
FAM154B	-2.761822075	-0.456711364	4.803903409	-2.718490979	6.93E-03	4.96E-02
SLC25A27	-1.06031073	-0.456703571	6.692808604	-3.317501768	1.02E-03	1.32E-02
ARHGEF10	1.678821242	-0.456636364	8.655308442	-4.113012118	5.02E-05	1.61E-03
TNFRSF10D	-1.386606128	-0.456163312	6.198984578	-3.210785285	1.46E-03	1.71E-02

SPDYE6	0.348519525	-0.456012662	5.59103263	-3.745933637	2.15E-04	4.50E-03
LOC100131434	-1.232727262	-0.455689286	5.897721591	-3.261517037	1.23E-03	1.50E-02
CCDC154	-2.632386424	-0.455543182	3.475015422	-2.768345864	5.98E-03	4.48E-02
EFCAB6	-0.471231886	-0.454528571	5.214705195	-3.502509186	5.29E-04	8.46E-03
C16orf3	1.136306263	-0.452353571	1.945600487	-3.96700959	9.06E-05	2.44E-03
PHLDB2	-2.328095003	-0.452162987	7.347069805	-2.882323467	4.23E-03	3.54E-02
LOC100128842	-1.031364773	-0.450923052	5.059394968	-3.326814843	9.85E-04	1.29E-02
C3orf35	1.937377313	-0.449654545	3.121292857	-4.180989019	3.79E-05	1.37E-03
LOC286367	2.155582651	-0.44855974	5.17159026	-4.237602094	2.99E-05	1.16E-03
FBXO41	1.597382033	-0.448224675	8.703892857	-4.091392718	5.48E-05	1.71E-03
LOC283922	1.76563336	-0.447269156	6.234674513	-4.135946788	4.57E-05	1.55E-03
KCTD19	-2.58621711	-0.44486461	2.647595617	-2.785923764	5.67E-03	4.31E-02
P2RX6	-2.223201652	-0.444753247	2.365419481	-2.920636353	3.75E-03	3.25E-02
KIAA1407	2.52410438	-0.441571753	7.514672565	-4.331724616	2.01E-05	8.87E-04
BCO2	-1.615447568	-0.441419156	4.225209903	-3.133924393	1.89E-03	2.01E-02
MMP16	-2.354844513	-0.441325325	3.434202273	-2.872475793	4.36E-03	3.61E-02
TUBGCP6	7.892844586	-0.441321753	9.019867695	-5.545439958	6.33E-08	1.68E-05
TLN2	2.34002931	-0.440930195	9.130925812	-4.284939088	2.45E-05	1.01E-03
CACNB2	2.068250546	-0.440418831	4.163225649	-4.215024864	3.29E-05	1.23E-03
C9orf84	1.353814441	-0.439893831	2.014915422	-4.026115265	7.15E-05	2.07E-03
KIAA1529	-1.480454317	-0.43988539	6.488004058	-3.179474538	1.63E-03	1.82E-02
ACRC	-1.653216741	-0.439571104	4.23969237	-3.121068832	1.97E-03	2.07E-02
EFHA2	-1.257054134	-0.439378247	5.399319318	-3.253545855	1.27E-03	1.52E-02
TSSK3	-0.224439001	-0.438533117	4.379152273	-3.577389423	4.03E-04	6.99E-03
AHSA2	1.669927933	-0.438423052	8.518528084	-4.110656177	5.07E-05	1.61E-03
ABCA2	-0.363733981	-0.437248377	10.29530828	-3.535304638	4.70E-04	7.79E-03
WDR19	6.554999878	-0.435828896	8.649210877	-5.265216018	2.64E-07	4.67E-05
SPDYE5	0.489853078	-0.435735714	4.057984091	-3.786455476	1.84E-04	4.04E-03
NBPF9	5.734823811	-0.435193831	7.001000487	-5.087162341	6.34E-07	8.18E-05
WDR27	1.369979996	-0.435098052	7.168269805	-4.030477029	7.02E-05	2.05E-03
KIAA1530	3.316971844	-0.434563636	7.311133766	-4.528374784	8.52E-06	4.99E-04
LUZP2	-1.844649198	-0.434287013	1.920082468	-3.055131864	2.45E-03	2.43E-02
LOC339047	-0.827928702	-0.43419513	8.760143344	-3.391598776	7.86E-04	1.11E-02
TAS2R5	-1.950644207	-0.434119805	3.573628409	-3.018041934	2.76E-03	2.63E-02
BAHCC1	-2.73209979	-0.433775649	6.722968019	-2.730015696	6.70E-03	4.83E-02
ADAMTS10	-2.621475464	-0.433681494	6.526609903	-2.772509507	5.90E-03	4.43E-02
BNC2	-2.271283186	-0.43255	8.456563312	-2.903133606	3.96E-03	3.38E-02
SLC35E2	-1.069310171	-0.432367857	6.377749188	-3.314601337	1.03E-03	1.33E-02
ARGFXP2	3.338051005	-0.432181169	1.809913312	-4.533501001	8.32E-06	4.92E-04
FDPSL2A	0.57229418	-0.432073377	3.092963312	-3.809911382	1.68E-04	3.80E-03
ZNF462	-2.347019791	-0.43150487	8.524698214	-2.875359719	4.32E-03	3.59E-02
VN1R1	-1.239931391	-0.429230195	4.304576461	-3.259158353	1.24E-03	1.50E-02
GHRL	-1.374480212	-0.428862013	2.633907955	-3.214810111	1.44E-03	1.69E-02
TANC2	2.278958409	-0.42872013	9.641576948	-4.26931706	2.62E-05	1.07E-03
ZNF514	7.354729905	-0.428405519	7.445886201	-5.434151591	1.12E-07	2.51E-05

IGF1R	-1.094703337	-0.428305519	10.93783166	-3.306404633	1.06E-03	1.35E-02
DNHD1	-1.304983765	-0.427277273	7.634228247	-3.237787305	1.34E-03	1.59E-02
SMCR5	0.665386698	-0.425041234	1.523341071	-3.836241399	1.52E-04	3.55E-03
LOC100131551	-2.147213109	-0.424844156	2.380866883	-2.948097765	3.44E-03	3.08E-02
PIWIL2	-1.325243776	-0.424545455	1.739212987	-3.231104589	1.37E-03	1.62E-02
C6orf163	0.863285804	-0.424212987	2.926315584	-3.891678415	1.22E-04	3.04E-03
KIAA1671	0.728313773	-0.423943506	10.77510422	-3.853947193	1.42E-04	3.35E-03
HEG1	-0.840166325	-0.423420779	10.31609545	-3.387734176	7.97E-04	1.12E-02
NAIP	0.853738734	-0.423309091	5.66906039	-3.889020419	1.23E-04	3.06E-03
MSRB3	-1.316585989	-0.419763636	8.023159091	-3.233961914	1.35E-03	1.61E-02
WFDC8	-2.53572451	-0.419570455	1.382140747	-2.805027936	5.35E-03	4.16E-02
EME2	-0.25845895	-0.419187987	4.530598864	-3.56715315	4.18E-04	7.18E-03
C15orf28	-0.854254348	-0.418977922	3.610845455	-3.383280167	8.09E-04	1.13E-02
RPS15AP10	0.319810456	-0.417341558	3.274754545	-3.737653658	2.21E-04	4.61E-03
SPATA13	1.034686019	-0.416510065	8.059393669	-3.939122417	1.01E-04	2.65E-03
LOC283314	-0.721642849	-0.416063312	6.54465211	-3.424993773	6.98E-04	1.02E-02
NKTR	5.899956394	-0.415807792	9.821490909	-5.123426214	5.31E-07	7.32E-05
LOC728264	-2.151394731	-0.415444481	6.433471266	-2.946592852	3.46E-03	3.09E-02
SIGLEC16	-2.342025896	-0.415098701	3.499324675	-2.877198869	4.29E-03	3.58E-02
C22orf36	-2.658866359	-0.414816558	7.63485276	-2.75821625	6.16E-03	4.57E-02
PLGLB2	0.637532615	-0.414673701	5.098267045	-3.828380433	1.56E-04	3.62E-03
TTLL3	0.934382581	-0.414355844	9.082640909	-3.911421156	1.13E-04	2.87E-03
SRRM2	7.429998608	-0.413601948	13.85525682	-5.449829446	1.04E-07	2.36E-05
SYNGR1	-2.736448054	-0.411888961	8.150404221	-2.728332563	6.73E-03	4.85E-02
ADAMTS13	-1.79353673	-0.411058766	6.640051786	-3.072866871	2.31E-03	2.32E-02
MLLT6	6.500198646	-0.410688961	11.85393279	-5.253475959	2.80E-07	4.69E-05
C8orf77	-2.204323057	-0.40879026	5.471346429	-2.927481561	3.67E-03	3.21E-02
ITGA10	-2.601315761	-0.40841526	4.566611851	-2.780186912	5.77E-03	4.35E-02
SNORD1C	-1.00586574	-0.408254545	4.999337013	-3.334998904	9.58E-04	1.26E-02
LOC100271836	4.416371576	-0.407759091	8.230928896	-4.789372969	2.61E-06	2.18E-04
SPDYE1	0.883943224	-0.407116558	3.895727435	-3.897424039	1.19E-04	2.99E-03
DAD1L	-0.937583435	-0.40587987	0.915119156	-3.356823333	8.88E-04	1.20E-02
TTBK2	3.889827592	-0.405265909	5.817873539	-4.665944158	4.59E-06	3.26E-04
WDR90	2.585777756	-0.404457143	9.30667013	-4.347300536	1.88E-05	8.58E-04
PI4KAP1	-0.541104741	-0.402992532	7.722889448	-3.481039976	5.72E-04	8.93E-03
NCRNA00107	-0.531220015	-0.402957143	3.091366883	-3.484084567	5.66E-04	8.86E-03
OBSCN	-0.056510026	-0.402572078	10.10710162	-3.627529374	3.35E-04	6.20E-03
ATG16L2	2.121356852	-0.402184091	7.863786526	-4.228766717	3.10E-05	1.19E-03
LOC100132287	0.500477984	-0.401936364	8.387015584	-3.789485837	1.82E-04	4.00E-03
SOX10	-0.913411082	-0.40118539	1.644353409	-3.364517854	8.64E-04	1.18E-02
TNKS	3.660365726	-0.401018182	9.947505195	-4.611266746	5.88E-06	3.89E-04
SLC7A5P2	-0.214864116	-0.40005974	4.062535714	-3.58026557	3.99E-04	6.95E-03
LOC100128288	0.602988876	-0.399643506	5.081389935	-3.818611157	1.62E-04	3.71E-03
DNAH1	-1.722838227	-0.399359416	7.519306656	-3.097240733	2.13E-03	2.19E-02
CASKIN1	-2.446431062	-0.399269481	6.576626948	-2.838514053	4.83E-03	3.87E-02

MDN1	2.956693062	-0.399172403	9.387711851	-4.439962562	1.26E-05	6.43E-04
RNF207	-2.144565378	-0.399103571	8.225495617	-2.949050274	3.43E-03	3.08E-02
MICAL3	2.191501749	-0.398586364	10.56926526	-4.246857081	2.88E-05	1.14E-03
FOXK1	3.197954425	-0.397483442	10.23087971	-4.499335697	9.68E-06	5.42E-04
LOC91316	-0.491932978	-0.396969156	7.716914123	-3.496161204	5.42E-04	8.60E-03
C14orf139	-2.602511041	-0.396382792	4.650833929	-2.779732276	5.78E-03	4.36E-02
IFFO2	-0.48683873	-0.396286364	7.650065909	-3.497724337	5.39E-04	8.58E-03
DFNB59	-0.124110916	-0.395005195	4.368640909	-3.607421992	3.61E-04	6.48E-03
SPTBN2	-0.323705119	-0.394616883	11.04114351	-3.547445382	4.50E-04	7.56E-03
LRRN3	-1.590032062	-0.393869156	3.546688799	-3.142547402	1.84E-03	1.98E-02
CHKB-CPT1B	0.25116675	-0.393713312	8.172538149	-3.717788119	2.39E-04	4.86E-03
ATM	7.867718965	-0.393362662	9.311574513	-5.540284225	6.50E-08	1.69E-05
CBX6	0.349963955	-0.393187338	9.207594643	-3.746349785	2.14E-04	4.49E-03
PITPNM3	-0.607261068	-0.392827922	8.773193831	-3.460599647	6.15E-04	9.34E-03
PPP1R3E	1.737220364	-0.392775974	6.557275649	-4.128452935	4.71E-05	1.57E-03
ABCC2	-2.284950691	-0.392631169	2.523977922	-2.898140108	4.02E-03	3.42E-02
PPL	-1.705131679	-0.392606169	11.16480341	-3.10331709	2.09E-03	2.16E-02
SPDYE2	-0.280341687	-0.392246104	1.612574351	-3.560554577	4.29E-04	7.32E-03
CCDC57	3.026286258	-0.392098377	8.477194318	-4.457159985	1.17E-05	6.17E-04
TRIM66	0.376962239	-0.39201526	7.740795617	-3.754120412	2.08E-04	4.42E-03
KIAA1377	-2.403940696	-0.391941234	5.934577435	-2.854317715	4.61E-03	3.74E-02
MCC	-1.594438385	-0.391888636	8.073813474	-3.141054006	1.85E-03	1.99E-02
SRGAP3	1.065888107	-0.390606169	10.11780666	-3.947703921	9.79E-05	2.58E-03
MFHAS1	-0.064256878	-0.390326623	9.415118506	-3.625230311	3.38E-04	6.23E-03
SLC1A7	-2.309060462	-0.390168182	2.076971104	-2.88931159	4.14E-03	3.49E-02
CMTM4	4.232862744	-0.389662338	9.991918831	-4.746669297	3.18E-06	2.54E-04
BOD1L	7.804903444	-0.389481169	9.970528247	-5.527377512	6.95E-08	1.70E-05
LOC100133331	0.396522961	-0.389249026	7.786426136	-3.75974124	2.04E-04	4.35E-03
C12orf51	4.567684196	-0.388987013	10.32316818	-4.824340636	2.22E-06	1.94E-04
PDE7B	-0.959134328	-0.38824513	5.82855276	-3.349949464	9.09E-04	1.22E-02
PSD3	-1.002512126	-0.388230844	7.823807955	-3.33607388	9.54E-04	1.26E-02
SYNE1	-0.72507452	-0.38591461	8.14589724	-3.423920247	7.01E-04	1.02E-02
DNM3	-1.948709826	-0.385594805	6.645051948	-3.018722652	2.75E-03	2.63E-02
SOX9	-2.760959257	-0.385239935	10.52365795	-2.718826189	6.92E-03	4.95E-02
KIF21B	-1.094784689	-0.384602597	6.731074675	-3.306378342	1.06E-03	1.35E-02
CAPS2	-2.174564121	-0.384583766	3.792159416	-2.938241313	3.55E-03	3.14E-02
RORA	0.053977032	-0.383712987	7.387524675	-3.660175236	2.97E-04	5.69E-03
TTN	-2.092961101	-0.383675	6.486115747	-2.967557123	3.24E-03	2.95E-02
AGAP8	0.305916934	-0.38358961	5.893312338	-3.733640631	2.25E-04	4.66E-03
CBX7	0.856698987	-0.38328474	8.602074513	-3.889844757	1.23E-04	3.06E-03
ITGA7	-2.182280472	-0.383155519	8.406858604	-2.935454934	3.58E-03	3.16E-02
TAL1	-0.490562164	-0.383136039	3.447055357	-3.496581892	5.41E-04	8.60E-03
RASD2	-2.754083761	-0.383043506	5.937365909	-2.721495955	6.87E-03	4.93E-02
C9orf139	-1.545236213	-0.382077597	2.262901461	-3.157692158	1.75E-03	1.91E-02
NCRNA00203	-1.342302293	-0.381691883	1.76360276	-3.225467873	1.39E-03	1.64E-02

MLL	5.163275916	-0.380713961	10.89064432	-4.959932185	1.17E-06	1.28E-04
ANKRD36	0.613342431	-0.380353896	7.050100974	-3.821541609	1.61E-04	3.68E-03
SLC9A5	-1.719154732	-0.379611364	4.292557955	-3.098505718	2.12E-03	2.18E-02
C20orf117	0.849140681	-0.379220779	9.814861039	-3.887739691	1.24E-04	3.06E-03
OCLM	0.04472756	-0.378873377	1.938056169	-3.657452492	3.00E-04	5.73E-03
ZNF224	-0.288388815	-0.37814026	6.329338312	-3.558125203	4.33E-04	7.36E-03
PDXDC2	-0.512512824	-0.378003247	7.349373701	-3.489839864	5.54E-04	8.74E-03
ANKRD36B	-1.175299675	-0.376953247	5.202037013	-3.280262795	1.16E-03	1.43E-02
CDNF	-1.869555794	-0.376589935	3.926076461	-3.046454653	2.52E-03	2.47E-02
LOC100132832	-1.420168138	-0.376430844	1.99792289	-3.199620782	1.52E-03	1.75E-02
KIF13B	2.591963729	-0.376253247	8.899117532	-4.348860125	1.86E-05	8.56E-04
C3orf42	1.377460527	-0.375599675	4.04550211	-4.032493984	6.97E-05	2.04E-03
LOC100129726	-2.018165916	-0.37523474	3.760738474	-2.994189517	2.98E-03	2.77E-02
PPP1R12B	1.635471701	-0.375208766	8.885030682	-4.101516918	5.26E-05	1.66E-03
GARNL3	-0.836464982	-0.374368506	6.276025487	-3.388903478	7.93E-04	1.11E-02
FLJ10213	0.823739599	-0.37375974	4.98442013	-3.880657672	1.28E-04	3.11E-03
ZNF546	0.919435662	-0.373102273	6.125479383	-3.907278045	1.15E-04	2.91E-03
MUM1	3.216086576	-0.372942532	9.024670292	-4.503770298	9.49E-06	5.36E-04
ACACB	-0.1624752	-0.372441883	8.299386201	-3.595965038	3.76E-04	6.69E-03
HERC2P2	-1.991358462	-0.372437987	9.216310877	-3.003680732	2.89E-03	2.71E-02
CEP290	0.468075637	-0.372267532	7.380406494	-3.780237374	1.88E-04	4.12E-03
TRPM3	-2.172657519	-0.372077597	1.599257305	-2.938929403	3.54E-03	3.14E-02
ERICH1	2.485072701	-0.371817857	7.7793125	-4.321841431	2.09E-05	9.07E-04
ITGB1BP2	-2.198800242	-0.370195455	2.393151623	-2.929481229	3.65E-03	3.20E-02
C1orf104	0.837109596	-0.369842532	6.396495617	-3.884386785	1.26E-04	3.08E-03
C20orf165	-0.202292312	-0.369732143	3.468195617	-3.584038732	3.93E-04	6.88E-03
PPFIA2	-1.942047657	-0.369655519	1.686370942	-3.021065998	2.73E-03	2.61E-02
CREB5	-2.461409376	-0.368635714	6.797778247	-2.832923191	4.92E-03	3.91E-02
CRAMP1L	5.756727924	-0.368204545	9.734240584	-5.091985035	6.19E-07	8.04E-05
CEP110	1.582605571	-0.367402273	8.10182289	-4.08745912	5.57E-05	1.72E-03
DISC1	0.980263231	-0.366988961	7.193458766	-3.924114095	1.07E-04	2.76E-03
ANKRD23	0.549092248	-0.366878571	5.419811364	-3.803323305	1.72E-04	3.86E-03
TBC1D4	2.429694326	-0.366370455	8.279149838	-4.307784828	2.22E-05	9.39E-04
BREA2	-1.560733229	-0.365991234	2.855011526	-3.152460565	1.78E-03	1.93E-02
C9orf102	3.256510759	-0.365751948	5.806718182	-4.513643212	9.09E-06	5.18E-04
SLC26A1	-1.493801485	-0.365743506	6.042263961	-3.174998086	1.65E-03	1.84E-02
HERC1	8.27617382	-0.36548474	9.457856981	-5.623622847	4.21E-08	1.25E-05
SLC10A5	-0.753456005	-0.365340909	2.187056818	-3.415029749	7.23E-04	1.04E-02
DAB2IP	5.045720248	-0.364871429	10.69920325	-4.933420624	1.33E-06	1.39E-04
MYSM1	0.724912995	-0.363765909	5.831080032	-3.852992198	1.42E-04	3.36E-03
FLJ14107	-0.153610463	-0.363299351	1.604563312	-3.598615336	3.73E-04	6.65E-03
FMNL2	6.022551925	-0.363177922	10.22993636	-5.150210371	4.66E-07	6.71E-05
ZNF517	2.482924786	-0.362508442	8.729759416	-4.321296981	2.10E-05	9.07E-04
LOC389791	-2.316226978	-0.361888636	3.1047875	-2.886682435	4.17E-03	3.51E-02
EVPL	-1.916673176	-0.36183961	10.1264276	-3.029975587	2.65E-03	2.57E-02

ZNF250	3.043518201	-0.361464286	9.081846429	-4.461409276	1.14E-05	6.08E-04
TRIM56	2.496464277	-0.36123961	9.069969805	-4.324727941	2.07E-05	9.05E-04
CEMP1	2.658019694	-0.360731818	6.310610714	-4.365483377	1.74E-05	8.15E-04
TAS2R14	-1.859255873	-0.359720779	2.606668182	-3.050045854	2.49E-03	2.45E-02
SPDYE7P	-1.847874495	-0.359470455	2.204050487	-3.054009507	2.46E-03	2.43E-02
PLXNB1	1.306052009	-0.359435065	12.24402922	-4.013203261	7.53E-05	2.15E-03
SLC4A7	5.028204914	-0.359261364	8.94764724	-4.929460164	1.35E-06	1.40E-04
ANKRD44	-2.695212952	-0.359212338	5.353369805	-2.744254422	6.42E-03	4.70E-02
ZZEF1	5.793642211	-0.358971429	9.722535714	-5.100103907	5.95E-07	7.85E-05
HIC2	0.026127219	-0.358535065	7.491065584	-3.65197154	3.06E-04	5.82E-03
NBPF14	-0.351580244	-0.358516558	7.760535877	-3.53899491	4.64E-04	7.72E-03
FAM86B1	-2.425436669	-0.357530844	7.321659253	-2.846332991	4.72E-03	3.80E-02
C22orf23	0.594112821	-0.357240909	5.312274351	-3.816097271	1.64E-04	3.74E-03
ZC3H7B	8.876960304	-0.357026948	11.15436445	-5.744426945	2.22E-08	7.35E-06
C6orf174	-2.711004746	-0.356139286	4.025182955	-2.738167233	6.54E-03	4.76E-02
UNKL	3.969470635	-0.35611039	8.416896104	-4.684792975	4.22E-06	3.11E-04
PRH2	-0.664680672	-0.356046104	2.131176948	-3.442768007	6.56E-04	9.73E-03
AFAP1	0.108591969	-0.356009416	9.916271916	-3.676214546	2.79E-04	5.49E-03
H6PD	1.134859658	-0.355616234	11.41784058	-3.966613843	9.08E-05	2.44E-03
MYC	-2.401719936	-0.355497078	11.56000731	-2.855141416	4.59E-03	3.73E-02
C17orf69	0.904373657	-0.355455519	5.464415747	-3.90309902	1.17E-04	2.94E-03
HIVEP2	1.843773191	-0.35525974	10.45839481	-4.156493886	4.20E-05	1.46E-03
FAT4	-2.719651835	-0.354955195	6.499584091	-2.734828623	6.60E-03	4.79E-02
MYST3	5.443520562	-0.354682143	11.01416932	-5.022655816	8.66E-07	1.02E-04
TMOD2	-2.208566528	-0.354225325	6.88308474	-2.925944234	3.69E-03	3.22E-02
SHPRH	1.734891613	-0.35418539	7.614140422	-4.127838195	4.72E-05	1.57E-03
PDPK1	10.22191365	-0.353477597	10.15684692	-6.007807738	5.31E-09	2.32E-06
ZEB1	-1.950142301	-0.353474351	7.632962175	-3.018218571	2.76E-03	2.63E-02
FBXO21	-1.187811641	-0.352816234	11.61231591	-3.276187088	1.17E-03	1.44E-02
APC2	-2.352189062	-0.352780195	3.86593263	-2.873454809	4.34E-03	3.60E-02
COLQ	-1.88849295	-0.351776299	5.882635227	-3.039841568	2.57E-03	2.51E-02
HIST2H2BA	-2.336283702	-0.35137987	1.701218506	-2.879312229	4.27E-03	3.56E-02
TRIB2	-2.382965462	-0.351348377	10.07670244	-2.862088681	4.50E-03	3.67E-02
FAM160B2	5.403637317	-0.35100487	9.685483929	-5.0137697	9.04E-07	1.05E-04
NCRNA00182	1.552081905	-0.350438312	5.687167857	-4.079322778	5.76E-05	1.76E-03
BMS1P4	-1.428373643	-0.35028961	4.771553247	-3.196885662	1.53E-03	1.76E-02
PPM1F	3.269774559	-0.349785714	8.729070779	-4.516878569	8.96E-06	5.14E-04
C1orf152	-0.59214574	-0.34933474	3.323905357	-3.46527963	6.05E-04	9.27E-03
DIP2C	-1.559774063	-0.348093506	8.54936039	-3.152784603	1.78E-03	1.93E-02
ZNF37B	0.024806017	-0.347940909	8.080393182	-3.651581936	3.06E-04	5.82E-03
NF1	-0.875786967	-0.347894156	10.21336656	-3.376461998	8.29E-04	1.15E-02
SLC25A25	0.980647273	-0.347663636	8.655846104	-3.924220184	1.07E-04	2.76E-03
L3MBTL	-2.490662787	-0.347086364	7.715898377	-2.821973695	5.08E-03	4.00E-02
FAM21A	-0.998490499	-0.346807468	8.454325487	-3.337362558	9.50E-04	1.25E-02
ANKRD36BP1	-0.020000301	-0.34647987	7.541107468	-3.638346613	3.22E-04	6.04E-03

ATP8B1	-2.261089549	-0.345850649	9.655287013	-2.906852631	3.92E-03	3.36E-02
WDR31	-0.665682978	-0.34561039	5.400024026	-3.442455984	6.56E-04	9.74E-03
RAI1	4.111399092	-0.345179545	10.78506932	-4.718221672	3.62E-06	2.78E-04
BAT2L1	4.470975733	-0.344992208	12.6371026	-4.802016856	2.46E-06	2.10E-04
FYCO1	4.916929953	-0.344846753	9.748498701	-4.904236	1.52E-06	1.54E-04
ZBTB43	4.572765522	-0.344575	8.100138474	-4.825511148	2.20E-06	1.93E-04
ZC3H6	2.442584655	-0.344448701	8.175328896	-4.311060368	2.19E-05	9.33E-04
MLL2	1.012210295	-0.344068182	11.42284318	-3.932930479	1.04E-04	2.69E-03
FAM83H	-1.30937256	-0.343479221	12.22312143	-3.236340763	1.34E-03	1.60E-02
C3orf49	4.957566538	-0.343368182	0.707258766	-4.913460373	1.46E-06	1.49E-04
PER2	1.265939436	-0.343156818	8.896735227	-4.00233045	7.87E-05	2.20E-03
ZNF660	-2.507161863	-0.343088312	4.530512338	-2.815780328	5.18E-03	4.06E-02
AKAP13	4.121852164	-0.343070455	10.71310049	-4.720675647	3.58E-06	2.76E-04
NPY6R	-2.187727513	-0.342736039	1.259789123	-2.933486501	3.60E-03	3.17E-02
GRLF1	2.121368051	-0.342384091	11.00945341	-4.228769611	3.10E-05	1.19E-03
NPHP3	4.58659871	-0.342319156	8.044470942	-4.82869647	2.17E-06	1.91E-04
LOC100132724	2.198930918	-0.341603247	2.010125	-4.248769069	2.85E-05	1.13E-03
TNFRSF10A	-1.237324002	-0.341390909	6.023288312	-3.260012214	1.24E-03	1.50E-02
GOLGA6L9	-2.580624536	-0.340941234	7.692465747	-2.788045863	5.63E-03	4.30E-02
MAGI1	-1.317094275	-0.340525	9.20249789	-3.23379423	1.35E-03	1.61E-02
C7orf63	-2.370290459	-0.340512662	6.273105357	-2.866774889	4.43E-03	3.64E-02
KIAA1109	3.657473056	-0.339998377	9.379693669	-4.610573887	5.90E-06	3.89E-04
LOC285696	-1.44091893	-0.33969513	0.984986201	-3.192699745	1.56E-03	1.77E-02
ZCWPW1	-2.142455816	-0.339632468	6.163746104	-2.949808973	3.42E-03	3.07E-02
ZNF471	-1.453429119	-0.339622727	6.722791883	-3.188520431	1.58E-03	1.79E-02
NBEAL2	1.285280583	-0.339345455	10.60249286	-4.007576315	7.70E-05	2.18E-03
KIAA1908	-2.65638971	-0.339085065	6.503221753	-2.759165163	6.14E-03	4.56E-02
MPP3	-2.689147513	-0.338941234	5.710554058	-2.746589029	6.38E-03	4.68E-02
BTAF1	4.227175238	-0.338685065	9.11801461	-4.745340514	3.19E-06	2.54E-04
PRICKLE2	-1.193913156	-0.338606169	7.950364448	-3.27419784	1.18E-03	1.45E-02
SGK196	-1.421400544	-0.338474026	6.001288961	-3.199210127	1.52E-03	1.75E-02
FAM63B	1.848087429	-0.338317857	7.524128084	-4.157625694	4.18E-05	1.46E-03
LENG8	-1.511991975	-0.338271429	11.62968052	-3.168887739	1.68E-03	1.86E-02
TBC1D3	-1.246066716	-0.337975974	8.071336688	-3.257148349	1.25E-03	1.51E-02
LOC729603	-2.472840563	-0.337590909	4.241449351	-2.8286493	4.98E-03	3.94E-02
OMG	-0.769912654	-0.337462338	1.669692857	-3.409864892	7.37E-04	1.06E-02
ZNF667	-2.511981921	-0.33535487	7.238431006	-2.813968553	5.21E-03	4.08E-02
PTCH1	0.15246997	-0.33513474	8.733553409	-3.689054501	2.66E-04	5.28E-03
SAMD4A	-0.705935048	-0.334912338	7.910423052	-3.429903662	6.86E-04	1.01E-02
ACVR2B	-0.604839225	-0.333998377	8.958747565	-3.461349887	6.14E-04	9.33E-03
TACC1	-2.019120211	-0.333877597	9.894227435	-2.993851126	2.98E-03	2.77E-02
RALGPS2	-2.005742223	-0.333518831	8.638517208	-2.998591657	2.93E-03	2.74E-02
PDZK1P1	-2.399184338	-0.333467857	3.148982955	-2.856081619	4.58E-03	3.72E-02
ZBTB34	4.833935272	-0.333427597	8.171303084	-4.885350512	1.66E-06	1.66E-04
MAML2	-0.821044208	-0.333357143	9.517188961	-3.393771086	7.80E-04	1.10E-02

VAV2	-1.181119128	-0.333308442	9.604586039	-3.278367725	1.16E-03	1.44E-02
AGAP4	1.354628495	-0.333111688	7.148719481	-4.026335013	7.14E-05	2.07E-03
ZNF483	-2.453607752	-0.333084091	3.723690422	-2.835836558	4.87E-03	3.88E-02
LOC100170939	-0.701539045	-0.332284091	7.145121591	-3.431276588	6.83E-04	1.00E-02
FKBP1AP1	-1.783998911	-0.332046429	2.449406006	-3.076165678	2.29E-03	2.31E-02
LOC100189589	-2.585290773	-0.331981169	1.182722403	-2.786275368	5.66E-03	4.31E-02
NFAT5	1.582869182	-0.331922727	9.940598377	-4.087529325	5.57E-05	1.72E-03
UBE2Q2P1	-2.646173223	-0.331890584	3.523904708	-2.763076274	6.07E-03	4.53E-02
SPG11	7.559385769	-0.331853571	10.07516932	-5.476693964	9.03E-08	2.11E-05
MYCBP2	2.542962596	-0.331790909	9.820157143	-4.336492581	1.97E-05	8.84E-04
PCM1	3.73558679	-0.331578247	10.33699854	-4.629252339	5.42E-06	3.71E-04
TNRC6C	-2.575705937	-0.330775974	8.968375	-2.789910957	5.60E-03	4.29E-02
LOC90110	5.523189339	-0.330451948	7.40055	-5.040366736	7.95E-07	9.53E-05
CDC42EP1	-1.889697658	-0.330373052	11.3354401	-3.039420412	2.57E-03	2.51E-02
KLHDC1	-0.087632158	-0.329405844	5.511186039	-3.618285034	3.47E-04	6.31E-03
LOC100272146	-1.014440832	-0.32939513	5.206619968	-3.332248761	9.67E-04	1.27E-02
LOC642826	-0.682325027	-0.329023701	5.911393344	-3.437271412	6.69E-04	9.88E-03
TTC12	-1.031607327	-0.328783442	7.518008279	-3.326736904	9.85E-04	1.29E-02
MDM4	2.702226083	-0.328744156	9.639142857	-4.376577077	1.65E-05	7.87E-04
IFT140	1.267122371	-0.328630844	9.6342625	-4.002651472	7.86E-05	2.20E-03
FNDC8	0.229642094	-0.328417857	3.775210552	-3.711538931	2.45E-04	4.95E-03
SPEN	3.832602429	-0.328116558	11.53065666	-4.652360232	4.88E-06	3.42E-04
PLEKHH1	-2.407992306	-0.32709513	8.697125162	-2.852814354	4.63E-03	3.75E-02
D2HGDH	-0.204632591	-0.326768506	8.900535877	-3.583336623	3.94E-04	6.90E-03
ABLIM1	-1.638558505	-0.326211039	11.87248279	-3.126063959	1.94E-03	2.05E-02
LOC349114	-2.650977163	-0.326077597	6.579381331	-2.761237869	6.10E-03	4.54E-02
DIXDC1	0.619623897	-0.325801299	8.077180519	-3.823318512	1.59E-04	3.68E-03
STXBP1	2.204216106	-0.325401623	10.03902971	-4.250128812	2.84E-05	1.13E-03
NR2C2	5.155773826	-0.325332143	10.04833133	-4.958243875	1.18E-06	1.28E-04
ZFP106	5.759337608	-0.324794481	9.977390422	-5.092559361	6.17E-07	8.04E-05
PALM2-AKAP2	0.176498785	-0.324632468	9.146394156	-3.696068769	2.59E-04	5.19E-03
YPEL4	-2.0529162	-0.324497403	4.359697403	-2.981843815	3.09E-03	2.85E-02
NPFF	-1.425145179	-0.324401948	2.845528896	-3.197962058	1.53E-03	1.75E-02
ZNF175	0.57406477	-0.324386039	7.439640097	-3.810413708	1.68E-04	3.80E-03
KIF27	-0.957405864	-0.323846429	6.416196916	-3.350501257	9.07E-04	1.22E-02
CDRT1	-1.864768199	-0.323044156	1.249224026	-3.048124404	2.50E-03	2.46E-02
AKAP9	3.369968076	-0.322743831	9.864129383	-4.541253291	8.04E-06	4.80E-04
ALS2CR8	2.901655476	-0.322441558	7.20545974	-4.426320639	1.33E-05	6.71E-04
FAM186B	-1.281873577	-0.322348377	3.982875162	-3.245394489	1.30E-03	1.56E-02
ANKS6	3.630763684	-0.322156818	9.520629383	-4.60417213	6.07E-06	3.96E-04
LIMCH1	-2.287548107	-0.321792857	8.844002273	-2.897190208	4.04E-03	3.42E-02
TGOLN2	11.00156192	-0.321743182	12.32710698	-6.156412163	2.32E-09	1.11E-06
PDPR	-0.721235287	-0.321718182	9.80178961	-3.425121249	6.98E-04	1.02E-02
ZFYVE28	-2.366973302	-0.321686688	7.114933279	-2.868000114	4.42E-03	3.63E-02
SLC23A2	1.8788384	-0.321527597	9.765114773	-4.165685055	4.04E-05	1.44E-03

LOC650623	-2.233328792	-0.321116883	5.769235065	-2.916958095	3.80E-03	3.28E-02
ZNF772	-2.477629909	-0.321008117	7.817243994	-2.826856841	5.01E-03	3.96E-02
SLC22A13	-0.771922	-0.320970779	1.342602273	-3.409233771	7.38E-04	1.06E-02
CDK5RAP2	4.885747052	-0.320827597	10.10274269	-4.897147574	1.57E-06	1.58E-04
DPRXP4	-0.080002911	-0.320376299	2.403426136	-3.620553181	3.44E-04	6.28E-03
ZNF780A	-0.664674681	-0.320333442	7.580938474	-3.442769872	6.56E-04	9.73E-03
SNORA39	-1.922347695	-0.320223701	3.434378409	-3.027985266	2.67E-03	2.58E-02
UBR5	5.178646596	-0.319819805	11.01259399	-4.963389765	1.15E-06	1.27E-04
KLF6	-1.753845646	-0.319741558	11.34362857	-3.086572907	2.21E-03	2.25E-02
KLHL17	-2.077381446	-0.318883442	6.622682305	-2.973123113	3.18E-03	2.91E-02
LOC646471	-1.930281805	-0.31885487	6.470354383	-3.025200338	2.69E-03	2.59E-02
ZNF445	2.378004975	-0.318396429	9.176230357	-4.294627999	2.35E-05	9.77E-04
KDM6B	0.806989676	-0.318152922	10.29638198	-3.87598127	1.30E-04	3.15E-03
TNRC6A	7.885646787	-0.318132143	10.17426705	-5.54396338	6.37E-08	1.68E-05
MAP3K12	-0.84509351	-0.317965584	8.824934091	-3.386177035	8.01E-04	1.12E-02
FLJ37201	-2.165523653	-0.317951299	3.426780195	-2.941502662	3.51E-03	3.12E-02
TAF1	4.185119658	-0.317303247	9.420580195	-4.735505054	3.34E-06	2.64E-04
SBF1	0.689663808	-0.317201623	10.44849724	-3.843080997	1.48E-04	3.48E-03
CHD2	6.129680791	-0.317091883	10.50492679	-5.173520517	4.15E-07	6.23E-05
C14orf148	-2.356491963	-0.316746104	3.164667208	-2.87186825	4.36E-03	3.62E-02
MLL3	2.02964511	-0.31671526	11.11397679	-4.205010283	3.43E-05	1.27E-03
IQSEC1	3.184120708	-0.316574026	10.24632468	-4.495949804	9.83E-06	5.45E-04
FAM118A	1.134956548	-0.316035714	7.760612013	-3.96664035	9.08E-05	2.44E-03
HERC2	1.367916956	-0.314709091	10.05317857	-4.029920619	7.04E-05	2.05E-03
MLANA	-1.229200455	-0.314636688	1.617952435	-3.26267116	1.23E-03	1.49E-02
PPP1R3B	-1.847991426	-0.314550649	8.238405844	-3.05396881	2.46E-03	2.43E-02
SPDYE8P	-1.543218062	-0.314544481	6.319194643	-3.158372864	1.74E-03	1.91E-02
C5orf53	-0.713420115	-0.314055844	6.897852597	-3.427564817	6.92E-04	1.01E-02
ERN1	-1.263233748	-0.313883766	6.391280844	-3.251518085	1.28E-03	1.53E-02
TBC1D24	-0.935425578	-0.313643831	8.318739773	-3.357510884	8.85E-04	1.20E-02
EVC	-0.076419432	-0.313536039	9.36835211	-3.621618087	3.42E-04	6.28E-03
CDRT15P	-2.661158231	-0.313454221	2.473054708	-2.757337857	6.18E-03	4.57E-02
MYO18A	-2.065757287	-0.312704545	9.80534513	-2.977269576	3.14E-03	2.88E-02
MGAT5	-1.213677363	-0.312643831	8.156367045	-3.267746476	1.21E-03	1.48E-02
TSPYL6	-1.055897969	-0.312577597	1.175571591	-3.318923098	1.01E-03	1.31E-02
TET2	4.056236046	-0.312300325	8.602201786	-4.705253302	3.84E-06	2.91E-04
MYH9	0.967137506	-0.31207013	14.21652792	-3.92048663	1.09E-04	2.80E-03
KIAA0240	1.554953258	-0.312041883	9.538876461	-4.080088779	5.74E-05	1.75E-03
CCDC27	-0.877168962	-0.312038636	1.123046266	-3.376023963	8.30E-04	1.15E-02
ITPR1	-2.689649919	-0.311815909	7.734781006	-2.746395723	6.38E-03	4.68E-02
LOC100271832	-2.171282051	-0.311626623	1.277336688	-2.939425714	3.54E-03	3.14E-02
TAF1L	0.27070451	-0.311570455	4.268255682	-3.723452186	2.34E-04	4.79E-03
CHD9	4.26395806	-0.311016234	9.394900974	-4.753928517	3.07E-06	2.49E-04
MED13L	2.507979543	-0.310848052	10.46537208	-4.327644068	2.04E-05	8.95E-04
C17orf68	3.442902685	-0.310572403	8.751030032	-4.558925225	7.43E-06	4.58E-04

ZBTB47	0.250968882	-0.310394481	9.157644968	-3.717730717	2.39E-04	4.86E-03
CROCC	-1.298365928	-0.31007013	8.874112987	-3.2399674	1.33E-03	1.58E-02
NRIP2	-0.520744963	-0.310005844	5.974013312	-3.487308312	5.59E-04	8.78E-03
FRYL	5.682995775	-0.309964935	9.412475974	-5.075735897	6.70E-07	8.39E-05
ZKSCAN1	2.814830771	-0.30942987	9.830322727	-4.404724571	1.46E-05	7.18E-04
ZMYND17	-0.036838489	-0.308801623	6.004226461	-3.633361392	3.28E-04	6.10E-03
PLXNA3	-0.896670008	-0.308412013	11.01010828	-3.369837314	8.48E-04	1.17E-02
GIGYF1	1.804354808	-0.30823539	10.44914594	-4.146140038	4.38E-05	1.50E-03
R3HDM2	6.094345767	-0.308225	10.69367906	-5.16584168	4.31E-07	6.37E-05
ZNF862	-0.707217022	-0.30788961	9.339248052	-3.429503191	6.87E-04	1.01E-02
SOCS7	-1.492302724	-0.307827273	5.980066234	-3.175501043	1.65E-03	1.84E-02
GOLGA1	6.605032888	-0.307713961	8.710155357	-5.275915673	2.50E-07	4.46E-05
FBXW2	6.886986903	-0.307490584	10.31677354	-5.335879918	1.85E-07	3.65E-05
TNRC6B	4.482732507	-0.307481818	10.18129351	-4.804735475	2.43E-06	2.09E-04
PKD2	1.260946419	-0.307212013	8.793581006	-4.0009752	7.91E-05	2.21E-03
MAP3K5	-1.369773254	-0.306781494	7.869828734	-3.216371175	1.44E-03	1.68E-02
MLXIP	1.654793896	-0.306143506	10.44594513	-4.106644222	5.15E-05	1.63E-03
MAML3	-2.276746124	-0.305560065	8.79699789	-2.901138666	3.99E-03	3.40E-02
ASB14	-0.894726835	-0.30553539	2.965735552	-3.370454252	8.46E-04	1.16E-02
FAM168B	3.117396349	-0.305474675	10.98536591	-4.479587235	1.06E-05	5.72E-04
CIRBP	3.423153605	-0.30543961	10.84748734	-4.554145942	7.59E-06	4.63E-04
TOM1L2	2.477265957	-0.305161688	9.501445779	-4.3198623	2.11E-05	9.10E-04
PPP1R13L	-1.903033173	-0.304981494	9.518789773	-3.034754757	2.61E-03	2.54E-02
MAPKBP1	0.142317742	-0.304757792	8.644162662	-3.686087299	2.69E-04	5.32E-03
TTC28	-1.453860902	-0.304726948	10.1681112	-3.188376093	1.58E-03	1.79E-02
KATNAL1	-0.902959829	-0.304617857	7.164755357	-3.367839642	8.54E-04	1.17E-02
ZNF609	3.82083109	-0.304419156	10.52539302	-4.64956174	4.95E-06	3.44E-04
EP400	0.884147017	-0.304068831	10.66215519	-3.897480684	1.19E-04	2.99E-03
BICD2	4.017378766	-0.30294513	9.5532625	-4.696099782	4.00E-06	2.99E-04
HDAC4	-0.421799941	-0.302827273	9.090984416	-3.51762481	5.01E-04	8.13E-03
WDR81	-0.105848217	-0.302807792	9.50953961	-3.612864196	3.54E-04	6.40E-03
LOC150776	0.16326554	-0.302805519	8.68281737	-3.692207347	2.63E-04	5.23E-03
CREBBP	1.613650143	-0.302784416	11.67375584	-4.095719501	5.39E-05	1.69E-03
HEATR7A	-1.834864541	-0.302763312	10.70009886	-3.058534415	2.42E-03	2.41E-02
C20orf194	-0.697907389	-0.301883766	8.500071104	-3.432410415	6.80E-04	1.00E-02
ZNF618	1.068050224	-0.301581494	10.49646445	-3.948297945	9.76E-05	2.58E-03
RALGPS1	0.223345728	-0.30132013	8.652378896	-3.709709112	2.46E-04	4.97E-03
LOC100101266	-2.76306371	-0.301142532	2.239686526	-2.718008528	6.94E-03	4.96E-02
LOC652276	1.204171864	-0.300971429	5.512622727	-3.985535849	8.42E-05	2.32E-03
ZBTB26	2.251462757	-0.300881818	6.783724675	-4.262267112	2.70E-05	1.09E-03
CREBZF	0.438120891	-0.300835714	9.971679545	-3.771669206	1.95E-04	4.22E-03
ZNF696	-0.173466729	-0.30053961	8.251256818	-3.592676403	3.81E-04	6.73E-03
GTF2IRD2B	-1.003420256	-0.300238961	7.249464935	-3.335782818	9.55E-04	1.26E-02
TAS2R10	-1.409476405	-0.300046753	1.04222013	-3.203181361	1.50E-03	1.74E-02
PRO0628	-1.688528078	-0.299799026	2.543923214	-3.109004837	2.05E-03	2.13E-02

CCDC93	4.444392312	-0.299372403	9.0446125	-4.795864885	2.53E-06	2.15E-04
GNRHR	-2.541453061	-0.298536688	1.928568669	-2.802866718	5.39E-03	4.17E-02
PARD3B	-2.359418149	-0.297398701	6.770900649	-2.870788838	4.38E-03	3.62E-02
ATRX	5.25078204	-0.297229221	10.37402045	-4.979589236	1.06E-06	1.20E-04
ZNF34	0.232838181	-0.296893831	7.527945942	-3.712467447	2.44E-04	4.93E-03
KIAA0368	3.897198066	-0.296076948	10.73568231	-4.667691257	4.56E-06	3.25E-04
RNASE13	-2.347420072	-0.295948701	0.980955519	-2.875212255	4.32E-03	3.59E-02
CSAD	-2.567627355	-0.295805519	8.484888149	-2.792971727	5.55E-03	4.27E-02
RABGAP1	4.607179984	-0.295263636	9.969301299	-4.833432334	2.12E-06	1.89E-04
CLCN6	-0.329912347	-0.295194805	8.973648052	-3.545565234	4.53E-04	7.59E-03
RHBDF1	-1.095947376	-0.295174675	8.826180844	-3.306002582	1.06E-03	1.35E-02
ZNF84	-0.343255573	-0.295011039	9.045616558	-3.541520512	4.60E-04	7.65E-03
RNF113B	-1.502461184	-0.294881169	1.295480195	-3.172090588	1.67E-03	1.85E-02
RBMS2	1.808441576	-0.294737987	10.3825349	-4.14721456	4.36E-05	1.50E-03
FLYWCH1	3.852770303	-0.294547727	9.027246266	-4.657151521	4.78E-06	3.37E-04
DENND1A	6.973409832	-0.29387987	9.896356169	-5.354148745	1.69E-07	3.45E-05
SEC16B	-2.316566735	-0.293167208	7.157694968	-2.886557734	4.17E-03	3.51E-02
PILRB	-2.131943373	-0.293062662	9.767901136	-2.953587005	3.38E-03	3.05E-02
WDR35	-1.563004205	-0.292962662	8.62864724	-3.151693231	1.78E-03	1.94E-02
LOC200030	1.722840338	-0.292762987	8.662300325	-4.124655617	4.78E-05	1.58E-03
SRGAP1	-1.457669625	-0.292519156	9.551766396	-3.187102632	1.59E-03	1.79E-02
RASA4	-0.891950434	-0.292509416	8.478455682	-3.371335549	8.44E-04	1.16E-02
KIAA2018	1.781699366	-0.292093831	9.249816721	-4.140178806	4.49E-05	1.53E-03
LYST	-0.588373403	-0.292093831	7.690495292	-3.466446706	6.03E-04	9.25E-03
MYH10	-2.151292548	-0.291974675	11.54928994	-2.946629635	3.46E-03	3.09E-02
SEC1	-2.670675502	-0.29173474	2.636085877	-2.753687378	6.24E-03	4.61E-02
ZNF780B	-1.866928517	-0.291628247	7.764864448	-3.047371065	2.51E-03	2.46E-02
SMC5	2.606151209	-0.291620455	9.623236526	-4.35243517	1.84E-05	8.46E-04
EZH1	2.379952105	-0.291255195	9.008321753	-4.295124259	2.34E-05	9.77E-04
TMTC2	-1.833523078	-0.291056818	8.369519643	-3.059000624	2.42E-03	2.41E-02
CHD3	0.527075373	-0.29097987	12.02176591	-3.797062133	1.76E-04	3.93E-03
TSNARE1	-0.975296736	-0.290924351	8.514349513	-3.344785712	9.26E-04	1.23E-02
SHPK	1.998294395	-0.290883766	8.257080195	-4.196862041	3.55E-05	1.29E-03
EYS	-1.981602709	-0.290880844	2.990603084	-3.007127771	2.86E-03	2.69E-02
CERK	-0.55539438	-0.290573377	10.15931916	-3.476634286	5.81E-04	9.00E-03
ZNF337	0.81753437	-0.290504545	8.623796429	-3.878925831	1.28E-04	3.12E-03
ATXN7	3.440486257	-0.290306494	9.274981818	-4.558340683	7.45E-06	4.58E-04
NEIL2	-0.323474227	-0.290080844	8.50919724	-3.547515301	4.50E-04	7.56E-03
ZNF70	-0.03606567	-0.289953571	7.27886737	-3.633590335	3.28E-04	6.10E-03
IRGQ	-0.937308374	-0.289903896	9.273833766	-3.356910982	8.87E-04	1.20E-02
TXNIP	-2.583632287	-0.289558117	12.81687614	-2.786904765	5.65E-03	4.31E-02
SETD5	3.711079996	-0.28944026	11.36001818	-4.623399338	5.57E-06	3.78E-04
TBC1D14	3.296882531	-0.289177922	10.68040974	-4.523484581	8.70E-06	5.09E-04
SNX30	-0.310189547	-0.288825649	8.746661201	-3.55153604	4.43E-04	7.49E-03
ANKS3	0.950298961	-0.288662013	7.824156006	-3.915828644	1.11E-04	2.84E-03

TEP1	-0.171572599	-0.288510065	8.561576786	-3.593243318	3.80E-04	6.73E-03
DOCK9	-1.272539391	-0.287928571	9.991967532	-3.248462319	1.29E-03	1.54E-02
OGT	-0.307793455	-0.287826948	11.5346099	-3.552260796	4.42E-04	7.49E-03
PDGFC	-1.725649433	-0.287259416	9.710345617	-3.096274983	2.14E-03	2.19E-02
HEATR5B	5.705505431	-0.287236688	9.222582955	-5.080701215	6.54E-07	8.34E-05
LPIN1	0.147951569	-0.287100325	9.608781006	-3.687734172	2.67E-04	5.30E-03
STX2	-2.492725186	-0.286849351	7.381544805	-2.821200224	5.10E-03	4.01E-02
P2RY4	-1.170393888	-0.286684416	0.988806494	-3.281859544	1.15E-03	1.43E-02
RAPGEF2	2.372229705	-0.286626299	8.655343994	-4.293155772	2.36E-05	9.81E-04
INO80	5.093327187	-0.286567532	9.288333117	-4.944171617	1.26E-06	1.34E-04
NT5DC3	0.687175388	-0.286162662	7.892995292	-3.842380441	1.48E-04	3.48E-03
LCAT	-2.367152883	-0.285434416	5.986187338	-2.867933797	4.42E-03	3.63E-02
TRAF1	-1.673198986	-0.285011039	7.278476299	-3.114247345	2.02E-03	2.10E-02
ZFP41	-1.411357691	-0.284977597	9.134826786	-3.202555119	1.51E-03	1.74E-02
NCS1	-2.309828323	-0.284868182	10.17708734	-2.889029996	4.14E-03	3.49E-02
TBC1D16	-1.270708115	-0.284856494	8.834952273	-3.249063881	1.29E-03	1.54E-02
LUC7L	-0.339269898	-0.284835065	9.047632468	-3.542729131	4.58E-04	7.62E-03
SSH1	3.142836385	-0.284832143	10.26119172	-4.485831945	1.03E-05	5.64E-04
LATS2	-0.279088038	-0.284621104	8.731186851	-3.560932909	4.28E-04	7.32E-03
TBC1D5	3.056673674	-0.28449026	10.64351461	-4.464650954	1.13E-05	6.03E-04
DCUN1D3	-0.97210805	-0.284401623	7.595181656	-3.345805055	9.22E-04	1.23E-02
FLJ10038	-1.292420365	-0.284216234	7.811923052	-3.241924864	1.32E-03	1.57E-02
HCN3	-2.133561225	-0.284207792	6.83573961	-2.953005868	3.39E-03	3.05E-02
SLC6A6	-2.708396857	-0.284105195	9.823368831	-2.73917337	6.52E-03	4.75E-02
CXorf1	-2.58667271	-0.284077273	0.632046429	-2.785750819	5.67E-03	4.31E-02
ZNF621	3.934485765	-0.284042857	8.830521429	-4.676521308	4.38E-06	3.20E-04
LOC100129637	-1.633395395	-0.283638961	7.374316234	-3.127821627	1.93E-03	2.04E-02
CELSR2	-2.618603609	-0.28358474	12.19518101	-2.773604426	5.88E-03	4.43E-02
SLC24A1	-0.034707277	-0.283273701	8.134737175	-3.633992718	3.27E-04	6.10E-03
ARHGAP39	-1.02985612	-0.283111039	10.19003149	-3.327299574	9.83E-04	1.29E-02
STX16	2.47195239	-0.282814286	10.64358052	-4.31851477	2.12E-05	9.14E-04
KIAA1217	0.842467039	-0.282584091	11.8445086	-3.885880157	1.25E-04	3.08E-03
CACNB1	-2.399300691	-0.282273377	7.71681526	-2.856038481	4.58E-03	3.72E-02
KIAA0430	4.436268374	-0.282160714	10.49447679	-4.793983485	2.55E-06	2.16E-04
TRPV1	-2.308521519	-0.281983766	7.386569156	-2.889509219	4.13E-03	3.49E-02
ZFHX3	-1.156975582	-0.281936688	10.58001477	-3.286223286	1.13E-03	1.42E-02
PTPN14	-1.2464457	-0.280960065	8.706078409	-3.257024152	1.25E-03	1.51E-02
UBR1	3.350210365	-0.280506494	8.491367532	-4.536455729	8.22E-06	4.89E-04
BRD1	1.809276263	-0.280420779	9.387251299	-4.147433991	4.36E-05	1.50E-03
ZNF782	-0.09521072	-0.280051623	5.931671591	-3.616030667	3.50E-04	6.34E-03
LRRC37A	-1.906680153	-0.279931818	7.520825	-3.033477625	2.62E-03	2.55E-02
HTT	1.679461425	-0.279100974	10.74033003	-4.113181664	5.02E-05	1.61E-03
ZNF594	-2.543452993	-0.279062338	6.606119481	-2.802111829	5.40E-03	4.18E-02
ANKMY1	-1.927776121	-0.278482792	7.889481981	-3.026080111	2.69E-03	2.59E-02
TAOK1	-1.411769793	-0.27818539	7.372383279	-3.202417924	1.51E-03	1.74E-02

KIAA0556	2.379540011	-0.278146429	9.356548214	-4.295019234	2.35E-05	9.77E-04
ZNF841	-0.831653519	-0.2778	7.345370779	-3.390422923	7.89E-04	1.11E-02
C17orf86	-1.996426618	-0.277578571	7.418779545	-3.001888509	2.90E-03	2.73E-02
NEK6	-0.735774206	-0.277296104	9.861305195	-3.420571079	7.09E-04	1.03E-02
MTMR10	0.370501747	-0.277246104	9.012684091	-3.75226229	2.09E-04	4.43E-03
KIAA1958	-1.633958629	-0.277114286	6.436034416	-3.127629931	1.93E-03	2.04E-02
CRIM1	-1.408059712	-0.276890584	10.37497484	-3.203652873	1.50E-03	1.74E-02
FAM116B	-1.709906949	-0.276870779	7.993458117	-3.101679459	2.10E-03	2.17E-02
MAP1D	-1.556484784	-0.276656169	6.072788799	-3.153895593	1.77E-03	1.93E-02
MTX3	0.556472599	-0.276562013	8.463089123	-3.805420042	1.71E-04	3.84E-03
NIPSNAP3B	-1.601964132	-0.276327597	5.019669318	-3.138501834	1.86E-03	2.00E-02
PAN2	-1.424270138	-0.276220455	9.720396266	-3.198253746	1.53E-03	1.75E-02
RFX7	1.01586762	-0.276165909	9.075759903	-3.93393865	1.03E-04	2.69E-03
MLLT4	-2.369091383	-0.27613961	11.38213344	-2.867217837	4.43E-03	3.63E-02
DHFRL1	0.396417113	-0.276030844	7.274634578	-3.759710845	2.04E-04	4.35E-03
ZNF532	0.303237816	-0.275889935	10.44551737	-3.732866338	2.26E-04	4.66E-03
JMJD1C	0.900237822	-0.275476623	9.756522078	-3.901950807	1.17E-04	2.95E-03
SMG6	1.022307648	-0.275320455	9.682920942	-3.93571333	1.03E-04	2.68E-03
PLEKHA7	-2.217962269	-0.275106169	9.256327435	-2.922537623	3.73E-03	3.24E-02
BDP1	-0.776196612	-0.275073701	9.100531331	-3.407890787	7.42E-04	1.06E-02
ATP2B1	0.89387597	-0.274996753	10.51003344	-3.900183998	1.18E-04	2.96E-03
VPS53	0.416978581	-0.274596104	9.641582468	-3.765611062	1.99E-04	4.28E-03
PTPN3	-1.486363391	-0.274253896	9.228023701	-3.177493445	1.64E-03	1.83E-02
NF2	0.366516877	-0.274024026	9.871943831	-3.751115772	2.10E-04	4.44E-03
FNBP1	-0.951698968	-0.273997727	10.7584836	-3.352322521	9.02E-04	1.21E-02
BAZ2A	3.274002439	-0.273811039	11.66500162	-4.517909426	8.92E-06	5.14E-04
SASH1	-1.678040449	-0.273576623	9.263615584	-3.112592471	2.03E-03	2.11E-02
PER1	-2.694744003	-0.273573052	9.326289448	-2.744434989	6.42E-03	4.70E-02
KLF13	-0.333618164	-0.27311039	11.02208312	-3.544442318	4.55E-04	7.61E-03
IKZF4	0.072858926	-0.272879221	8.551116234	-3.665727717	2.91E-04	5.63E-03
SMCR8	3.670029826	-0.272788961	9.778071753	-4.61358086	5.82E-06	3.89E-04
PTAR1	2.522892951	-0.272701623	9.371625162	-4.33141817	2.01E-05	8.87E-04
HSFX2	-2.392914704	-0.272577597	5.011450162	-2.858405149	4.55E-03	3.70E-02
MLL5	0.459644595	-0.272321753	11.02844432	-3.777827568	1.90E-04	4.14E-03
LPCAT2	-1.844275757	-0.272165909	8.925615097	-3.055261791	2.45E-03	2.43E-02
ZNF169	-0.631865085	-0.272082792	7.062656656	-3.452969282	6.32E-04	9.49E-03
HOOK3	2.170776476	-0.272025325	9.863391234	-4.241519147	2.94E-05	1.15E-03
ZFYVE20	6.344179233	-0.271901623	9.28772711	-5.219932082	3.30E-07	5.30E-05
MYO9A	-0.628656102	-0.271815584	8.256951948	-3.453965356	6.30E-04	9.47E-03
RHOBTB2	-0.600084581	-0.271781494	9.099029383	-3.462822347	6.10E-04	9.31E-03
MTMR11	-2.201635905	-0.271718831	7.663356818	-2.92845467	3.66E-03	3.20E-02
ATP7A	-0.618155763	-0.27165974	8.233492208	-3.457222827	6.23E-04	9.39E-03
TRIOBP	1.84551854	-0.271397078	10.53321769	-4.156951798	4.19E-05	1.46E-03
SCAI	2.11973887	-0.271004545	8.375916558	-4.228348631	3.11E-05	1.19E-03
NFIA	-2.560477942	-0.270967857	10.54377386	-2.795677806	5.51E-03	4.24E-02

SMG1	2.823853823	-0.270900649	10.84143149	-4.40697321	1.45E-05	7.12E-04
YOD1	0.401527858	-0.270750974	8.101691396	-3.761178185	2.02E-04	4.34E-03
CYB561D1	-0.58898858	-0.270697078	8.306513149	-3.466256409	6.03E-04	9.25E-03
BPTF	1.508348517	-0.270405844	10.74314318	-4.06763993	6.04E-05	1.83E-03
VASH1	-2.161070705	-0.270302922	9.127184903	-2.943107811	3.50E-03	3.11E-02
TMEM170B	-1.254790356	-0.26989513	7.39317776	-3.254288394	1.26E-03	1.52E-02
PIP5K1P1	-1.597561789	-0.269850974	1.33267776	-3.139995016	1.85E-03	1.99E-02
GCC2	3.667627973	-0.269582468	9.488176948	-4.613005819	5.83E-06	3.89E-04
VPS13C	1.558807609	-0.269368506	9.36780211	-4.081116816	5.72E-05	1.75E-03
DLG5	-1.695159013	-0.269285065	10.92753019	-3.106734507	2.07E-03	2.14E-02
KIAA0528	-1.411682276	-0.269155195	9.714792532	-3.20244706	1.51E-03	1.74E-02
ZSCAN29	2.024047794	-0.268723052	8.41720276	-4.203556535	3.45E-05	1.28E-03
LOC100131496	-2.717628781	-0.268704221	3.71296737	-2.735610064	6.59E-03	4.78E-02
N4BP2	-2.533326299	-0.267790584	6.474356006	-2.805932244	5.34E-03	4.15E-02
PHF2	2.415928228	-0.26774513	9.878621591	-4.30428432	2.26E-05	9.47E-04
CCNL2	-2.448263034	-0.267451623	10.80258718	-2.837830804	4.84E-03	3.87E-02
GOLGB1	2.830023758	-0.266940909	11.13705357	-4.408510243	1.44E-05	7.11E-04
UNC119B	-1.490537834	-0.266639286	9.942942695	-3.176093213	1.64E-03	1.84E-02
SENP7	1.309415391	-0.266616883	8.34402987	-4.01411373	7.50E-05	2.14E-03
DENND4A	2.491223512	-0.266526948	7.991670292	-4.323400195	2.08E-05	9.07E-04
LOC285740	-0.266400223	-0.266525325	0.75465487	-3.564759816	4.22E-04	7.22E-03
SH3BP2	-2.065876488	-0.266462013	10.82387029	-2.977227083	3.14E-03	2.88E-02
ATXN1	-1.11109551	-0.266236688	9.630438799	-3.301103335	1.08E-03	1.37E-02
RGS12	-0.799101972	-0.266090909	9.107659091	-3.400686098	7.61E-04	1.08E-02
MNT	5.041090217	-0.265200325	10.2196336	-4.932373972	1.33E-06	1.39E-04
BAZ2B	-1.693666482	-0.264702922	9.346125162	-3.107245662	2.06E-03	2.14E-02
MECP2	2.799976368	-0.264622078	10.63725325	-4.401020503	1.49E-05	7.23E-04
C22orf43	-2.594191223	-0.264450649	0.970658442	-2.782895335	5.72E-03	4.33E-02
XPC	5.304874986	-0.2642	9.509218831	-4.991707728	1.00E-06	1.15E-04
SKI	-0.948366338	-0.263790584	11.75083198	-3.353385653	8.98E-04	1.21E-02
SNAPC4	0.090431222	-0.263647403	9.138634091	-3.670888201	2.85E-04	5.56E-03
ZNF236	-0.087692256	-0.263368506	8.118451461	-3.618267162	3.47E-04	6.31E-03
NEK9	1.422677775	-0.262551948	10.06409675	-4.044666593	6.63E-05	1.95E-03
BBC3	-1.583713292	-0.262416558	6.998967695	-3.14468781	1.83E-03	1.97E-02
RANBP10	2.760494186	-0.262365584	9.30586526	-4.391161972	1.55E-05	7.46E-04
KIAA0232	0.88694488	-0.262238636	9.642933279	-3.898258279	1.19E-04	2.98E-03
NCRNA00169	-2.729850624	-0.262023701	1.841456981	-2.730885919	6.68E-03	4.82E-02
LOC151162	-1.169138239	-0.261986364	10.12344838	-3.28226812	1.15E-03	1.43E-02
AGAP5	-2.722565854	-0.261817532	5.55973474	-2.733702657	6.63E-03	4.80E-02
TMX3	0.99274323	-0.261130844	8.992036526	-3.927560304	1.06E-04	2.74E-03
TRIM52	-0.973800063	-0.261085714	7.095377922	-3.345264197	9.24E-04	1.23E-02
MTMR3	-0.013204229	-0.26099513	10.01096412	-3.640356933	3.19E-04	6.03E-03
TSGA10	-2.70803374	-0.260856169	6.86796737	-2.739313435	6.52E-03	4.75E-02
DFNB31	-2.569558919	-0.260700974	8.090961201	-2.792240196	5.56E-03	4.27E-02
ZNF37A	-0.852632652	-0.260566883	8.769521104	-3.383793152	8.08E-04	1.13E-02

PLEKHM3	-0.626959393	-0.260499026	8.516881656	-3.45449191	6.29E-04	9.46E-03
GGA1	3.631734251	-0.26022987	9.978576623	-4.604404892	6.06E-06	3.96E-04
NUMA1	-0.314686234	-0.259958766	13.13920179	-3.550175542	4.45E-04	7.51E-03
PTK2B	-2.524710893	-0.259859416	8.962813149	-2.809178629	5.29E-03	4.12E-02
SMYD4	0.041584478	-0.259791558	7.820403571	-3.656526849	3.01E-04	5.73E-03
LIMD1	3.12543454	-0.259573377	9.452868506	-4.481561173	1.05E-05	5.72E-04
RABL2A	-1.505582676	-0.259248701	8.476166558	-3.171041935	1.67E-03	1.85E-02
HIVEP1	-0.163180995	-0.25910974	9.651395779	-3.595753949	3.77E-04	6.69E-03
ZFC3H1	1.678437907	-0.258577597	9.177415747	-4.112910593	5.02E-05	1.61E-03
SSH2	0.094461459	-0.258572727	8.455507143	-3.672070834	2.84E-04	5.54E-03
REL	-1.074135049	-0.258497727	5.82720763	-3.313045355	1.03E-03	1.33E-02
C22orf30	0.564956269	-0.258392532	9.867536201	-3.807828935	1.69E-04	3.81E-03
MZF1	-1.872163655	-0.258325974	9.034214935	-3.045544757	2.52E-03	2.47E-02
ANKRD28	1.494998497	-0.258106818	10.23479334	-4.064067652	6.13E-05	1.85E-03
SBF2	1.609342095	-0.257860065	9.317388149	-4.094574099	5.41E-05	1.69E-03
EML5	-2.113443333	-0.257844805	5.337923052	-2.960224632	3.31E-03	3.00E-02
AGAP6	-2.525560305	-0.257808117	7.209493344	-2.808858719	5.29E-03	4.12E-02
TAS2R19	-2.568371444	-0.257376299	0.947033279	-2.792689943	5.56E-03	4.27E-02
INPP5F	0.751318223	-0.257367532	8.628455195	-3.860401611	1.38E-04	3.29E-03
ADCY6	-1.079816509	-0.257351948	9.429723377	-3.311212263	1.04E-03	1.33E-02
AKNA	-2.122843663	-0.257250974	9.52022776	-2.956853645	3.35E-03	3.02E-02
ATF7	3.486095175	-0.256866883	10.53039838	-4.569362684	7.10E-06	4.43E-04
ZNF268	-0.62694802	-0.256751623	8.147574188	-3.454495439	6.29E-04	9.46E-03
LOC642852	-2.690313658	-0.256740584	8.337311526	-2.746140323	6.39E-03	4.68E-02
RC3H2	1.438646763	-0.256645455	8.521828571	-4.048957657	6.52E-05	1.93E-03
RAD54L2	-0.726326144	-0.25622013	8.107747078	-3.423528626	7.02E-04	1.02E-02
ANKFY1	2.089597166	-0.255828247	10.66118912	-4.220553309	3.21E-05	1.22E-03
MTMR15	0.515393011	-0.255277597	8.514835227	-3.79373609	1.79E-04	3.96E-03
ATG2B	1.017086249	-0.255121429	9.240150974	-3.934274523	1.03E-04	2.69E-03
RECK	-2.767331054	-0.255107792	7.102072727	-2.716349782	6.97E-03	4.98E-02
CCNT2	1.670137685	-0.255068506	9.262581331	-4.110711756	5.07E-05	1.61E-03
TMED10P1	-1.932476909	-0.255004545	5.713810065	-3.024429415	2.70E-03	2.60E-02
ABL1	-0.080712645	-0.25491039	11.04360195	-3.620342235	3.44E-04	6.28E-03
ZNF550	-0.982212219	-0.254823052	7.794439123	-3.342574014	9.33E-04	1.24E-02
YPEL1	-2.208094819	-0.254778247	8.350671916	-2.926115163	3.69E-03	3.22E-02
ZCCHC14	-1.49142807	-0.254741883	9.600905682	-3.175794527	1.65E-03	1.84E-02
LOC100129550	-1.571086136	-0.254676623	7.613387013	-3.148961029	1.80E-03	1.95E-02
SYNGAP1	-2.739237099	-0.254373052	10.06112451	-2.727252454	6.75E-03	4.86E-02
PRPF8	0.311052988	-0.253849675	12.78982516	-3.735124594	2.24E-04	4.64E-03
L3MBTL3	-1.908498387	-0.253770779	7.346218506	-3.03284071	2.63E-03	2.55E-02
C19orf34	-1.273834883	-0.253377597	0.715583279	-3.248036696	1.29E-03	1.55E-02
KIAA1147	-0.739837709	-0.253362338	10.15159091	-3.419298345	7.13E-04	1.03E-02
CDC42EP3	-1.454600641	-0.253089935	9.425713474	-3.188128796	1.58E-03	1.79E-02
DENND4C	0.263740861	-0.252955195	9.634960714	-3.7214343	2.36E-04	4.81E-03
NCOR2	-1.676394551	-0.252944805	12.81868929	-3.113155152	2.03E-03	2.11E-02

ZNF7	-0.054809515	-0.252836364	9.56519026	-3.628033863	3.34E-04	6.20E-03
ITPKB	-1.999512032	-0.252729545	10.26578068	-3.000796939	2.91E-03	2.73E-02
ZNF192	-2.736557724	-0.252608117	9.263111526	-2.728290099	6.73E-03	4.85E-02
CEP170	-1.177365551	-0.252496753	9.172075649	-3.279590171	1.16E-03	1.43E-02
EPN2	2.621133781	-0.252394156	10.17997955	-4.356207766	1.81E-05	8.35E-04
UBR4	2.120758552	-0.252169156	11.79807776	-4.228612121	3.11E-05	1.19E-03
LZTR1	-0.227470344	-0.252105844	9.662623701	-3.576478412	4.04E-04	7.00E-03
RBM9	0.942670169	-0.251679545	10.65860308	-3.913716674	1.12E-04	2.86E-03
DYNC1LI2	2.525618751	-0.251648377	10.61087776	-4.332107669	2.00E-05	8.87E-04
RPS10P7	-2.723989218	-0.251415909	5.937016721	-2.733152515	6.64E-03	4.80E-02
DISP1	-0.120365176	-0.251242208	7.886032792	-3.608538819	3.59E-04	6.47E-03
ZHX3	-2.319938373	-0.251228896	8.735414773	-2.885319958	4.19E-03	3.52E-02
C22orf46	-1.689045852	-0.251039286	7.734012175	-3.108827615	2.05E-03	2.13E-02
NCOR1	1.726742159	-0.250994805	10.84974091	-4.125686274	4.76E-05	1.58E-03
ASH1L	2.536561572	-0.250847403	10.78045097	-4.334874714	1.98E-05	8.85E-04
CCDC55	1.899173017	-0.250633117	8.738139935	-4.171006839	3.95E-05	1.42E-03
SGK269	-0.437564453	-0.250411039	9.889254221	-3.512810735	5.10E-04	8.23E-03
CTDSPL	-0.105741313	-0.250130519	10.44618279	-3.612896031	3.54E-04	6.40E-03
FAM179B	0.509466235	-0.250028247	8.797781331	-3.792047687	1.80E-04	3.97E-03
ARID5B	-1.994850646	-0.24998539	10.8580138	-3.002445919	2.90E-03	2.72E-02
ING5	-1.473016512	-0.249932468	8.811192857	-3.181966521	1.61E-03	1.81E-02
ZNF512B	-0.989149099	-0.249887662	10.53648864	-3.34035411	9.40E-04	1.24E-02
FLNB	-1.637356319	-0.249757468	12.12950633	-3.1264733	1.94E-03	2.05E-02
ZNF141	-1.467897291	-0.249726623	6.490400325	-3.18368062	1.60E-03	1.80E-02
METT10D	0.83558358	-0.249451948	9.328321429	-3.883961317	1.26E-04	3.08E-03
WDR6	-1.206193504	-0.249245779	11.60745308	-3.270190724	1.20E-03	1.47E-02
VPS13D	-1.222974416	-0.249244481	10.07187192	-3.264707662	1.22E-03	1.49E-02
USP20	2.570748927	-0.249225649	9.25155276	-4.343509462	1.91E-05	8.66E-04
ANKRD11	0.19338969	-0.249077597	11.34170341	-3.700992148	2.54E-04	5.11E-03
ZNF707	-2.391587482	-0.248936364	8.418418182	-2.85889679	4.54E-03	3.70E-02
RNF38	0.042299867	-0.248904221	10.10756737	-3.656737551	3.00E-04	5.73E-03
PIP4K2B	1.313449623	-0.248821429	10.76785357	-4.015205554	7.47E-05	2.14E-03
RAB11FIP3	-0.930319504	-0.248818831	9.577926948	-3.359137297	8.80E-04	1.19E-02
DOCK1	-2.204970983	-0.24879513	9.864129058	-2.927246879	3.68E-03	3.21E-02
HPS4	1.629709526	-0.248418506	9.32895763	-4.09998677	5.29E-05	1.66E-03
VPS13B	-0.183786059	-0.248292532	9.497342045	-3.589586375	3.85E-04	6.79E-03
GAPVD1	3.511902723	-0.248227273	9.628742857	-4.575589205	6.90E-06	4.35E-04
EXTL3	-2.121514947	-0.248084416	10.00985065	-2.957330346	3.34E-03	3.02E-02
SH3D19	-1.923569487	-0.247965584	9.628383442	-3.027556566	2.67E-03	2.58E-02
ZNF276	-2.148278892	-0.247762338	8.461818182	-2.947714272	3.45E-03	3.08E-02
DEPDC5	0.671223301	-0.247547727	8.286211201	-3.837886755	1.51E-04	3.54E-03
HEXDC	-2.190970507	-0.247489935	8.459698539	-2.932313967	3.62E-03	3.18E-02
APBB2	-2.296404304	-0.247252273	9.502610877	-2.893949197	4.08E-03	3.45E-02
ARL13A	1.192531051	-0.247181818	0.630864935	-3.982363591	8.52E-05	2.33E-03
MCPH1	-0.652244875	-0.246193506	8.307414286	-3.446637162	6.47E-04	9.65E-03

ASXL2	-0.379167983	-0.245112013	9.481283604	-3.530613262	4.78E-04	7.88E-03
KIAA0649	-0.641185079	-0.24505487	9.614253084	-3.450074837	6.39E-04	9.57E-03
SEPSECS	-0.151691863	-0.244989935	7.776354383	-3.599188706	3.72E-04	6.65E-03
GTPBP1	2.009317629	-0.244651299	10.38500162	-4.199728647	3.51E-05	1.29E-03
DDX26B	-2.208724555	-0.244586364	7.369382792	-2.925886969	3.69E-03	3.22E-02
CYTSA	-0.620532109	-0.244582792	10.03263328	-3.456485872	6.24E-04	9.41E-03
SCRIB	-2.450540624	-0.244478571	12.24024578	-2.836981141	4.86E-03	3.87E-02
C22orf29	-2.570676462	-0.244386688	9.856351461	-2.791816872	5.57E-03	4.27E-02
LRSAM1	1.246722958	-0.244270779	8.857193831	-3.99711228	8.04E-05	2.24E-03
WDFY3	1.037857623	-0.244165584	9.726679545	-3.939995471	1.01E-04	2.65E-03
FAM119B	0.769977769	-0.24405974	8.167266234	-3.86562981	1.35E-04	3.25E-03
CAMSAP1	1.150129922	-0.243599675	9.668371591	-3.970789535	8.93E-05	2.42E-03
LOC440944	-2.330062651	-0.243519805	8.470534253	-2.881600174	4.24E-03	3.54E-02
ARHGEF10L	-2.661474859	-0.243338961	8.989942208	-2.757216484	6.18E-03	4.57E-02
DNAJC27	1.016859751	-0.243192208	7.122284416	-3.934212099	1.03E-04	2.69E-03
LDHAL6B	-2.214850161	-0.243086688	1.179747565	-2.923666395	3.72E-03	3.23E-02
IKBKB	-0.750818666	-0.243028571	9.791476623	-3.415856796	7.21E-04	1.04E-02
CLASP1	0.084960866	-0.242770455	10.37768166	-3.669282424	2.87E-04	5.58E-03
C3orf48	-0.783669598	-0.242687662	0.823028247	-3.405541771	7.48E-04	1.07E-02
SNTB2	-0.177937654	-0.242518831	9.597970455	-3.591337925	3.83E-04	6.75E-03
SNX29	-1.46721503	-0.242377597	9.014854058	-3.183909001	1.60E-03	1.80E-02
MMP19	-1.747654717	-0.242301623	8.775321916	-3.088705601	2.19E-03	2.24E-02
RANBP2	1.339524311	-0.242099026	10.57104205	-4.022255995	7.26E-05	2.09E-03
TSTD2	1.355600411	-0.241914935	8.937752922	-4.026597361	7.14E-05	2.07E-03
ZNF33A	-1.066962108	-0.241708117	10.0713349	-3.315358322	1.02E-03	1.32E-02
RG9MTD3	-0.151422139	-0.241706494	7.044845455	-3.599269306	3.72E-04	6.65E-03
ANKRD26	-2.337861044	-0.241620455	7.46635276	-2.878731849	4.27E-03	3.57E-02
SCAPER	-0.262154033	-0.241530519	8.051330844	-3.566039713	4.20E-04	7.21E-03
ZBTB40	-0.338533376	-0.241351948	9.336499351	-3.542952433	4.57E-04	7.62E-03
YPEL2	-2.299529171	-0.241351299	9.125148377	-2.892804803	4.09E-03	3.46E-02
ZBTB4	-1.470668344	-0.240998701	11.12346039	-3.182752879	1.61E-03	1.81E-02
THOC2	4.384085832	-0.240996429	10.05241607	-4.781883572	2.70E-06	2.25E-04
FGD4	-2.704914062	-0.240953896	8.823689935	-2.740516503	6.49E-03	4.74E-02
CLUAP1	-0.92292546	-0.240829221	8.62384513	-3.361491194	8.73E-04	1.19E-02
ZKSCAN2	-2.642158045	-0.240445455	7.515005844	-2.76461194	6.04E-03	4.52E-02
LRBA	-1.686317519	-0.240438312	10.2591737	-3.109761353	2.05E-03	2.12E-02
UBN1	0.618757281	-0.240269481	10.57741851	-3.823073408	1.60E-04	3.68E-03
KIAA0467	-1.161245092	-0.240263636	10.29204286	-3.284835391	1.14E-03	1.42E-02
CLK1	-1.202998075	-0.240019805	9.912550487	-3.271233842	1.19E-03	1.47E-02
C11orf95	-2.336362606	-0.239207468	9.292933929	-2.879283199	4.27E-03	3.56E-02
PRR12	-1.148329401	-0.238687013	10.97607013	-3.289032242	1.12E-03	1.41E-02
PRO0611	-2.265641266	-0.238636364	0.958113636	-2.905192548	3.94E-03	3.37E-02
MICALL1	-1.668816083	-0.238458442	9.690262987	-3.115744766	2.01E-03	2.10E-02
DIP2A	-1.859701526	-0.238361364	9.277225812	-3.049890554	2.49E-03	2.45E-02
ZNF33B	-2.253761184	-0.23829513	9.189331006	-2.909523521	3.88E-03	3.34E-02

PHLPP2	-2.755643495	-0.238055195	7.480932792	-2.720890526	6.88E-03	4.94E-02
GOLGA2	-0.790178272	-0.237825649	10.37204367	-3.40349465	7.54E-04	1.07E-02
TET3	-2.569765875	-0.237755519	9.825043669	-2.792161806	5.56E-03	4.27E-02
DMXL2	-1.958690053	-0.237683766	8.478900325	-3.015209012	2.78E-03	2.65E-02
RGPD6	-2.432118829	-0.237659416	8.761531331	-2.843846564	4.76E-03	3.82E-02
TRIO	-1.969437427	-0.237316234	10.73355422	-3.011420978	2.82E-03	2.67E-02
AFG3L1	-2.446480474	-0.23715974	7.447765584	-2.838495627	4.83E-03	3.87E-02
NIN	0.431546675	-0.237001623	8.850142045	-3.769786369	1.96E-04	4.24E-03
STAT5B	0.997639528	-0.236848377	9.642340747	-3.928911617	1.05E-04	2.73E-03
RAPGEF1	-0.379099694	-0.236787338	10.81553425	-3.530634032	4.78E-04	7.88E-03
SHANK3	-1.973246066	-0.236472078	9.041510065	-3.010077504	2.83E-03	2.67E-02
HIPK1	-0.686740356	-0.236416234	10.47254058	-3.435894673	6.72E-04	9.89E-03
FOXJ2	-0.921997445	-0.23625974	9.849512338	-3.36178652	8.72E-04	1.19E-02
FBXW8	-1.356193772	-0.236114935	9.377433442	-3.220870861	1.42E-03	1.66E-02
ZNF264	-1.324822264	-0.236058117	9.480837175	-3.231243755	1.37E-03	1.62E-02
REST	-1.133079533	-0.235939286	9.437823864	-3.293981165	1.10E-03	1.39E-02
DDHD1	-1.075928229	-0.235882792	8.250317695	-3.312466897	1.04E-03	1.33E-02
USP36	0.145864548	-0.235730844	10.35764886	-3.687124175	2.68E-04	5.31E-03
KIF1B	-2.40753724	-0.235683442	10.9735336	-2.852983245	4.63E-03	3.75E-02
C16orf48	-2.23792664	-0.235484416	7.682930519	-2.915286673	3.81E-03	3.29E-02
TSC1	-0.078536245	-0.235401623	9.365876461	-3.620989066	3.43E-04	6.28E-03
POU5F2	-1.571232516	-0.235388312	0.735400649	-3.148911522	1.80E-03	1.95E-02
SELO	-1.627091106	-0.235166883	8.463036039	-3.129966532	1.92E-03	2.03E-02
ZNF252	-1.799402829	-0.235158117	9.959494968	-3.070836335	2.33E-03	2.33E-02
HMBOX1	-2.729198681	-0.235124351	6.653079383	-2.731138113	6.68E-03	4.82E-02
ANKRD57	-1.109251089	-0.234842208	8.559040584	-3.301700223	1.07E-03	1.36E-02
HGSNAT	0.35871321	-0.23463961	11.16578084	-3.748869594	2.12E-04	4.46E-03
KIF13A	-2.529534803	-0.234582143	9.300088149	-2.807361362	5.31E-03	4.14E-02
C17orf85	0.125274331	-0.234469481	8.950174351	-3.681101096	2.74E-04	5.41E-03
WASH2P	-2.330716898	-0.234363312	8.025678084	-2.881359639	4.24E-03	3.55E-02
RBM6	-0.256033921	-0.234185065	9.863494481	-3.56788371	4.17E-04	7.17E-03
CHD1	-0.7273719	-0.234081494	8.755557955	-3.423201387	7.03E-04	1.02E-02
TSC2	-1.653757996	-0.233908442	11.60078149	-3.120884244	1.97E-03	2.07E-02
INO80D	-0.039825936	-0.233464286	8.770813961	-3.632476256	3.29E-04	6.11E-03
ZNF641	-1.034464048	-0.233393831	7.823518019	-3.325818839	9.89E-04	1.29E-02
C17orf103	-2.765397945	-0.233336688	8.420030357	-2.717101314	6.96E-03	4.97E-02
MBTD1	-1.120632671	-0.233221104	9.111695292	-3.298015334	1.09E-03	1.38E-02
THRA	-2.414136823	-0.233178571	9.785523052	-2.850532982	4.66E-03	3.77E-02
RGPD4	0.852691994	-0.232936039	5.955435877	-3.888728896	1.24E-04	3.06E-03
SETD1B	-1.716715924	-0.232916883	10.82382597	-3.099342987	2.12E-03	2.18E-02
DDHD2	-2.188592668	-0.23275	9.432789286	-2.933173739	3.61E-03	3.17E-02
MGA	-0.225122565	-0.232723701	9.9612875	-3.57718401	4.03E-04	6.99E-03
IREB2	1.840630827	-0.232671104	9.937121591	-4.155669338	4.21E-05	1.46E-03
USP22	0.664899809	-0.232423701	11.97678555	-3.836104115	1.52E-04	3.55E-03
CLDN20	-2.617496419	-0.232411039	0.953676299	-2.774026442	5.88E-03	4.42E-02

WDR37	-1.662160642	-0.232337662	8.850080519	-3.118017314	1.99E-03	2.09E-02
EP300	-0.337659431	-0.231836039	11.2467138	-3.543217383	4.57E-04	7.62E-03
KDSR	-0.551846538	-0.231641558	9.974650649	-3.477728614	5.79E-04	8.99E-03
ARID4A	1.583244365	-0.231519805	8.722340747	-4.087629241	5.57E-05	1.72E-03
ZNF605	-2.265187224	-0.231069481	8.738573701	-2.905358184	3.93E-03	3.37E-02
TANC1	-1.092117432	-0.231057468	9.869401786	-3.307240204	1.05E-03	1.35E-02
ZNF638	1.557145949	-0.230824351	10.29974821	-4.080673645	5.73E-05	1.75E-03
SPTBN1	-2.017720695	-0.230639935	11.89998977	-2.994347379	2.97E-03	2.77E-02
SEC14L1	-2.117398806	-0.230525	10.48696218	-2.958806629	3.33E-03	3.01E-02
SCAND2	-1.090295064	-0.230376623	8.109972727	-3.30782894	1.05E-03	1.34E-02
UBL3	-1.449891582	-0.230371429	9.640956494	-3.189702745	1.57E-03	1.78E-02
ZNF251	-1.319484369	-0.230268831	9.389350649	-3.233005625	1.36E-03	1.61E-02
ACVR2A	-1.450726021	-0.230080519	8.299250649	-3.189423896	1.57E-03	1.78E-02
TMOD3	-0.124348004	-0.230018506	10.38719464	-3.607351291	3.61E-04	6.48E-03
ZDHHC17	-0.339230196	-0.229788312	8.479344156	-3.542741169	4.57E-04	7.62E-03
RBM33	2.075934062	-0.229744805	10.12078474	-4.217015516	3.26E-05	1.23E-03
ZBTB6	0.35107003	-0.229485065	8.525027597	-3.746668423	2.14E-04	4.49E-03
PPP3CA	-2.185032235	-0.22884026	9.694119481	-2.934460667	3.59E-03	3.17E-02
BIRC6	0.741746411	-0.228446104	10.62293019	-3.857717209	1.39E-04	3.31E-03
GTF2IRD2P1	-2.650633425	-0.228093831	7.138514773	-2.761369451	6.10E-03	4.54E-02
KCTD7	-2.588489179	-0.228063961	8.001203084	-2.78506119	5.68E-03	4.32E-02
LOC284900	-2.655974897	-0.227720779	7.107466234	-2.759324066	6.14E-03	4.56E-02
RBM5	0.356052439	-0.227655519	10.53514562	-3.748103448	2.13E-04	4.47E-03
TBC1D2B	-1.718556534	-0.227618506	9.635066721	-3.098711105	2.12E-03	2.18E-02
C21orf84	-2.055655056	-0.227601299	0.537275325	-2.980868737	3.10E-03	2.86E-02
SEC16A	-0.873570663	-0.227452273	11.52585114	-3.377164365	8.27E-04	1.15E-02
ZNF324B	-2.35995315	-0.227425974	6.724246753	-2.870591445	4.38E-03	3.62E-02
MTR	-1.636083248	-0.227332143	9.883983929	-3.126906722	1.94E-03	2.05E-02
GOLGA4	-1.225275403	-0.227129221	10.13363994	-3.26395516	1.22E-03	1.49E-02
ZNF623	-1.456233373	-0.226485714	9.70173961	-3.187582904	1.58E-03	1.79E-02
PRDM10	1.27979753	-0.226103247	7.980815909	-4.006089783	7.75E-05	2.19E-03
RABL2B	-2.681442257	-0.225857143	8.67227987	-2.749552091	6.32E-03	4.65E-02
PCMTD1	-1.51823417	-0.225725	10.11116088	-3.166788392	1.70E-03	1.87E-02
PI4KA	-1.425345735	-0.225558766	10.58468166	-3.197895202	1.53E-03	1.75E-02
TGFBRAP1	-0.601860659	-0.225532143	8.404496266	-3.462272383	6.12E-04	9.31E-03
VPS13A	-1.145913666	-0.225267857	8.214058929	-3.289816664	1.12E-03	1.40E-02
HELZ	0.900366282	-0.225161688	10.07160942	-3.901986475	1.17E-04	2.95E-03
ZC3H4	3.954710538	-0.225097078	10.43554172	-4.6813047	4.28E-06	3.15E-04
ZNF160	-1.679641571	-0.225048377	8.887681656	-3.112045006	2.03E-03	2.11E-02
KIAA0195	-1.514769109	-0.225024026	10.96414383	-3.167953906	1.69E-03	1.87E-02
ZNF407	-1.432156161	-0.224905844	7.649280195	-3.195624109	1.54E-03	1.76E-02
ZNF286A	-2.595873503	-0.224863961	8.027668669	-2.782256035	5.73E-03	4.34E-02
OXR1	-1.814998006	-0.224707468	9.85685276	-3.065431966	2.37E-03	2.37E-02
C11orf61	-2.197754778	-0.224647727	7.969487175	-2.929859619	3.65E-03	3.19E-02
ZNF721	-1.498407664	-0.224602922	8.650455682	-3.173451869	1.66E-03	1.85E-02

ZFYVE26	-1.447081907	-0.224564286	9.593947727	-3.190641501	1.57E-03	1.78E-02
TUBGCP4	0.096438894	-0.224183117	8.326321429	-3.672650965	2.83E-04	5.54E-03
JRKL	-2.140336234	-0.223988636	8.339275812	-2.95057109	3.42E-03	3.07E-02
POLI	-1.936522626	-0.223955519	8.047903409	-3.023008071	2.71E-03	2.60E-02
ABL2	0.186770592	-0.223646104	9.198795779	-3.699063518	2.56E-04	5.14E-03
FOXN3	-1.963304272	-0.223598701	10.01015649	-3.013583227	2.80E-03	2.66E-02
RNF19A	-2.656098949	-0.22335974	10.34175519	-2.759276546	6.14E-03	4.56E-02
USPL1	-0.070417536	-0.223138636	8.54620276	-3.623401035	3.40E-04	6.26E-03
DDX42	1.670308996	-0.223100325	11.08389205	-4.110757149	5.07E-05	1.61E-03
KIAA0182	-2.157434385	-0.222686039	10.56405438	-2.944417978	3.48E-03	3.10E-02
ZNF646	-0.065781057	-0.222603896	9.530627273	-3.624777817	3.38E-04	6.24E-03
VPS39	2.068744063	-0.2219	10.46832468	-4.215152749	3.29E-05	1.23E-03
NFRKB	-1.723460274	-0.221737987	9.763333604	-3.097027062	2.14E-03	2.19E-02
SPRED1	-2.260448854	-0.221717532	9.617425	-2.90708623	3.91E-03	3.36E-02
RGMB	-2.419214576	-0.221488636	9.136732305	-2.848646379	4.69E-03	3.78E-02
ZBED4	-0.797057396	-0.221282143	9.145304383	-3.401329776	7.59E-04	1.08E-02
USP46	0.322256654	-0.221107792	8.852782468	-3.738359816	2.21E-04	4.60E-03
MPRIP	-0.397298046	-0.221083766	10.15788994	-3.52509505	4.88E-04	7.97E-03
AFF1	-2.366150446	-0.220963961	10.16421932	-2.868303967	4.41E-03	3.63E-02
FHOD1	-0.559548304	-0.220847078	8.737708929	-3.475352609	5.84E-04	9.04E-03
CLMN	-2.025535561	-0.220534416	9.81044513	-2.991575318	3.00E-03	2.78E-02
GRAMD4	-1.856650642	-0.220477597	9.109324838	-3.050953573	2.48E-03	2.45E-02
BBS1	0.23297656	-0.219905844	10.21130422	-3.712507643	2.44E-04	4.93E-03
ZBTB41	-0.079764849	-0.219167532	8.872537662	-3.620623935	3.44E-04	6.28E-03
CBL	-1.886659139	-0.218863312	9.799834578	-3.040482548	2.57E-03	2.51E-02
MED23	-0.532756202	-0.218697727	9.302181006	-3.483611568	5.67E-04	8.87E-03
AGPAT6	-0.921234314	-0.218612338	10.17339123	-3.362029356	8.72E-04	1.19E-02
LATS1	-1.860740331	-0.218522403	8.143310227	-3.049528524	2.49E-03	2.45E-02
ZNF548	-2.148113752	-0.218494805	7.874111039	-2.947773696	3.45E-03	3.08E-02
DSTYK	-1.476542701	-0.218084091	9.322767695	-3.180785324	1.62E-03	1.82E-02
BTBD12	-2.374688428	-0.217931494	7.909055032	-2.865149692	4.46E-03	3.64E-02
RC3H1	3.599002316	-0.217876623	9.602204545	-4.596549449	6.28E-06	4.05E-04
TK2	-1.161361644	-0.21775487	8.671762825	-3.284797496	1.14E-03	1.42E-02
ZNF197	-0.019714175	-0.217591558	8.283236688	-3.638431272	3.22E-04	6.04E-03
TBC1D13	-0.570001389	-0.21736039	9.59058539	-3.472125421	5.90E-04	9.11E-03
C9orf5	-0.309331772	-0.217223701	10.88426023	-3.55179551	4.43E-04	7.49E-03
ZNF805	-1.610354574	-0.216740584	8.214749838	-3.135654129	1.88E-03	2.01E-02
EIF3L	-1.993945677	-0.216703571	12.50336769	-3.002765956	2.90E-03	2.72E-02
MTMR9	-1.566077832	-0.216479545	7.665890747	-3.150654412	1.79E-03	1.94E-02
MAP4	-0.456763148	-0.216436039	12.58352873	-3.506939738	5.21E-04	8.36E-03
ZNF292	-1.862405388	-0.216379221	9.354970779	-3.048948157	2.50E-03	2.46E-02
ZHX1	-2.523468513	-0.216176299	9.757713149	-2.809646479	5.28E-03	4.12E-02
CLK4	-2.215537423	-0.216068831	7.955546753	-2.923417158	3.72E-03	3.23E-02
CEP135	-2.004392316	-0.21602987	7.939850649	-2.99906961	2.93E-03	2.74E-02
ZNF500	-1.464342796	-0.215675	8.375127435	-3.184870286	1.60E-03	1.80E-02

TPCN2	-1.550234881	-0.21558474	8.120606331	-3.156005558	1.76E-03	1.92E-02
UBR3	2.002991871	-0.215575974	9.393980195	-4.198083838	3.53E-05	1.29E-03
RELL1	-2.43125844	-0.215497078	8.122067695	-2.844166829	4.75E-03	3.82E-02
POGZ	-1.724426809	-0.215351299	11.30899708	-3.096695033	2.14E-03	2.19E-02
APC	-1.511311249	-0.215261364	8.701838799	-3.169116599	1.68E-03	1.86E-02
TNFRSF10B	-2.550855132	-0.21503539	9.526720617	-2.799316156	5.45E-03	4.20E-02
RNF20	0.919430879	-0.214771429	9.727662987	-3.907276718	1.15E-04	2.91E-03
HAUS3	-0.938097811	-0.214433442	8.340026786	-3.35665942	8.88E-04	1.20E-02
AHI1	-2.638908759	-0.214358117	7.866064448	-2.765854086	6.02E-03	4.50E-02
CEP164	-2.360432969	-0.214270455	9.770442695	-2.870414401	4.38E-03	3.62E-02
SPTAN1	-1.821253207	-0.214123052	12.73514042	-3.063261768	2.38E-03	2.38E-02
CNTROB	-0.552946374	-0.214011039	9.276062013	-3.477389404	5.79E-04	8.99E-03
ATXN1L	-2.005076833	-0.213947078	9.919915422	-2.998827256	2.93E-03	2.74E-02
KIAA1370	-1.182028955	-0.213848377	8.753423864	-3.278071353	1.17E-03	1.44E-02
KLF3	4.344130875	-0.213726299	10.61386282	-4.772601194	2.82E-06	2.32E-04
CP110	-2.010483208	-0.21356526	8.478681656	-2.996912482	2.95E-03	2.75E-02
PCF11	0.173228065	-0.213440584	9.902236201	-3.695114725	2.60E-04	5.19E-03
RASAL2	-1.826930357	-0.213369156	9.172375162	-3.06129087	2.40E-03	2.40E-02
SETX	1.506802363	-0.213062987	10.09018344	-4.067226344	6.05E-05	1.83E-03
NEURL4	-1.971397069	-0.213062662	8.617869968	-3.010729797	2.82E-03	2.67E-02
USP34	-0.808471437	-0.21212987	11.1329974	-3.39773494	7.69E-04	1.09E-02
LUC7L3	-2.184853568	-0.211998701	10.99858506	-2.934525233	3.59E-03	3.17E-02
MGEA5	-1.223569103	-0.211625325	10.93320747	-3.264513194	1.22E-03	1.49E-02
CES2	-1.700539972	-0.211454221	9.410113799	-3.104891007	2.08E-03	2.15E-02
OTUD4	-1.209464969	-0.211288312	10.23448506	-3.269122464	1.20E-03	1.47E-02
KIAA2026	-2.363000185	-0.211283117	9.420317532	-2.869466972	4.40E-03	3.62E-02
ZMYND11	-0.784824243	-0.211128247	10.73921445	-3.405178693	7.49E-04	1.07E-02
RAPH1	-2.502460794	-0.210956818	8.640068994	-2.817546314	5.15E-03	4.05E-02
ZER1	1.042807821	-0.210798377	10.8042599	-3.941357774	1.00E-04	2.64E-03
CCDC45	-1.598909445	-0.210482143	8.843716071	-3.13953799	1.86E-03	1.99E-02
TLN1	-2.000416804	-0.210041234	13.15689432	-3.000476775	2.92E-03	2.73E-02
DYRK2	-2.610050179	-0.210015909	10.35919724	-2.776863064	5.83E-03	4.39E-02
CYB5D1	-2.062070217	-0.209844805	8.740263961	-2.978583657	3.13E-03	2.88E-02
C5orf41	-2.606950025	-0.209663312	8.207959903	-2.778043247	5.81E-03	4.37E-02
SIK2	-2.067868048	-0.209657468	9.677645292	-2.976517049	3.15E-03	2.89E-02
GOPC	-0.355426989	-0.209622403	9.545813474	-3.537827295	4.66E-04	7.74E-03
FOXP1	-1.089011283	-0.209547727	9.592491071	-3.308243621	1.05E-03	1.34E-02
C1orf107	-0.322243328	-0.208987013	9.922221429	-3.547888021	4.49E-04	7.56E-03
TECPR1	-2.570863495	-0.208937662	8.090132468	-2.791746018	5.57E-03	4.27E-02
MBD5	-1.437222255	-0.208907143	8.602407468	-3.193933727	1.55E-03	1.77E-02
ALKBH8	-2.44440641	-0.208875649	6.807938149	-2.839268982	4.82E-03	3.86E-02
PLDN	0.063464867	-0.20871526	9.658748864	-3.662966226	2.94E-04	5.65E-03
DPH1	-1.164994949	-0.208297727	9.663384253	-3.283615971	1.14E-03	1.42E-02
DGCR8	2.548844047	-0.208267208	9.594657955	-4.337978657	1.95E-05	8.81E-04
SECISBP2	1.682917193	-0.208056818	9.204706006	-4.114096779	5.00E-05	1.61E-03

GPR107	-0.232443788	-0.207862987	11.10868539	-3.574983279	4.07E-04	7.02E-03
CHD6	-2.477836001	-0.207805519	10.59631412	-2.826779685	5.01E-03	3.96E-02
DOPEY1	-2.324333595	-0.207733117	8.512058766	-2.883705657	4.21E-03	3.53E-02
SLC12A4	-2.757149303	-0.207719156	9.294050812	-2.720305908	6.89E-03	4.94E-02
TUG1	-0.816372717	-0.207378571	11.89469253	-3.39524438	7.76E-04	1.10E-02
SIDT2	-2.372608501	-0.207213312	10.0794888	-2.865918403	4.45E-03	3.64E-02
TMCO7	-1.91686139	-0.207015909	6.8482375	-3.029909591	2.65E-03	2.57E-02
CEP68	-0.159123097	-0.2066	9.692711039	-3.596967429	3.75E-04	6.67E-03
RFC1	0.455012685	-0.206460714	9.951808604	-3.776503057	1.91E-04	4.15E-03
MYST4	-2.719023397	-0.206365909	10.2865586	-2.735071391	6.60E-03	4.79E-02
SLTM	-0.491307479	-0.206299351	10.23849903	-3.496353169	5.41E-04	8.60E-03
PCMTD2	-2.089078388	-0.206166558	10.36152776	-2.968945181	3.22E-03	2.94E-02
WASH7P	-2.371454352	-0.205983117	10.27183701	-2.866344876	4.44E-03	3.64E-02
TCF12	-1.127657496	-0.205812013	10.53079205	-3.295739073	1.10E-03	1.38E-02
LMTK2	-2.537599983	-0.205766558	9.012855682	-2.804320548	5.36E-03	4.16E-02
PAFAH1B1	-0.28028991	-0.20503539	10.96452386	-3.560570204	4.29E-04	7.32E-03
FBXW7	-0.632006391	-0.204026299	7.684430357	-3.452925415	6.32E-04	9.49E-03
MON2	0.266691502	-0.203617208	9.399468669	-3.722289441	2.35E-04	4.80E-03
MAVS	-1.849631947	-0.203493831	11.74373945	-3.053397776	2.46E-03	2.43E-02
C9orf41	-2.760409282	-0.203354221	7.108473539	-2.719039837	6.92E-03	4.95E-02
C14orf43	-1.19834881	-0.203271104	10.44647354	-3.272750999	1.19E-03	1.46E-02
MEX3C	-1.796771904	-0.203256494	9.92709513	-3.071747179	2.32E-03	2.33E-02
MAFG	-1.773585581	-0.202841234	9.0175625	-3.079763509	2.26E-03	2.29E-02
SETD2	-0.056572003	-0.202786688	10.61794367	-3.627510986	3.35E-04	6.20E-03
DGKD	-2.702330197	-0.202450325	8.653474188	-2.741512562	6.47E-03	4.73E-02
FTHL3	-2.573060724	-0.202443506	3.708317208	-2.790913512	5.58E-03	4.28E-02
RBM19	-2.119972289	-0.202175325	9.078215584	-2.957883712	3.34E-03	3.02E-02
MRRF	-2.270285195	-0.201829545	8.986919643	-2.90349791	3.96E-03	3.38E-02
ZNF510	-0.509376476	-0.201174351	7.956903734	-3.490803911	5.52E-04	8.72E-03
NUFIP2	0.481161609	-0.200867857	10.66894334	-3.783974918	1.86E-04	4.06E-03
CASC4	-1.503761472	-0.20078961	11.12141688	-3.1716538	1.67E-03	1.85E-02
BAHD1	-1.564806657	-0.200427273	9.801078571	-3.15108408	1.79E-03	1.94E-02
DDX17	3.016031762	-0.200226948	13.0541112	-4.454629608	1.18E-05	6.22E-04
OR4F29	-2.390067382	-0.200077273	0.56300487	-2.859459779	4.53E-03	3.70E-02
TCF20	-1.923596445	-0.199665909	10.20770666	-3.027547106	2.67E-03	2.58E-02
NCKIPSD	-0.360480513	-0.199665584	9.923012013	-3.536292844	4.68E-04	7.77E-03
POU4F3	-2.272744801	-0.199654221	0.824343019	-2.902599985	3.97E-03	3.38E-02
CSNK1G1	-0.190971697	-0.199551948	9.711618831	-3.587433261	3.88E-04	6.83E-03
DDX51	-2.293043954	-0.199478247	8.875446591	-2.895179354	4.06E-03	3.44E-02
MINK1	-1.270604849	-0.199222727	11.09165942	-3.2490978	1.29E-03	1.54E-02
POLR1A	-1.724285485	-0.198973377	9.974090584	-3.096743583	2.14E-03	2.19E-02
WHSC1L1	-2.37248866	-0.198827273	9.835982468	-2.865962689	4.44E-03	3.64E-02
BCL2L2	-2.499831406	-0.198825325	9.241768506	-2.818533603	5.14E-03	4.04E-02
ZNF189	-1.530736185	-0.198497403	8.451892208	-3.162579853	1.72E-03	1.89E-02
ZFP90	-1.81237343	-0.198458117	8.687911201	-3.066342115	2.36E-03	2.36E-02

PSKH1	-1.581020524	-0.198113636	9.641373052	-3.145599538	1.82E-03	1.96E-02
OXSR1	-0.376527815	-0.198049351	10.12179675	-3.531416183	4.77E-04	7.88E-03
ARHGEF12	-2.71276218	-0.19784026	11.52362727	-2.737489008	6.55E-03	4.76E-02
MAPK1	-1.796262868	-0.197794156	11.21254253	-3.071923382	2.32E-03	2.33E-02
ZNF624	-0.980126206	-0.197512013	6.06794724	-3.343241302	9.31E-04	1.24E-02
KLHDC10	-2.704126613	-0.197171104	9.250935227	-2.740820095	6.49E-03	4.73E-02
TNKS2	-0.652589117	-0.197118182	10.36477727	-3.446530112	6.47E-04	9.65E-03
ARHGAP5	-1.351223982	-0.19685487	10.71177386	-3.222516182	1.41E-03	1.66E-02
RLIM	0.645083438	-0.196600325	10.24445763	-3.830512866	1.55E-04	3.59E-03
GLTSCR1	-2.373903598	-0.196337013	9.582450974	-2.865439777	4.45E-03	3.64E-02
CALCOCO1	-2.234916813	-0.196294805	10.73866494	-2.916380916	3.80E-03	3.28E-02
TSEN2	-1.926739426	-0.196239935	7.880953409	-3.026444036	2.68E-03	2.59E-02
STAG2	-1.974216775	-0.195575974	11.09086396	-3.009735003	2.83E-03	2.68E-02
USP24	-1.410572451	-0.195341558	10.40069805	-3.202816524	1.50E-03	1.74E-02
CORO7	-2.656338661	-0.195267857	9.173059578	-2.759184719	6.14E-03	4.56E-02
WDR7	-2.234807049	-0.195065909	8.231951461	-2.916420814	3.80E-03	3.28E-02
ADD1	-0.560188204	-0.194951948	12.05486169	-3.475155131	5.84E-04	9.04E-03
MED1	-1.192239521	-0.194867208	9.956974838	-3.274743599	1.18E-03	1.45E-02
FRS2	-2.399188797	-0.194227273	10.04521039	-2.856079965	4.58E-03	3.72E-02
C4orf23	-2.296668492	-0.194068182	7.902366558	-2.893852462	4.08E-03	3.45E-02
TAB1	-0.395995361	-0.194043506	9.085145779	-3.525491811	4.87E-04	7.97E-03
CCDC142	-1.164013057	-0.194031169	7.95862013	-3.283935315	1.14E-03	1.42E-02
SP1	0.363522712	-0.193875649	11.61660633	-3.750254087	2.11E-04	4.45E-03
TELO2	-1.415031532	-0.193851623	9.315557305	-3.201331845	1.51E-03	1.74E-02
CCDC25	-2.367007721	-0.193744156	9.327179221	-2.867987404	4.42E-03	3.63E-02
AEBP2	-2.341077871	-0.193485065	9.840616558	-2.877547881	4.29E-03	3.57E-02
ZDHHC8	-2.553721114	-0.193388636	10.0676401	-2.798233007	5.46E-03	4.22E-02
RBL2	-1.011406584	-0.193222078	10.48610584	-3.333222124	9.63E-04	1.27E-02
PAN3	-2.209087627	-0.192992857	9.526610065	-2.925755397	3.69E-03	3.22E-02
C5orf40	-2.626350127	-0.192721429	0.646917857	-2.770650061	5.94E-03	4.45E-02
CARD8	-1.586971945	-0.192685065	8.449127597	-3.143584151	1.83E-03	1.97E-02
RAB14	0.220995684	-0.19261526	10.91945081	-3.709025943	2.47E-04	4.98E-03
ANAPC1	-1.719676805	-0.192572403	9.812896266	-3.098326457	2.13E-03	2.18E-02
TMEM170A	-2.27462051	-0.192492532	8.766166071	-2.901915046	3.98E-03	3.39E-02
AKAP11	-1.606385849	-0.192395779	9.497404058	-3.137001411	1.87E-03	2.00E-02
EHMT1	-1.879771738	-0.192122727	10.37498344	-3.042888798	2.55E-03	2.49E-02
SECISBP2L	-1.73301002	-0.191853896	9.763739935	-3.093745019	2.16E-03	2.20E-02
ALAD	-1.438027424	-0.191731494	9.705650487	-3.193664992	1.55E-03	1.77E-02
CXorf23	-1.265928295	-0.191723701	6.908271266	-3.250633531	1.28E-03	1.54E-02
MKL2	-2.426446393	-0.191402273	9.906881981	-2.845957405	4.73E-03	3.80E-02
FNBP4	-2.063292875	-0.191107792	10.22719416	-2.978147959	3.13E-03	2.88E-02
DENND5A	-2.171313278	-0.190982468	10.03118344	-2.939414448	3.54E-03	3.14E-02
RALGDS	-1.857430547	-0.190871104	10.75779627	-3.050681864	2.48E-03	2.45E-02
BTBD7	-1.885045944	-0.190760714	9.795864448	-3.04104631	2.56E-03	2.50E-02
SLC9A8	-2.356712701	-0.190152273	9.543646591	-2.871786837	4.37E-03	3.62E-02

TAPT1	-2.364672871	-0.190042857	8.603454545	-2.86884951	4.41E-03	3.63E-02
ASH2L	-2.504528681	-0.190033117	9.840999026	-2.816769629	5.17E-03	4.05E-02
ZMYM2	-2.052370352	-0.189902922	10.14542841	-2.98203811	3.09E-03	2.85E-02
TGFBR1	-1.516744454	-0.189650974	9.493058279	-3.167289525	1.69E-03	1.87E-02
BCLAF1	1.66857859	-0.189488312	11.27814805	-4.110298615	5.08E-05	1.61E-03
CCDC76	-2.142429867	-0.188871429	8.188345455	-2.949818304	3.42E-03	3.07E-02
RICTOR	-1.443450301	-0.188261688	9.081650325	-3.191854495	1.56E-03	1.78E-02
KIAA1143	-0.600331294	-0.188067532	9.643843506	-3.462745957	6.11E-04	9.31E-03
VHL	-1.932310549	-0.188012013	9.779340097	-3.024487848	2.70E-03	2.60E-02
HDAC5	-2.532484433	-0.187980519	10.21012013	-2.806249626	5.33E-03	4.15E-02
VGLL4	-0.985705474	-0.187411688	11.65453571	-3.341456292	9.36E-04	1.24E-02
KIAA0831	-1.609168969	-0.187380844	8.747825162	-3.136056669	1.88E-03	2.00E-02
CHIC1	-1.805720156	-0.187168182	8.184069156	-3.068648199	2.34E-03	2.35E-02
ZC3H11A	-1.128590945	-0.186993831	11.3119849	-3.295436496	1.10E-03	1.38E-02
ZNF23	-1.535691881	-0.186861688	7.097759416	-3.160910173	1.73E-03	1.89E-02
LOC100130264	-2.483447342	-0.186812987	0.426835065	-2.824678159	5.04E-03	3.97E-02
RNF111	-1.521474094	-0.186651948	9.461505195	-3.165698242	1.70E-03	1.87E-02
ARAP1	-2.493291448	-0.186637013	11.33144383	-2.820987821	5.10E-03	4.01E-02
PUM1	1.67609	-0.186611688	11.73615065	-4.112288708	5.03E-05	1.61E-03
RIF1	-2.100513493	-0.186548052	9.172983117	-2.964855411	3.27E-03	2.97E-02
FAM193A	-1.865812242	-0.186474675	10.1216276	-3.047760351	2.51E-03	2.46E-02
CNOT1	-1.789648419	-0.186427273	12.00556623	-3.074212106	2.30E-03	2.32E-02
CNNM3	-1.386638025	-0.185997078	10.24701769	-3.210774692	1.46E-03	1.71E-02
RUFY2	-1.080603985	-0.185272403	8.164329383	-3.310958114	1.04E-03	1.33E-02
XRN1	-1.844085944	-0.185165909	9.513750162	-3.055327828	2.45E-03	2.43E-02
SPAG9	-2.381217272	-0.185100974	9.661080682	-2.862735454	4.49E-03	3.67E-02
XPO4	-2.041357384	-0.184635065	9.120912338	-2.98595564	3.05E-03	2.82E-02
CSNK1E	-1.604180773	-0.184539286	10.85080244	-3.137749744	1.87E-03	2.00E-02
CLIP1	-2.224461496	-0.184466883	9.880796429	-2.920179006	3.76E-03	3.26E-02
SMARCC2	-1.038152144	-0.184376948	11.79416023	-3.324633247	9.93E-04	1.29E-02
ERCC6	-2.590377289	-0.184221429	8.080163961	-2.784344191	5.70E-03	4.32E-02
BRD3	-2.195494008	-0.183435714	10.33921461	-2.930677714	3.64E-03	3.19E-02
KHNYN	-2.467591652	-0.183403896	10.52319805	-2.830612522	4.95E-03	3.93E-02
FBXO31	-0.880917791	-0.183181169	8.712067208	-3.374835474	8.33E-04	1.15E-02
ATP6V1C1	-2.540235931	-0.182948377	10.59272711	-2.803326039	5.38E-03	4.17E-02
PRKAR1A	0.152266708	-0.182331818	11.81175487	-3.688995114	2.66E-04	5.28E-03
C15orf17	-2.339177761	-0.182239935	9.551181981	-2.878247281	4.28E-03	3.57E-02
PHF3	-2.133928321	-0.181898052	10.449125	-2.95287399	3.39E-03	3.05E-02
C17orf66	-2.457849298	-0.181869481	0.482205519	-2.834252986	4.90E-03	3.90E-02
ROCK1	-1.401328917	-0.181823052	9.910304058	-3.205892172	1.49E-03	1.73E-02
SCRN3	-2.771674064	-0.181111364	7.676081331	-2.714660633	7.01E-03	5.00E-02
SURF6	-1.539889229	-0.181098052	9.075975	-3.159495352	1.74E-03	1.90E-02
XIAP	-0.709808097	-0.180982468	10.00085292	-3.428693642	6.89E-04	1.01E-02
ETAA1	-2.341668492	-0.180664286	8.40239513	-2.877330451	4.29E-03	3.58E-02
NCRNA00161	-1.719278487	-0.180606494	0.395081169	-3.098463226	2.13E-03	2.18E-02

HDAC7	-2.263268652	-0.180482792	12.05878458	-2.906057989	3.93E-03	3.36E-02
ANO6	-2.492067171	-0.179924026	10.44293409	-2.821447024	5.09E-03	4.01E-02
SS18L1	-2.634362637	-0.179854545	8.882699351	-2.767591101	5.99E-03	4.49E-02
GGT8P	-2.516753959	-0.179837013	0.431794481	-2.812173737	5.24E-03	4.09E-02
CAMTA2	-2.415730913	-0.179083117	9.861621429	-2.849940836	4.67E-03	3.77E-02
ZNF740	-1.330691551	-0.178849351	9.605848052	-3.229305455	1.38E-03	1.63E-02
ZNF652	-1.964195617	-0.17866039	10.01689513	-3.013269073	2.80E-03	2.66E-02
MGC15885	-2.187761863	-0.178628571	0.41663961	-2.933474084	3.60E-03	3.17E-02
TATDN2	-0.518915659	-0.178414286	10.5025539	-3.487871007	5.58E-04	8.76E-03
CEP350	-1.840840125	-0.178058117	9.475424188	-3.056456867	2.44E-03	2.42E-02
MAT2A	-0.654898463	-0.178008117	12.2268875	-3.445811887	6.49E-04	9.66E-03
HS2ST1	-0.888835556	-0.177932468	9.89557013	-3.372324034	8.41E-04	1.16E-02
PDS5A	0.012224545	-0.177748701	11.09593539	-3.647869933	3.11E-04	5.88E-03
RALGAPA1	-2.534894777	-0.177419481	8.17175	-2.80534084	5.35E-03	4.16E-02
SEPT10	-2.168584556	-0.177311364	9.899415747	-2.940398823	3.53E-03	3.13E-02
HP1BP3	-1.92221759	-0.177237987	12.00734854	-3.028030914	2.67E-03	2.58E-02
KDM3B	-1.641567484	-0.177152273	11.10347062	-3.125039191	1.95E-03	2.05E-02
UBXN2B	-2.030232564	-0.176815584	10.04929221	-2.98990805	3.02E-03	2.79E-02
POLDIP3	-0.787659648	-0.176241558	10.62085455	-3.404286948	7.51E-04	1.07E-02
MED15	-2.121487505	-0.176221429	10.92563539	-2.957340191	3.34E-03	3.02E-02
BAT2L2	-1.672530955	-0.175966234	12.13337208	-3.114475621	2.02E-03	2.10E-02
SON	-1.128221012	-0.175764935	12.10195714	-3.295556413	1.10E-03	1.38E-02
PARP16	-2.683661905	-0.175335714	8.069366558	-2.748698835	6.34E-03	4.66E-02
ZNF263	-1.066245208	-0.175317532	9.170338636	-3.315589409	1.02E-03	1.32E-02
HIRA	-2.759152549	-0.175235065	9.647278571	-2.719527978	6.91E-03	4.95E-02
LDOC1L	-2.739932743	-0.175206818	10.10571347	-2.72698299	6.76E-03	4.86E-02
MED12	-2.719445365	-0.17481526	10.8040164	-2.734908386	6.60E-03	4.79E-02
KCTD3	-2.095449716	-0.174714935	9.917902273	-2.966667128	3.25E-03	2.95E-02
TMEM87B	-2.017431227	-0.174714286	9.94794026	-2.994450012	2.97E-03	2.77E-02
DNAJC5	-2.590305096	-0.174156169	10.96856412	-2.784371609	5.70E-03	4.32E-02
EDEM1	-2.539343403	-0.173285714	9.567022078	-2.803662815	5.37E-03	4.17E-02
SERINC1	-2.38764595	-0.173092208	11.15873247	-2.860356372	4.52E-03	3.69E-02
MGRN1	-2.636910053	-0.173086039	11.28409399	-2.766617894	6.01E-03	4.50E-02
KIAA1310	-2.41690453	-0.172975	10.61256964	-2.849504807	4.67E-03	3.77E-02
CAMSAP1L1	-2.724403782	-0.172934416	9.86748474	-2.732992263	6.64E-03	4.80E-02
CYCSP52	-0.984232803	-0.171970455	0.395530357	-3.341927539	9.35E-04	1.24E-02
NBR1	-1.66439342	-0.171930195	10.89738555	-3.117255089	2.00E-03	2.09E-02
GPATCH8	-0.936617176	-0.171412987	10.45949026	-3.357131226	8.87E-04	1.20E-02
GCFC1	-2.125566325	-0.171332468	9.005499351	-2.955876613	3.36E-03	3.03E-02
SLMAP	-0.963219631	-0.171150649	9.612798052	-3.348644942	9.13E-04	1.22E-02
GGA3	-2.56857678	-0.17111526	10.20847159	-2.792612179	5.56E-03	4.27E-02
ASB1	-2.71313369	-0.171073052	8.65006737	-2.737345615	6.55E-03	4.76E-02
SYNRG	-2.415625872	-0.170897078	9.39673263	-2.849979859	4.67E-03	3.77E-02
C16orf52	-0.848111864	-0.170778247	7.898601786	-3.385222815	8.04E-04	1.12E-02
KIAA1267	-1.499606698	-0.170043831	10.29488263	-3.173049258	1.66E-03	1.85E-02

MTMR1	-2.623573077	-0.169867532	9.652858442	-2.771709515	5.92E-03	4.44E-02
MPHOSPH8	-2.297405234	-0.169603896	9.548898701	-2.893582681	4.08E-03	3.45E-02
XPO7	-1.681279345	-0.169596429	10.74755958	-3.111484915	2.04E-03	2.11E-02
PTPN23	-2.549546313	-0.168658442	11.58131818	-2.799810669	5.44E-03	4.20E-02
APPL1	-1.937852601	-0.168554221	10.3045086	-3.022540686	2.72E-03	2.61E-02
EAF1	-0.959207759	-0.168247403	9.325154221	-3.34992602	9.09E-04	1.22E-02
POLR1B	-2.079895148	-0.167726623	9.754139935	-2.972225732	3.19E-03	2.92E-02
REV1	-0.78542961	-0.167201948	9.12161526	-3.404988321	7.50E-04	1.07E-02
CDK12	-1.673863013	-0.166742208	9.985782143	-3.11402042	2.02E-03	2.10E-02
PITPNB	-2.640805943	-0.166586364	10.24315812	-2.76512889	6.03E-03	4.51E-02
ARIH2	-1.302746503	-0.166331818	10.35529968	-3.238524473	1.33E-03	1.59E-02
KDM2A	-2.454268323	-0.166278896	12.17828718	-2.83558999	4.88E-03	3.88E-02
BNIP2	-2.120548039	-0.166133442	9.316267695	-2.957677197	3.34E-03	3.02E-02
DCTN1	-2.305963084	-0.165983442	11.97584205	-2.890447215	4.12E-03	3.48E-02
TOB2	-1.791020713	-0.165868506	11.00908198	-3.073737398	2.30E-03	2.32E-02
PRO1768	-1.827788854	-0.165214935	0.351819805	-3.060992728	2.40E-03	2.40E-02
BRPF1	-1.514963442	-0.165200649	9.487341883	-3.167888551	1.69E-03	1.87E-02
NUP214	-2.141845945	-0.164965584	10.90361591	-2.950028277	3.42E-03	3.07E-02
AMMECR1L	-1.367349762	-0.164901623	9.179888799	-3.217174652	1.43E-03	1.68E-02
BTBD2	-2.726616848	-0.164761364	11.71210244	-2.732136636	6.66E-03	4.81E-02
SPIN1	-2.348182569	-0.164387987	11.61724302	-2.874931331	4.32E-03	3.59E-02
PRPF4B	-2.353155728	-0.164312013	10.61377386	-2.873098453	4.35E-03	3.61E-02
RAB3GAP1	-2.178980106	-0.163874351	9.911275162	-2.936647003	3.57E-03	3.15E-02
C3orf63	-1.176954253	-0.163701948	10.61333344	-3.279724095	1.16E-03	1.43E-02
CELF1	-2.138226373	-0.163351948	11.20091429	-2.951329527	3.41E-03	3.06E-02
RBPJ	-1.339494934	-0.162948377	9.303421266	-3.226396151	1.39E-03	1.64E-02
KCTD2	-1.607445027	-0.162427597	9.968749188	-3.1366419	1.87E-03	2.00E-02
STX17	-2.509621869	-0.161482792	9.423744318	-2.814855793	5.20E-03	4.07E-02
ADAM10	-2.390523918	-0.160547078	10.3969914	-2.859290706	4.54E-03	3.70E-02
PPP1R12A	-1.943135522	-0.160192532	10.05396477	-3.02068347	2.73E-03	2.62E-02
FUBP3	-0.455527182	-0.159883442	9.970959578	-3.507317971	5.20E-04	8.36E-03
ZNF79	-1.963463027	-0.159786688	6.631375487	-3.013527276	2.80E-03	2.66E-02
KLHL28	-2.442993526	-0.15918539	8.020374188	-2.83979569	4.82E-03	3.86E-02
PDCD6IP	-0.9036913	-0.158704545	11.52480812	-3.367607253	8.55E-04	1.17E-02
RBBP6	-0.898266771	-0.157564286	10.31184643	-3.36933028	8.50E-04	1.17E-02
ZC3H7A	-2.435191748	-0.155975	9.405665747	-2.842702442	4.77E-03	3.83E-02
GRINL1A	-2.45179166	-0.155853571	10.15790568	-2.836514335	4.86E-03	3.87E-02
RNF214	-2.715769934	-0.154881169	8.660771104	-2.73632789	6.57E-03	4.77E-02
VEZF1	-2.548020066	-0.154763312	10.81484821	-2.800387229	5.43E-03	4.20E-02
LEMD3	-2.750044294	-0.154055519	9.299107305	-2.723063325	6.84E-03	4.91E-02
CEP63	-2.479479911	-0.153642857	8.605243506	-2.826164171	5.02E-03	3.96E-02
C3orf19	-1.521063662	-0.15346461	8.538695617	-3.165836361	1.70E-03	1.87E-02
SIN3A	-2.154555987	-0.153023701	11.16780503	-2.945454675	3.47E-03	3.09E-02
EIF4ENIF1	-2.652095153	-0.152973377	9.216625649	-2.760809862	6.11E-03	4.55E-02
PUM2	-2.269689223	-0.152892532	11.34630893	-2.903715441	3.95E-03	3.38E-02

ZYG11B	-2.598535778	-0.152529221	9.695621753	-2.781244032	5.75E-03	4.35E-02
CIZ1	-2.589980126	-0.151073701	11.43637614	-2.784495026	5.69E-03	4.32E-02
MORC3	-2.424037378	-0.151011364	9.055609253	-2.846853405	4.71E-03	3.80E-02
GGA2	-2.105448354	-0.150766234	10.44886883	-2.963088812	3.28E-03	2.98E-02
FEZ2	-2.499960411	-0.149557468	9.201743994	-2.818485171	5.14E-03	4.04E-02
GIT2	-2.557856232	-0.144135065	9.826430519	-2.79666951	5.49E-03	4.23E-02
ARPP19	-2.596588341	-0.143132143	11.47573458	-2.78198434	5.74E-03	4.34E-02
ZXDC	-1.834676465	-0.14295974	10.27983312	-3.058599783	2.42E-03	2.41E-02
C17orf39	-2.472835351	-0.140896429	8.171956331	-2.82865125	4.98E-03	3.94E-02
SNRK	-1.809796024	-0.140330844	9.044454058	-3.06723566	2.35E-03	2.36E-02
RAB5A	-1.644911891	-0.139413636	10.37050422	-3.123899817	1.95E-03	2.06E-02
GOSR1	-1.918460637	-0.137493506	9.770957792	-3.029348773	2.66E-03	2.57E-02
PPP1R10	-2.569795974	-0.134058117	10.63809302	-2.792150404	5.56E-03	4.27E-02
ZBTB11	-1.288043243	-0.130719805	9.345624513	-3.243365246	1.31E-03	1.57E-02
CTCF	-2.460778717	-0.114725	10.40530893	-2.833158804	4.91E-03	3.91E-02
YTHDC1	-2.28483887	-0.106495779	10.40872354	-2.898180995	4.02E-03	3.42E-02
IL4	-2.762353983	-0.082563636	0.107215584	-2.71828431	6.93E-03	4.96E-02
C17orf105	-2.283342737	-0.05092987	0.037681169	-2.898728005	4.02E-03	3.41E-02
SFRS7	-1.940742877	0.119678247	11.24621315	3.021524739	2.73E-03	2.61E-02
TPM3	-2.711505521	0.124333766	13.05996948	2.737973992	6.54E-03	4.76E-02
SAR1A	-2.62677399	0.12834026	10.74668442	2.770488322	5.94E-03	4.45E-02
GORASP2	-2.467587567	0.130933766	11.92969416	2.830614049	4.95E-03	3.93E-02
ADIPOR1	-2.686965807	0.131529221	11.99071396	2.747428314	6.36E-03	4.67E-02
CSTF1	-2.679049557	0.131587013	10.13916558	2.750471588	6.30E-03	4.64E-02
COPB1	-2.668607704	0.13274513	11.37366347	2.754480901	6.23E-03	4.60E-02
RBM4	-0.718277504	0.13403539	11.39649886	3.426046248	6.96E-04	1.02E-02
RNF114	-2.177097628	0.135319481	11.16043961	2.937326737	3.56E-03	3.15E-02
SF3B4	-2.474111103	0.135911039	11.78335942	2.828173893	4.99E-03	3.95E-02
PRPF19	-2.321920077	0.136643182	11.32890211	2.884592213	4.20E-03	3.52E-02
GANAB	-2.086868013	0.136673701	13.53766477	2.969735111	3.22E-03	2.93E-02
RAB1A	-0.427270082	0.137688636	11.82782549	3.515955057	5.04E-04	8.17E-03
PPP1R8	-1.978603682	0.138738961	10.05732208	3.008186683	2.85E-03	2.69E-02
DAP3	-2.110253424	0.139522727	10.88210747	2.961367721	3.30E-03	3.00E-02
SNRPA	-1.862532305	0.140779545	10.94152808	3.048903915	2.50E-03	2.46E-02
DPF2	-1.837491181	0.141346753	10.79708961	3.057621368	2.43E-03	2.42E-02
EIF2S2	-2.211390123	0.143480519	10.48375779	2.924920878	3.70E-03	3.22E-02
ECD	-2.513900248	0.143786039	9.547645617	2.813247179	5.22E-03	4.08E-02
RALY	-2.37834737	0.144015909	11.97049756	2.863796924	4.47E-03	3.66E-02
UNC50	-2.426772105	0.144575325	9.580385714	2.84583624	4.73E-03	3.80E-02
TIPRL	-2.25293729	0.145026623	10.05278929	2.909823652	3.88E-03	3.34E-02
SMARCAL1	-1.71733993	0.145102273	9.350569318	3.099128779	2.12E-03	2.18E-02
YIPF5	-2.536126743	0.145225325	10.06624708	2.804876237	5.35E-03	4.16E-02
C2orf24	-2.76545381	0.146472727	10.62214545	2.717079598	6.96E-03	4.97E-02
CCDC86	-1.987106572	0.147517857	9.720010877	3.005183526	2.87E-03	2.71E-02
WDR46	-2.670161052	0.147717857	10.00524399	2.75388482	6.24E-03	4.61E-02

TPRA1	-2.115946184	0.14861461	9.979565097	2.959327456	3.32E-03	3.01E-02
HNRNPL	2.046603543	0.149598377	12.17210568	4.209412049	3.37E-05	1.26E-03
TMEM138	-2.006764516	0.150193831	9.509628734	2.998229654	2.94E-03	2.74E-02
GOLPH3	-2.419616013	0.151033117	11.60996006	2.848497178	4.69E-03	3.78E-02
INO80E	-2.676134838	0.151761364	9.546744318	2.751591298	6.28E-03	4.63E-02
BUB3	-2.339852143	0.152037662	10.91697987	2.877999071	4.28E-03	3.57E-02
C1orf174	-2.688647861	0.152749351	9.271012338	2.746781263	6.37E-03	4.68E-02
PSMC2	-1.517853185	0.152920779	10.50688506	3.16691656	1.70E-03	1.87E-02
ABCF3	-1.911621008	0.153484091	10.40123555	3.031746586	2.64E-03	2.56E-02
HSPA8	-2.407454127	0.15432987	14.72253766	2.85301409	4.62E-03	3.75E-02
TMCO1	-2.574252105	0.154440909	11.11676461	2.790462012	5.59E-03	4.28E-02
ATP5B	-2.732855728	0.154658442	14.16607532	2.729723157	6.70E-03	4.84E-02
ZC3H15	-1.519949333	0.154664286	10.52745292	3.166211328	1.70E-03	1.87E-02
RBM34	-2.321516723	0.154693506	9.305936364	2.884740351	4.19E-03	3.52E-02
C14orf169	-2.364614552	0.155176948	8.133568994	2.86887104	4.40E-03	3.63E-02
MDH1	-2.621307023	0.156852597	11.09774318	2.772573738	5.90E-03	4.43E-02
BZW1	-1.845573356	0.157072078	11.54024448	3.05481031	2.45E-03	2.43E-02
PARK7	-2.651911725	0.157290909	11.49379026	2.760880089	6.11E-03	4.55E-02
WIBG	-2.219783134	0.157864286	9.206075649	2.921876999	3.74E-03	3.24E-02
ELAVL1	-0.064475627	0.158641558	11.15703117	3.625165372	3.38E-04	6.23E-03
SCYL1	-0.392970676	0.159137987	11.30531964	3.526412885	4.85E-04	7.96E-03
GPN1	-2.527276897	0.159273052	10.3146112	2.808212101	5.30E-03	4.13E-02
VIPAR	-2.359457046	0.159382143	9.197915097	2.870774487	4.38E-03	3.62E-02
HPS6	-2.128313452	0.159382468	9.045498377	2.954890493	3.37E-03	3.04E-02
PPME1	-2.741206325	0.15938474	10.58335795	2.726489591	6.77E-03	4.87E-02
TMEM110	-2.451325295	0.159446429	7.715370617	2.836688361	4.86E-03	3.87E-02
SARNP	-2.53614701	0.159571429	9.522637662	2.804868593	5.35E-03	4.16E-02
GDI2	-2.742330014	0.159736688	12.58773328	2.726054191	6.78E-03	4.87E-02
ALKBH1	-2.1142832	0.160158117	8.171773864	2.959923601	3.32E-03	3.01E-02
H2AFY	-2.665799503	0.160270455	12.04916899	2.755558208	6.21E-03	4.59E-02
PRCC	-0.159289799	0.160302597	11.28494416	3.596917586	3.75E-04	6.67E-03
WDR74	-2.583428383	0.160942857	9.934154545	2.786982137	5.65E-03	4.31E-02
YIPF6	-2.708600126	0.161091234	8.261275487	2.739094961	6.52E-03	4.75E-02
SUMO1	-1.729705942	0.161319481	10.7735013	3.094880929	2.15E-03	2.20E-02
MARS	-2.337568859	0.161372078	11.05930487	2.878839366	4.27E-03	3.57E-02
YIPF3	-1.793731607	0.161949026	12.12591153	3.072799435	2.31E-03	2.32E-02
TP53RK	-1.891579521	0.162145779	9.176701461	3.038762418	2.58E-03	2.51E-02
MMADHC	-1.236168079	0.162252273	10.86760601	3.260390686	1.24E-03	1.50E-02
FDPS	-2.464770102	0.162530195	11.4434138	2.831667317	4.94E-03	3.92E-02
C11orf58	-1.596874274	0.162631494	11.36222289	3.140228147	1.85E-03	1.99E-02
SFRS9	-1.796642775	0.162740584	11.41668912	3.071791878	2.32E-03	2.33E-02
WDR70	-1.615192856	0.162897403	9.185621429	3.134010922	1.89E-03	2.01E-02
RCE1	-2.18915004	0.163080519	9.443328571	2.932972228	3.61E-03	3.17E-02
C12orf44	-2.471558555	0.163345455	10.06464221	2.829128922	4.97E-03	3.94E-02
CREG1	-2.579489219	0.163626299	12.02882484	2.788476472	5.63E-03	4.30E-02

LOC729020	-2.759579023	0.164049351	5.133851948	2.719362336	6.91E-03	4.95E-02
NFS1	-2.64524252	0.164721753	9.134823214	2.763432308	6.06E-03	4.53E-02
UBE2D2	-1.917992111	0.164946429	11.47219334	3.029513084	2.66E-03	2.57E-02
PPP2R5D	-2.680291258	0.165148052	10.44087662	2.749994448	6.31E-03	4.65E-02
TRA2B	2.585443374	0.165441558	11.75066494	4.347216219	1.88E-05	8.58E-04
BRIX1	-2.684596712	0.165512662	9.694835552	2.74833941	6.34E-03	4.66E-02
SKP1	-2.331744763	0.165927922	11.89948409	2.880981705	4.24E-03	3.55E-02
BCCIP	-2.056859927	0.166191234	10.09693458	2.980439688	3.11E-03	2.86E-02
OTUB1	-0.721431304	0.166669481	12.21978864	3.42505994	6.98E-04	1.02E-02
ZC3HC1	-1.899403581	0.166751948	8.709762987	3.036025298	2.60E-03	2.54E-02
DSTN	-2.471778843	0.167198701	12.2391039	2.829046514	4.98E-03	3.94E-02
UBE2E1	-2.576267477	0.167363961	11.01122873	2.789698085	5.61E-03	4.29E-02
GPS1	-2.755790409	0.167640909	11.58573929	2.720833493	6.88E-03	4.94E-02
PPP2R2D	-1.655926139	0.167684416	9.506754545	3.120144723	1.98E-03	2.07E-02
PSMD12	-2.003268043	0.167743831	9.461134903	2.999467618	2.93E-03	2.74E-02
USF1	-0.667785857	0.167832143	9.935959578	3.441801263	6.58E-04	9.75E-03
TMEM60	-2.451295912	0.168012338	8.677408766	2.836699326	4.86E-03	3.87E-02
HSPA4	-2.476563504	0.168054221	11.05532451	2.827256046	5.00E-03	3.95E-02
PRKRA	-1.460651585	0.168188636	10.31592094	3.186105271	1.59E-03	1.79E-02
COPG	-0.070828822	0.16830974	12.27104578	3.623278882	3.40E-04	6.26E-03
ZCCHC17	-2.711985095	0.16851039	9.767309091	2.737788918	6.55E-03	4.76E-02
CIDECP	-2.446347663	0.168811039	7.114155519	2.838545154	4.83E-03	3.87E-02
ARMC1	-2.514509372	0.168955195	9.865963961	2.813018086	5.22E-03	4.08E-02
SEC61A1	0.645357882	0.168984091	12.90029075	3.830590351	1.55E-04	3.59E-03
METTL5	-2.301169386	0.168998701	9.646386364	2.892203949	4.10E-03	3.46E-02
ARPC5	-2.434622493	0.169130519	12.32316331	2.842914422	4.77E-03	3.83E-02
PSMD1	0.49655168	0.169402597	10.90449935	3.78836626	1.82E-04	4.01E-03
ACOT8	-2.24827027	0.169939286	9.383387825	2.911523218	3.86E-03	3.32E-02
EIF5A	-2.605192914	0.170000325	12.45030925	2.778711943	5.79E-03	4.37E-02
COPS8	-2.148289631	0.170157792	10.38291591	2.947710407	3.45E-03	3.08E-02
NCSTN	-0.844354263	0.170188961	11.928575	3.386410702	8.00E-04	1.12E-02
C10orf84	-2.680729507	0.170334091	9.024845617	2.749826026	6.32E-03	4.65E-02
AKR1A1	-2.069878197	0.170601948	10.91330357	2.975800225	3.15E-03	2.89E-02
MESDC2	-1.637664067	0.171863636	10.13062857	3.126368517	1.94E-03	2.05E-02
PRKAG1	-0.81473725	0.171887013	10.27616818	3.395760034	7.74E-04	1.09E-02
NUP35	-2.081561242	0.17234513	8.542180357	2.971630803	3.20E-03	2.92E-02
UMPS	0.052120667	0.172745455	9.671135065	3.659628931	2.97E-04	5.70E-03
COPS3	-1.966912104	0.173008117	9.687289773	3.01231146	2.81E-03	2.66E-02
GPN3	-1.902563493	0.17316526	8.632437825	3.034919196	2.61E-03	2.54E-02
PCCB	-1.917511878	0.173205844	10.5521224	3.029681492	2.66E-03	2.57E-02
PYCR2	-2.148785625	0.173492208	10.71895325	2.947531921	3.45E-03	3.08E-02
RAD1	-2.365995197	0.173492208	9.201024026	2.868361292	4.41E-03	3.63E-02
TMEM9	-2.280685774	0.173553247	11.38958052	2.899699191	4.00E-03	3.41E-02
RPP30	-1.572821125	0.173634091	9.242606006	3.148374204	1.80E-03	1.95E-02
ILKAP	-0.914003167	0.174113961	8.674610227	3.364329577	8.65E-04	1.18E-02

SEC23B	-1.716998797	0.174133117	10.60894968	3.099245884	2.12E-03	2.18E-02
EIF6	-2.321215447	0.174656494	11.58193539	2.884850995	4.19E-03	3.52E-02
BAX	-2.394029815	0.174866558	9.016007955	2.857992019	4.55E-03	3.70E-02
DNTTIP1	-0.859815978	0.175097078	9.86912776	3.381520331	8.14E-04	1.13E-02
MFSD5	0.122049423	0.175113636	9.709112662	3.680156927	2.75E-04	5.42E-03
SFRS3	3.558392264	0.175191234	12.2615638	4.586787104	6.56E-06	4.17E-04
TMEM55B	-2.735605725	0.175450649	9.352398052	2.72865869	6.73E-03	4.85E-02
RAB1B	-0.22079445	0.175466883	12.4917737	3.578484439	4.01E-04	6.98E-03
WDR18	-2.64850414	0.175475974	9.553710714	2.762184408	6.09E-03	4.53E-02
VPS25	-2.511253517	0.175508766	10.16626737	2.814242418	5.20E-03	4.08E-02
GRK6	-1.622204151	0.176194805	10.2122526	3.131628272	1.91E-03	2.02E-02
C3orf26	-2.587822384	0.176467208	8.967689448	2.78531436	5.68E-03	4.32E-02
MGAT2	-1.063773881	0.176807792	9.454424675	3.316385907	1.02E-03	1.32E-02
SRP9	-1.783112129	0.176895779	12.5809362	3.076472217	2.28E-03	2.31E-02
BET1	-1.445882875	0.177533117	9.219206169	3.191042038	1.56E-03	1.78E-02
NSUN5	-1.469368653	0.177542857	9.608582468	3.183188044	1.61E-03	1.81E-02
RFWD2	0.068387839	0.177615584	10.17751883	3.664413627	2.92E-04	5.64E-03
TMED10	-1.847470615	0.177664286	12.90758344	3.054150073	2.45E-03	2.43E-02
AAMP	0.577167248	0.1778	11.20242468	3.811293754	1.67E-04	3.80E-03
TMEM53	-2.759706096	0.177902922	8.296181981	2.719312979	6.91E-03	4.95E-02
C7orf11	-2.299773653	0.177930519	9.166849026	2.89271525	4.09E-03	3.46E-02
SNRNP40	-2.180993249	0.178467532	9.831347403	2.935919924	3.58E-03	3.16E-02
TBL2	-1.000649484	0.178491234	10.26444432	3.336670797	9.52E-04	1.26E-02
GUSB	-2.580469793	0.178539286	10.42609302	2.788104559	5.63E-03	4.30E-02
STT3A	-1.728632633	0.178796753	11.58809838	3.095249838	2.15E-03	2.19E-02
PAIP1	-1.559053092	0.178947727	10.00852192	3.15302815	1.78E-03	1.93E-02
SRM	-2.150122234	0.179077273	11.18621721	2.947050884	3.45E-03	3.09E-02
TBCCD1	-2.759415567	0.179636688	8.673887175	2.719425823	6.91E-03	4.95E-02
C6orf136	-2.371149982	0.180304221	8.872335877	2.866457335	4.44E-03	3.64E-02
SUCLA2	-2.295605853	0.180544481	9.453076136	2.894241539	4.07E-03	3.45E-02
VPS72	-1.873795522	0.181029545	10.95315503	3.04497526	2.53E-03	2.48E-02
PIGK	-1.522277756	0.181463312	9.318336201	3.165427776	1.70E-03	1.87E-02
SEC13	-0.679398221	0.181505195	11.07563377	3.438183737	6.66E-04	9.86E-03
SNF8	-2.738585955	0.181618831	9.496978896	2.727504658	6.75E-03	4.86E-02
C2orf47	-0.607588315	0.181805195	8.652198701	3.460498261	6.16E-04	9.34E-03
KDELR2	-2.060205173	0.182092532	12.46408198	2.979248156	3.12E-03	2.87E-02
MRPS16	-1.618232908	0.182189286	11.01592256	3.132978029	1.90E-03	2.02E-02
PNO1	-0.613478432	0.182517532	9.465797078	3.458672943	6.20E-04	9.36E-03
ATF6B	-1.96451373	0.182861364	11.83472808	3.013156947	2.80E-03	2.66E-02
C3orf38	-0.456428639	0.184032143	9.201843344	3.507042109	5.21E-04	8.36E-03
UCK2	-1.961391596	0.184192532	9.139376136	3.014257244	2.79E-03	2.65E-02
SRI	-2.362830964	0.184477922	10.74758312	2.869529433	4.40E-03	3.62E-02
LZIC	-2.261998289	0.184583766	8.462904221	2.906521271	3.92E-03	3.36E-02
HNRNPC	2.073017512	0.184585714	13.10122273	4.216259992	3.27E-05	1.23E-03
CAP1	-2.359503083	0.185006818	12.51818458	2.870757501	4.38E-03	3.62E-02

SUMO2	-2.409829015	0.185230519	11.87145682	2.852132591	4.64E-03	3.75E-02
CHST12	-1.960583553	0.185875649	8.863428084	3.014541952	2.79E-03	2.65E-02
HEXB	-2.284241729	0.185928571	10.36564286	2.898399331	4.02E-03	3.42E-02
ARPC2	1.487061724	0.186069156	12.46994497	4.061942551	6.18E-05	1.85E-03
PSMG2	-1.173761052	0.186178571	10.2480237	3.280763668	1.15E-03	1.43E-02
MRPL18	-2.218939889	0.186807143	9.837363961	2.922182953	3.73E-03	3.24E-02
MDH2	-2.043488427	0.186849351	12.66880325	2.985197963	3.06E-03	2.83E-02
RAE1	-2.388156163	0.187487662	10.42913474	2.860167475	4.52E-03	3.69E-02
FLOT1	-2.614697604	0.187492208	12.20798701	2.775092965	5.86E-03	4.41E-02
MTERFD1	-2.147218913	0.187680844	8.905673539	2.948095676	3.44E-03	3.08E-02
TMED4	-1.494944083	0.18787987	10.80298929	3.174614601	1.65E-03	1.84E-02
UBE2J2	-1.663290558	0.188400325	10.3028664	3.117631606	2.00E-03	2.09E-02
NAA20	-1.212362783	0.188470779	10.0338211	3.268175944	1.21E-03	1.48E-02
PSMC1	-1.989441367	0.188853247	8.792816883	3.0043584	2.88E-03	2.71E-02
MIIP	-1.484699002	0.188941234	9.588344643	3.17805157	1.63E-03	1.83E-02
XAB2	-2.721713511	0.189033766	10.62032922	2.734032045	6.62E-03	4.80E-02
CDKN2AIPNL	-2.659469244	0.189353896	8.574117208	2.757985212	6.16E-03	4.57E-02
JMJD4	-1.881762952	0.189354545	9.115698701	3.042193312	2.55E-03	2.50E-02
AIP	-1.948143025	0.189464935	10.21962857	3.018922085	2.75E-03	2.63E-02
LSM12	0.062611803	0.18949513	10.34962679	3.662715364	2.94E-04	5.65E-03
ERP44	-1.788305877	0.189698377	10.92462256	3.074676456	2.30E-03	2.32E-02
FUCA1	-2.179952211	0.189749351	10.0352039	2.936295933	3.57E-03	3.15E-02
ZCCHC10	-0.936040666	0.189962338	8.219798052	3.357314914	8.86E-04	1.20E-02
SDF2	-1.557868136	0.19025487	9.566596266	3.153428395	1.77E-03	1.93E-02
EIF2B2	-1.116782382	0.19061039	9.449287013	3.299262327	1.08E-03	1.37E-02
EXOSC3	-1.094863973	0.190682143	8.76165211	3.30635272	1.06E-03	1.35E-02
TMEM39A	0.696777042	0.190725	9.840551461	3.845082925	1.47E-04	3.45E-03
SCAMP3	1.076403972	0.190835714	11.19207305	3.950592315	9.68E-05	2.56E-03
MON1A	-1.253914378	0.191041234	8.242256981	3.25457568	1.26E-03	1.52E-02
RABIF	-0.236111082	0.191055844	8.815032468	3.573880438	4.08E-04	7.04E-03
PIGF	-2.361345438	0.191230844	8.470614773	2.870077689	4.39E-03	3.62E-02
PSMD4	-0.428494394	0.191703571	11.03152256	3.515581237	5.05E-04	8.17E-03
B4GALT3	-0.683148995	0.191711364	10.20315179	3.43701453	6.69E-04	9.88E-03
TARBP2	-2.137404117	0.191755519	9.806339448	2.951625056	3.40E-03	3.06E-02
RWDD1	-2.315621089	0.191958766	9.021936526	2.886904803	4.17E-03	3.51E-02
TSG101	-1.077025492	0.192225649	10.42827776	3.312112888	1.04E-03	1.33E-02
TMEM134	-2.44544965	0.19226039	9.816941883	2.838880015	4.83E-03	3.87E-02
TAGLN2	-2.441841461	0.192468506	13.6374375	2.840225099	4.81E-03	3.85E-02
POLD2	-2.386747335	0.192613312	11.30639367	2.860689038	4.52E-03	3.69E-02
SUMO1P3	-0.427681393	0.192720779	8.82770974	3.515829475	5.04E-04	8.17E-03
PEX16	-1.258264291	0.192885714	8.86083961	3.253148849	1.27E-03	1.52E-02
UTP11L	-1.041620133	0.192952597	9.540625649	3.323518054	9.96E-04	1.30E-02
DPY30	-2.643145681	0.192953571	10.20840731	2.764234279	6.05E-03	4.52E-02
VPS29	-1.568003313	0.192962987	10.44371136	3.150003479	1.79E-03	1.94E-02
OBFC2B	-1.119540232	0.193218506	9.910704708	3.298369186	1.09E-03	1.38E-02

MRPS18C	-2.199640981	0.193262013	8.240360877	2.929176902	3.65E-03	3.20E-02
C14orf119	-1.84266661	0.193298377	10.15060568	3.055821584	2.44E-03	2.43E-02
PSMD2	-0.984602917	0.193424026	12.33700617	3.34180911	9.35E-04	1.24E-02
CCDC90A	-2.492483106	0.193593831	9.070224188	2.821291023	5.09E-03	4.01E-02
PTPMT1	-1.372425203	0.193856818	9.839260877	3.215491742	1.44E-03	1.68E-02
NSMCE4A	-1.76820195	0.193857468	9.127363799	3.081622021	2.25E-03	2.28E-02
ARF1	1.828444354	0.194001623	13.76600925	4.15247026	4.27E-05	1.48E-03
TXNDC12	-0.57783664	0.194211364	11.22442321	3.469704624	5.96E-04	9.17E-03
MRPL44	1.229217091	0.194437013	9.539373701	3.992353292	8.19E-05	2.27E-03
MED6	-0.332979862	0.195311364	8.770792045	3.544635756	4.54E-04	7.61E-03
FASTK	-1.532412869	0.19534026	10.8493461	3.162015034	1.72E-03	1.89E-02
ZDHHC16	-0.518750323	0.19556526	9.675296266	3.48792186	5.58E-04	8.76E-03
CLP1	1.86382933	0.195578896	8.178965422	4.161753141	4.11E-05	1.45E-03
RBM42	-2.256936617	0.195980195	10.81498425	2.90836649	3.90E-03	3.34E-02
STX5	4.25521429	0.19630974	10.38488019	4.751888241	3.10E-06	2.51E-04
EXT2	-0.329752847	0.196457143	10.56603247	3.545613558	4.53E-04	7.59E-03
C15orf23	-2.701543055	0.196652922	8.793502435	2.74181593	6.47E-03	4.73E-02
SNX3	-2.030250753	0.196732143	11.60119529	2.989901592	3.02E-03	2.79E-02
PSMD13	-0.286877011	0.197078896	10.62284659	3.558581722	4.32E-04	7.36E-03
NOSIP	-2.428500798	0.197275	9.681066071	2.845193086	4.74E-03	3.81E-02
FAM165B	-2.01393791	0.197436039	8.005314773	2.995688328	2.96E-03	2.76E-02
RFC2	-1.353418075	0.197528247	9.549163474	3.221789892	1.41E-03	1.66E-02
FUCA2	-1.41299658	0.197668831	10.24938961	3.202009474	1.51E-03	1.74E-02
MRPL37	-0.801794762	0.197969481	11.32964188	3.399838176	7.63E-04	1.08E-02
CDK4	-2.144446527	0.19797013	11.92324935	2.949093024	3.43E-03	3.08E-02
DYNLRB1	-1.47460735	0.198057468	11.93237289	3.181433676	1.62E-03	1.81E-02
BCL7B	0.094485685	0.198074675	10.24219253	3.672077942	2.84E-04	5.54E-03
FLAD1	0.206625932	0.198274675	10.487375	3.704846092	2.51E-04	5.04E-03
NFU1	-1.598559029	0.198380844	8.931321591	3.139656831	1.86E-03	1.99E-02
PSMD14	-0.396385424	0.198551948	10.18808961	3.525373013	4.87E-04	7.97E-03
COMMD4	-2.244664856	0.198599675	9.850568669	2.912835544	3.84E-03	3.31E-02
SLC33A1	0.137145619	0.198672727	9.512619481	3.684574799	2.71E-04	5.35E-03
SIAH2	-2.255799661	0.198682143	10.50266185	2.908780812	3.89E-03	3.34E-02
TRPT1	-1.807275887	0.198771104	9.190795942	3.068109114	2.35E-03	2.35E-02
UBE2N	-1.221847236	0.198791883	11.03051153	3.26507623	1.22E-03	1.49E-02
C18orf8	-2.155158673	0.198973052	8.587393019	2.945237638	3.47E-03	3.09E-02
ISCA2	-2.560124652	0.199096429	8.756807955	2.795811463	5.50E-03	4.24E-02
TMEM199	-1.900831949	0.199194481	8.333903734	3.035525356	2.61E-03	2.54E-02
TMEM189	-1.151898107	0.199427597	9.998748214	3.287873118	1.13E-03	1.41E-02
NFYC	-0.73873878	0.200027273	10.80546688	3.419642585	7.12E-04	1.03E-02
COX4NB	-0.897465458	0.200123052	9.244730357	3.369584737	8.49E-04	1.17E-02
C1orf74	-1.690397082	0.200386688	6.759852435	3.108365076	2.06E-03	2.13E-02
ADAM15	-2.554025467	0.200461039	11.53533182	2.798117959	5.46E-03	4.22E-02
GLA	-1.888359268	0.200645779	8.8106125	3.039888298	2.57E-03	2.51E-02
FUNDC2	-2.591948701	0.200786688	10.51210568	2.783747322	5.71E-03	4.33E-02

ZNF511	-2.566367785	0.200792208	9.416873377	2.793448659	5.54E-03	4.26E-02
C14orf166	-0.543033555	0.200797078	10.92839334	3.480445596	5.73E-04	8.94E-03
DNM2	-1.754263483	0.200928571	11.99694935	3.086428917	2.21E-03	2.25E-02
C17orf79	-0.457578199	0.201230844	9.204432955	3.506690293	5.21E-04	8.36E-03
RNF115	-1.029254302	0.201545455	9.477073377	3.32749292	9.83E-04	1.29E-02
COPS7B	0.812993773	0.201567857	9.907305357	3.877658136	1.29E-04	3.13E-03
TMEM11	1.264705069	0.201898377	9.036181006	4.001995445	7.88E-05	2.20E-03
COPS5	-0.920215395	0.202266234	10.08376299	3.362353561	8.71E-04	1.19E-02
APEX2	0.088042338	0.202425649	9.94426737	3.670187043	2.86E-04	5.56E-03
TMEM93	-2.657590379	0.202663312	9.13364789	2.758705173	6.15E-03	4.56E-02
MFF	2.468869757	0.202680519	10.40332273	4.31773284	2.13E-05	9.15E-04
SNRPB2	-1.010943433	0.202722078	10.28771818	3.333370676	9.63E-04	1.27E-02
ZNF14	-2.460380023	0.203149675	8.031039123	2.833307746	4.91E-03	3.91E-02
MCRS1	0.572805638	0.203209416	10.34074886	3.810056492	1.68E-04	3.80E-03
PIGC	-0.107722341	0.203217208	9.945887825	3.612306061	3.54E-04	6.40E-03
LRPAP1	-2.647109587	0.203234091	11.25193977	2.762718031	6.08E-03	4.53E-02
BPNT1	-2.352030419	0.203780844	9.445993669	2.873513287	4.34E-03	3.60E-02
MKKS	-0.829130288	0.203803247	9.795856818	3.3912195	7.87E-04	1.11E-02
PAQR3	-2.542257097	0.203877597	8.090066071	2.802563252	5.39E-03	4.18E-02
DNAJC9	-0.73015058	0.203960065	9.520828084	3.422331739	7.05E-04	1.02E-02
TUSC2	-0.392776062	0.204080844	9.625341071	3.526472141	4.85E-04	7.96E-03
UFC1	-1.652372857	0.204297078	11.02359594	3.121356608	1.97E-03	2.07E-02
SNORA5C	-1.942555927	0.204402597	0.512623377	3.020887279	2.73E-03	2.61E-02
MTCH1	-0.60019995	0.204428247	12.36212516	3.462786625	6.10E-04	9.31E-03
ORAI1	-1.243566615	0.204555844	9.447999351	3.257967551	1.25E-03	1.51E-02
NANS	-1.648345056	0.204655519	9.271029058	3.1227298	1.96E-03	2.07E-02
TMEM216	-1.41160084	0.204848052	8.641994156	3.202474172	1.51E-03	1.74E-02
GUK1	-2.67864729	0.20495	12.06489903	2.750626148	6.30E-03	4.64E-02
PDIA3P	-1.604748184	0.205448377	11.72677029	3.137557198	1.87E-03	2.00E-02
PFDN1	0.524516526	0.205549026	10.39660438	3.796333839	1.77E-04	3.94E-03
C7orf36	-1.742276259	0.205756818	8.015488799	3.090557289	2.18E-03	2.22E-02
JUND	-2.642477988	0.205759416	12.74901672	2.764489603	6.05E-03	4.52E-02
HDGF	1.852999196	0.205807792	13.8110026	4.158913925	4.15E-05	1.46E-03
PDSS1	-1.853443158	0.205882792	7.276506331	3.052070782	2.47E-03	2.44E-02
FEN1	-2.20073	0.205987987	9.462989448	2.92878266	3.66E-03	3.20E-02
GPR108	-2.59491225	0.205993831	10.4857112	2.782621348	5.73E-03	4.34E-02
RBX1	-2.474638161	0.206240584	9.722233279	2.827976658	4.99E-03	3.95E-02
APH1A	0.915580589	0.206275325	12.63718636	3.90620882	1.15E-04	2.91E-03
GPR89A	-0.609340406	0.206583766	9.330038636	3.45995539	6.17E-04	9.34E-03
PREB	0.731811849	0.206586364	10.42303474	3.854929287	1.41E-04	3.34E-03
CALU	-0.411628563	0.206904221	12.29003912	3.520727672	4.96E-04	8.06E-03
TSSC1	-1.650264901	0.20694026	9.456158442	3.122075341	1.97E-03	2.07E-02
PSMA6	-2.405648269	0.206975974	10.71647955	2.853684207	4.62E-03	3.74E-02
SUMO3	-1.541867162	0.207102922	11.87268263	3.158828434	1.74E-03	1.90E-02
SF4	4.16391599	0.207176623	9.525006494	4.730539531	3.42E-06	2.69E-04

ACP2	0.820538317	0.207205195	10.29814286	3.879764301	1.28E-04	3.12E-03
SDF4	-0.056435083	0.207484091	11.74565503	3.627551609	3.35E-04	6.20E-03
DNPEP	1.861269826	0.207573377	10.51370552	4.1610823	4.12E-05	1.45E-03
TTC9C	1.224059312	0.207574026	8.741808442	3.990950172	8.24E-05	2.28E-03
TMEM70	-1.722770374	0.207952922	8.940383604	3.097264039	2.13E-03	2.19E-02
SSNA1	-2.235603396	0.208044481	9.730785877	2.916131338	3.80E-03	3.28E-02
NUDCD2	-2.508626352	0.208443831	8.860561526	2.815229969	5.19E-03	4.07E-02
CHMP4B	1.34042609	0.208824026	11.77491461	4.022499632	7.25E-05	2.09E-03
RQCD1	0.652941388	0.208914935	8.553112013	3.832730894	1.54E-04	3.58E-03
CD63	-2.17642098	0.209283766	13.11929708	2.937571028	3.56E-03	3.15E-02
ATP5F1	-1.366017642	0.209417857	11.69132971	3.217616219	1.43E-03	1.68E-02
NUDT16L1	-2.302378221	0.209425325	9.687276299	2.891761046	4.10E-03	3.47E-02
BUD31	-0.951390169	0.209569156	10.68366445	3.352421043	9.01E-04	1.21E-02
POLR1C	-0.984846232	0.210281494	9.602948539	3.341731252	9.35E-04	1.24E-02
GTF2A2	-2.131869978	0.210293831	9.808637825	2.953613366	3.38E-03	3.05E-02
PFN1	-0.714128204	0.210517208	13.9354099	3.427343485	6.93E-04	1.01E-02
ALAS1	2.881735692	0.210795455	10.31384188	4.421374127	1.36E-05	6.82E-04
HSBP1	-2.192677365	0.21095974	11.18707013	2.931696659	3.62E-03	3.18E-02
C12orf32	-2.227080019	0.211129221	9.977216558	2.919228217	3.77E-03	3.26E-02
PHF11	-2.101428535	0.211253571	8.827315097	2.964527915	3.27E-03	2.97E-02
MRPS22	0.122225398	0.21191526	9.519241396	3.680208454	2.75E-04	5.42E-03
TIMM17A	-0.1676022	0.212095779	10.09692646	3.594431397	3.79E-04	6.71E-03
ALG14	-0.941225703	0.212097403	7.46805	3.355662513	8.91E-04	1.20E-02
DHRS7B	-1.321340591	0.212704545	8.45741461	3.232393047	1.36E-03	1.62E-02
BOD1	-1.423020243	0.212825974	10.05068701	3.198670345	1.52E-03	1.75E-02
DECR1	-2.422760164	0.212853896	9.452641883	2.847328339	4.71E-03	3.79E-02
LSM3	-1.559453671	0.213205519	10.38069237	3.152892835	1.78E-03	1.93E-02
PPIA	-0.880217289	0.213227273	12.43921169	3.375057583	8.33E-04	1.15E-02
NOMO1	-2.203967013	0.213254545	11.60472662	2.927610514	3.67E-03	3.21E-02
TTC13	-0.861656819	0.213380844	8.377473539	3.380937657	8.16E-04	1.13E-02
SLC25A3	-0.500481396	0.213461364	13.0328599	3.493536737	5.47E-04	8.65E-03
SOAT1	-2.512506053	0.213564935	8.755994156	2.813771474	5.21E-03	4.08E-02
RNF25	2.701576913	0.213877273	9.289879545	4.376414345	1.66E-05	7.87E-04
HARBI1	-0.142323102	0.213904221	6.101963149	3.601987341	3.68E-04	6.60E-03
GPR180	-1.516195018	0.213923377	8.339972727	3.167474333	1.69E-03	1.87E-02
C4orf27	-1.238985038	0.21397013	8.151611688	3.259468286	1.24E-03	1.50E-02
NOP16	-2.360471291	0.213974351	8.381670942	2.87040026	4.38E-03	3.62E-02
AGTRAP	-1.53226234	0.214014935	10.44871916	3.162065746	1.72E-03	1.89E-02
CHCHD3	0.824019468	0.214306169	10.10107841	3.880735765	1.27E-04	3.11E-03
CHID1	-0.01409809	0.214368506	10.50924399	3.640092581	3.20E-04	6.03E-03
ABCB8	-0.711851855	0.21438961	9.405828571	3.42805497	6.91E-04	1.01E-02
LOC401397	-2.156809617	0.214407792	9.253370779	2.944643026	3.48E-03	3.10E-02
RRAGC	-2.105181718	0.214497403	8.42171039	2.963184288	3.28E-03	2.98E-02
COQ6	0.073324003	0.21463539	8.434431981	3.665864383	2.90E-04	5.63E-03
ANAPC10	-1.253839115	0.214640909	6.948552273	3.254600362	1.26E-03	1.52E-02

C15orf24	-1.176073851	0.214869481	9.619230844	3.280010748	1.16E-03	1.43E-02
UNC119	-2.172211747	0.214888961	9.59058474	2.93909026	3.54E-03	3.14E-02
CREB3	-1.327549744	0.214956494	10.18169578	3.230343155	1.37E-03	1.62E-02
TRIAP1	-0.382783852	0.215205519	8.869443669	3.52951334	4.80E-04	7.90E-03
DTD1	-2.154268638	0.215538636	9.142262175	2.94555815	3.47E-03	3.09E-02
POP5	-1.815329875	0.215587013	8.571657792	3.065316863	2.37E-03	2.37E-02
LOC653566	-0.599628263	0.215684416	11.03199545	3.462963633	6.10E-04	9.31E-03
EIF4E2	3.082805515	0.21595487	10.39626899	4.471084085	1.10E-05	5.89E-04
CKAP2	-2.587254967	0.216045779	9.861449513	2.785529781	5.68E-03	4.31E-02
CAMTA1	-1.9911906	0.216103247	9.800583442	3.003740074	2.89E-03	2.71E-02
INPP1	-2.503714717	0.216183442	7.79098263	2.817075373	5.16E-03	4.05E-02
PRDX6	0.736681904	0.216608442	13.02986266	3.856296192	1.40E-04	3.33E-03
SAMD1	-1.039493236	0.216725649	10.21022581	3.324202037	9.94E-04	1.29E-02
TOE1	-0.691755301	0.216784091	8.529384903	3.434330349	6.76E-04	9.94E-03
C14orf147	-1.504926751	0.216930844	10.12768425	3.171262317	1.67E-03	1.85E-02
PDZD11	-0.954786096	0.217038961	9.883841558	3.351337428	9.05E-04	1.21E-02
TPI1	-2.076790099	0.21739026	13.37604838	2.973334185	3.18E-03	2.91E-02
HSPB11	-2.669684422	0.217399026	8.842203409	2.754067735	6.24E-03	4.61E-02
BSCL2	-0.684179056	0.217512338	11.19484448	3.436693372	6.70E-04	9.88E-03
TALDO1	-0.746156604	0.217632143	11.45651997	3.417318327	7.18E-04	1.04E-02
ACBD6	1.474254129	0.21774513	9.412501786	4.05851117	6.27E-05	1.87E-03
MRPS30	0.018317249	0.21778961	9.435916234	3.649667938	3.08E-04	5.85E-03
ZNF410	1.758406839	0.217873052	9.837079383	4.13404196	4.60E-05	1.56E-03
GLT25D1	-1.178667877	0.21804513	11.35412127	3.279166084	1.16E-03	1.43E-02
FAM119A	-1.790973877	0.218309416	8.323693019	3.073753601	2.30E-03	2.32E-02
THYN1	-2.449076262	0.218400649	9.48576526	2.837527455	4.85E-03	3.87E-02
H1FX	-2.18565366	0.218835714	12.18639123	2.93423609	3.60E-03	3.17E-02
NEDD8	-1.454005982	0.218896104	10.81772273	3.188327594	1.58E-03	1.79E-02
TMEM219	-0.766284203	0.218914286	10.9999474	3.411004289	7.34E-04	1.05E-02
TMEM183A	-0.082847804	0.219010714	10.59357094	3.619707557	3.45E-04	6.29E-03
DPH2	-0.48979818	0.219103571	9.855468019	3.49681633	5.40E-04	8.60E-03
PSMA1	0.757749441	0.219368831	11.10030455	3.86220429	1.37E-04	3.27E-03
SH3BGRL3	-1.647565022	0.219420455	12.27172062	3.12299567	1.96E-03	2.06E-02
C1orf43	0.580583261	0.219426299	12.37943458	3.812262524	1.66E-04	3.79E-03
DPP3	0.549266352	0.219518831	10.62998799	3.803372779	1.72E-04	3.86E-03
TRIT1	-1.986078384	0.219548052	9.116111688	3.005546825	2.87E-03	2.70E-02
DOM3Z	-1.84947535	0.219552273	9.212998864	3.053452289	2.46E-03	2.43E-02
ATP5C1	-1.155224153	0.21962987	12.36133961	3.286792468	1.13E-03	1.41E-02
FKBP3	-1.16441628	0.21963539	10.02562354	3.283804177	1.14E-03	1.42E-02
GTF2H4	0.928405316	0.21973539	9.413364448	3.909764803	1.14E-04	2.89E-03
PIGU	0.463747317	0.219865909	9.957223214	3.779000405	1.89E-04	4.13E-03
MRPS36	-2.092186861	0.220026948	7.980062825	2.96783396	3.23E-03	2.95E-02
CDC123	-0.213593436	0.22005	10.48276656	3.580647104	3.98E-04	6.95E-03
SERP1	2.536864491	0.220280844	12.61992614	4.334951289	1.98E-05	8.85E-04
BCAS2	0.069615306	0.220498701	9.538132468	3.664774433	2.92E-04	5.64E-03

ADRM1	1.283288125	0.220857792	11.30808734	4.007036189	7.72E-05	2.19E-03
CREM	-0.759608956	0.220871104	8.804201136	3.413099514	7.28E-04	1.05E-02
POLE4	-2.469643567	0.220887662	8.776889935	2.829845209	4.96E-03	3.93E-02
MAD2L1BP	-0.492524249	0.220949675	8.851879383	3.495979735	5.42E-04	8.60E-03
CCDC123	-1.666788855	0.220966883	9.263389935	3.116437142	2.00E-03	2.09E-02
HPRT1	-2.72582496	0.221019805	9.566317695	2.732442829	6.65E-03	4.81E-02
TSPAN17	0.196604945	0.221203247	10.19791396	3.701928657	2.54E-04	5.09E-03
DCPS	-0.709209209	0.221322403	8.297830682	3.428880772	6.89E-04	1.01E-02
GEMIN7	-2.081383981	0.221410065	8.512356981	2.971694105	3.20E-03	2.92E-02
TRAIP	-2.439385828	0.221448377	7.677209903	2.84114018	4.80E-03	3.85E-02
RNF7	1.709644678	0.221604545	10.59452955	4.121168309	4.85E-05	1.59E-03
YARS2	-0.539064612	0.221666883	9.105225649	3.481668556	5.71E-04	8.92E-03
CCDC72	-2.628666989	0.221712987	10.04614221	2.769765875	5.95E-03	4.46E-02
EFNA4	-2.681701609	0.222290909	8.781278571	2.749452406	6.32E-03	4.65E-02
KLHDC3	-1.793916533	0.222818182	11.51465325	3.072735441	2.31E-03	2.32E-02
AP1S1	-0.377563739	0.222882468	9.844893182	3.531101161	4.77E-04	7.88E-03
ABCB6	-2.488560048	0.222922403	9.14393263	2.822762089	5.07E-03	4.00E-02
GLB1	0.758782395	0.222966234	11.06009416	3.862493758	1.37E-04	3.27E-03
SRP19	2.006923217	0.223186364	8.951994481	4.199106124	3.51E-05	1.29E-03
VDAC2	-1.724446424	0.223233442	11.61479269	3.096688294	2.14E-03	2.19E-02
ZNF684	-2.175914785	0.223350974	6.436031981	2.937753767	3.56E-03	3.15E-02
CHCHD8	-2.576518594	0.22341526	10.36466542	2.789602886	5.61E-03	4.29E-02
BRMS1	1.697857313	0.223533766	9.264207792	4.118050955	4.92E-05	1.61E-03
B3GAT3	-0.469853104	0.223659091	9.926456818	3.502931613	5.29E-04	8.45E-03
B9D2	-2.700030928	0.223853896	7.224489935	2.742398621	6.46E-03	4.72E-02
COX16	-1.22450803	0.223935714	9.681309416	3.264206136	1.22E-03	1.49E-02
ERAL1	-0.763249387	0.224107792	10.14104286	3.411957004	7.31E-04	1.05E-02
ATL3	-1.95411717	0.224707792	9.268688961	3.01681942	2.77E-03	2.64E-02
CLTB	-2.321529303	0.224954221	10.22201867	2.884735731	4.19E-03	3.52E-02
RRAGB	-1.845717536	0.225397403	8.151991558	3.054760141	2.45E-03	2.43E-02
SELS	-1.253612171	0.225491883	9.484944643	3.254674786	1.26E-03	1.52E-02
MRPL53	-2.207553865	0.225898052	9.576536039	2.926311171	3.69E-03	3.22E-02
IFT52	-1.034115603	0.226032468	9.843227922	3.325930832	9.88E-04	1.29E-02
CFL1	1.818780071	0.226097078	14.58355633	4.149931724	4.31E-05	1.49E-03
RAB8A	0.34517789	0.226371753	11.33645406	3.744970733	2.15E-04	4.51E-03
RAN	-0.302895828	0.226496104	12.19808961	3.553741779	4.40E-04	7.46E-03
NUDT22	-2.05627644	0.226712662	9.163414773	2.980647472	3.11E-03	2.86E-02
TUBB	-0.399678931	0.226823052	14.8986875	3.524369795	4.89E-04	7.99E-03
SEPX1	-2.282989751	0.226882792	8.659951136	2.898857048	4.01E-03	3.41E-02
THOC3	-2.111203921	0.227004545	10.48283279	2.961027159	3.31E-03	3.00E-02
FAM36A	-1.698138222	0.227078571	9.922330844	3.105713966	2.08E-03	2.14E-02
BPGM	-1.370232039	0.227096104	8.798708442	3.21621905	1.44E-03	1.68E-02
UBE2F	0.523128758	0.227292532	9.331505357	3.795938803	1.77E-04	3.94E-03
ECHS1	-1.636203387	0.227294481	11.44366412	3.126865822	1.94E-03	2.05E-02
MRPL1	-0.833482515	0.227327273	8.263331169	3.389845406	7.91E-04	1.11E-02

PPP1CA	0.767055067	0.227435065	12.33944675	3.864811323	1.36E-04	3.25E-03
SEC22B	2.963634135	0.227436364	10.25617597	4.44168041	1.25E-05	6.43E-04
TUBG1	-0.843363101	0.227475974	9.824378896	3.386723973	7.99E-04	1.12E-02
STOML2	-1.248482975	0.227504221	11.07150471	3.256356438	1.25E-03	1.51E-02
DPM1	1.39121077	0.227741234	9.850449838	4.036199066	6.86E-05	2.01E-03
COX7A2L	-0.22172679	0.227786364	11.32500097	3.578204345	4.02E-04	6.98E-03
PPT1	-1.529663021	0.228162013	12.10056672	3.162941316	1.72E-03	1.89E-02
HMGB3	-2.543517093	0.228491883	10.08423555	2.802087631	5.40E-03	4.18E-02
BAD	-0.235955621	0.228519156	10.10725438	3.573927195	4.08E-04	7.04E-03
ADI1	-0.545251408	0.22863961	11.12011071	3.479762031	5.74E-04	8.94E-03
MSRB2	-2.249282909	0.228691883	9.188846591	2.91115453	3.86E-03	3.33E-02
TMEM50B	-1.322696853	0.228771104	10.02254399	3.231945393	1.36E-03	1.62E-02
FAM98C	-2.368732249	0.228952597	8.307803571	2.867350491	4.43E-03	3.63E-02
NUP37	-0.614486525	0.229046429	8.61299724	3.458360451	6.20E-04	9.36E-03
SERF2	-2.100646524	0.229062662	13.02147419	2.9648078	3.27E-03	2.97E-02
CCDC107	-1.22412554	0.22938961	8.391386364	3.264331225	1.22E-03	1.49E-02
CCNYL1	-0.221185094	0.229645455	8.74600974	3.578367084	4.02E-04	6.98E-03
RHOC	-0.876163429	0.229797078	11.98196607	3.376342681	8.29E-04	1.15E-02
ALKBH6	-1.951702426	0.229866558	8.526970942	3.017669481	2.76E-03	2.63E-02
PSMB1	-1.366671144	0.229969156	11.14591834	3.217399605	1.43E-03	1.68E-02
SIP1	-1.565102826	0.230071104	7.308166721	3.150983978	1.79E-03	1.94E-02
FAM195B	-1.933800456	0.230188636	10.76148263	3.023964495	2.71E-03	2.60E-02
VKORC1	-0.992382056	0.230231169	10.71811818	3.339319047	9.43E-04	1.25E-02
C3orf1	2.522453932	0.230313312	11.23007159	4.331307109	2.01E-05	8.87E-04
PRIM2	-1.123127584	0.230347403	8.244682143	3.297207072	1.09E-03	1.38E-02
VRK1	-1.45580743	0.230369805	8.47725763	3.187725324	1.58E-03	1.79E-02
SGOL2	-2.580599043	0.230546429	8.023812825	2.788055533	5.63E-03	4.30E-02
MGAT1	0.87089452	0.230547078	11.69047549	3.893795582	1.21E-04	3.02E-03
SNRPD1	-1.326234803	0.230566883	9.727395779	3.230777371	1.37E-03	1.62E-02
MORN2	-2.482101119	0.230577273	8.420563961	2.825182473	5.03E-03	3.97E-02
MRPS23	-2.128191505	0.230612338	9.45081526	2.954934274	3.37E-03	3.04E-02
TMEM208	-0.98407186	0.230673377	8.835056169	3.341979036	9.35E-04	1.24E-02
BANF2	-2.350033461	0.230793506	0.558394156	2.874249307	4.33E-03	3.60E-02
UROD	0.454621943	0.23087013	10.07953247	3.776391303	1.91E-04	4.15E-03
HEXIM2	-2.24598565	0.231127597	6.702983279	2.912354856	3.85E-03	3.32E-02
C11orf59	-0.074512765	0.231156494	11.46457175	3.622184576	3.42E-04	6.28E-03
GMPS	0.006782603	0.231160065	10.69904302	3.646263287	3.12E-04	5.91E-03
PDIA3	-0.452711984	0.231387662	12.97465162	3.508179344	5.19E-04	8.35E-03
COMMD3	-1.641573068	0.231623377	9.289692857	3.125037289	1.95E-03	2.05E-02
TXNDC16	-2.446914511	0.23188474	8.535803409	2.838333762	4.84E-03	3.87E-02
RNASEH2B	-0.223657142	0.231925325	8.531750325	3.577624361	4.03E-04	6.99E-03
UBE2A	0.227957725	0.23193539	10.25035016	3.711049508	2.45E-04	4.95E-03
HSPA14	0.605688215	0.232190909	9.277938961	3.81937537	1.62E-04	3.71E-03
MRPL49	1.29987015	0.232377922	10.89723117	4.011529345	7.58E-05	2.16E-03
PGAM1	-0.797378459	0.232543182	11.96862679	3.401228706	7.60E-04	1.08E-02

ABT1	0.058456763	0.23254513	9.861155032	3.661493259	2.95E-04	5.67E-03
LRRC42	0.522140699	0.23283539	9.92945211	3.795657523	1.77E-04	3.94E-03
SNRPD2	-2.72319126	0.232855195	11.84433474	2.733460946	6.63E-03	4.80E-02
RACGAP1	-2.436344776	0.232880519	10.12650195	2.842273029	4.78E-03	3.84E-02
ALG8	-2.556847434	0.233470455	10.19952744	2.797051015	5.48E-03	4.23E-02
CDC37	-0.553221931	0.233638961	11.64151494	3.477304413	5.80E-04	8.99E-03
EBPL	-2.367275759	0.233738312	9.18039513	2.867888419	4.42E-03	3.63E-02
COMMD8	-1.582258641	0.234150649	8.239081169	3.145180362	1.82E-03	1.96E-02
CNPY2	0.652547912	0.234194481	10.9296901	3.832619857	1.54E-04	3.58E-03
DERL2	0.344362665	0.234321429	8.994234091	3.744735789	2.16E-04	4.51E-03
HIGD1A	-1.418965253	0.234627922	11.28143669	3.200021554	1.52E-03	1.75E-02
GINS1	-2.68930552	0.23532987	8.783383117	2.746528236	6.38E-03	4.68E-02
MID1IP1	-1.501716801	0.235395779	10.22010438	3.172340613	1.67E-03	1.85E-02
PRDX1	0.102284565	0.235466883	12.51254773	3.674365454	2.81E-04	5.52E-03
MRPL48	-1.868561174	0.236047078	9.360733929	3.046801613	2.51E-03	2.47E-02
PQBP1	-2.405476749	0.23611039	10.21446169	2.853747847	4.61E-03	3.74E-02
MPHOSPH6	0.300180771	0.236372078	7.756178247	3.731982643	2.26E-04	4.67E-03
C17orf49	-0.51296376	0.236482143	10.58391899	3.489701236	5.54E-04	8.74E-03
TRAPPC2L	-2.387374208	0.237041558	8.720838961	2.860456974	4.52E-03	3.69E-02
WDR77	-2.262941308	0.237122403	10.40323718	2.906177373	3.92E-03	3.36E-02
GET4	-0.571946884	0.237353247	10.14163701	3.471524482	5.92E-04	9.13E-03
ZNF85	-2.151308146	0.237740909	7.374310065	2.94662402	3.46E-03	3.09E-02
CBR1	-1.77262047	0.23782987	10.0267513	3.080096758	2.26E-03	2.29E-02
SNAPIN	0.095807743	0.238250649	9.872918831	3.67246581	2.83E-04	5.54E-03
ST7	1.198840012	0.238276623	9.581219481	3.984083139	8.47E-05	2.32E-03
EEF1E1	-1.476099794	0.238661364	8.661041071	3.180933711	1.62E-03	1.82E-02
B4GALT7	1.599884826	0.239337013	9.101517208	4.092058645	5.47E-05	1.70E-03
CHEK2	-1.924251852	0.239355195	8.257393182	3.027317114	2.68E-03	2.58E-02
MED10	-1.102943868	0.239391883	9.065810877	3.303740598	1.07E-03	1.36E-02
C11orf84	1.186807305	0.239409091	9.651469481	3.98080297	8.58E-05	2.34E-03
BANF1	-0.610676122	0.239411039	12.33178539	3.459541476	6.18E-04	9.34E-03
ALS2CR4	-0.982288733	0.239517857	9.146209578	3.342549536	9.33E-04	1.24E-02
PSMA3	0.569141611	0.239598377	10.03182906	3.809016832	1.68E-04	3.80E-03
CDC34	-0.39494619	0.239688961	9.720140584	3.525811328	4.87E-04	7.97E-03
TBCB	-1.40007841	0.239699026	10.81964821	3.206308049	1.49E-03	1.73E-02
DIRC2	-0.513892644	0.239720779	8.741885065	3.489415659	5.55E-04	8.74E-03
POLR3K	-1.333868682	0.239783117	8.029832468	3.228255772	1.38E-03	1.63E-02
TXNL4A	-0.417833774	0.239802597	9.979718182	3.518835021	4.99E-04	8.10E-03
LSM6	-1.235275047	0.23996039	8.240691234	3.260683055	1.24E-03	1.50E-02
C14orf1	0.939827923	0.239986039	10.48781899	3.912929558	1.12E-04	2.86E-03
TMEM179B	-1.063080066	0.240068831	11.22315519	3.316609489	1.02E-03	1.32E-02
WBSCR22	0.780207106	0.24007013	10.41357208	3.868493246	1.34E-04	3.22E-03
LIN37	-0.473228361	0.240111039	8.089002273	3.501897429	5.30E-04	8.46E-03
PPIE	-0.335144611	0.24011461	9.75393263	3.543979687	4.55E-04	7.62E-03
POLR2H	0.581754127	0.240495455	10.60101721	3.812594526	1.66E-04	3.79E-03

CTPS	-2.202429771	0.240622403	9.563519643	2.928167216	3.66E-03	3.20E-02
SDHC	1.167816952	0.240805844	11.13216656	3.97562117	8.76E-05	2.38E-03
ARPC3	0.51999291	0.241038312	11.65402825	3.795046026	1.78E-04	3.94E-03
NME2	-2.001392165	0.241082143	12.78859172	3.000131595	2.92E-03	2.74E-02
CSNK2B	1.191524196	0.241151623	12.15175698	3.982089105	8.53E-05	2.33E-03
H3F3A	-0.03585949	0.241157143	12.86039091	3.633651412	3.27E-04	6.10E-03
MRPL39	-1.025970515	0.241185714	8.734815584	3.328547719	9.79E-04	1.28E-02
SDSL	-2.57985567	0.241408442	8.078569156	2.788337489	5.63E-03	4.30E-02
DCUN1D5	-0.109772912	0.241441234	9.020878409	3.611695288	3.55E-04	6.41E-03
DCTN3	-0.961070823	0.241666558	10.18657484	3.34933116	9.11E-04	1.22E-02
ZNF490	-2.03870303	0.241837338	8.177308279	2.986899122	3.05E-03	2.82E-02
COMMD7	1.359809355	0.241984091	10.68768295	4.027733297	7.10E-05	2.06E-03
DERA	-2.465475339	0.242120779	9.484938312	2.83140371	4.94E-03	3.92E-02
C6orf108	-2.689047604	0.242276948	10.04166964	2.746627469	6.38E-03	4.68E-02
PL-5283	0.483579154	0.242624675	11.07702208	3.784665037	1.85E-04	4.05E-03
FTSJ1	-0.856457529	0.242781169	9.928237338	3.382583128	8.11E-04	1.13E-02
MTCH2	4.736808431	0.243232792	10.79726769	4.863170057	1.85E-06	1.76E-04
ZNF593	-1.595487265	0.243272727	9.196196104	3.140698421	1.85E-03	1.99E-02
OSTCL	-2.718325519	0.243324351	2.496254383	2.735340961	6.59E-03	4.78E-02
AKIRIN1	-2.271355275	0.243387662	10.73145942	2.903107289	3.96E-03	3.38E-02
NDUFV3	0.613206136	0.243423701	9.833685877	3.821503046	1.61E-04	3.68E-03
CDK16	-2.015210227	0.243447078	12.29911769	2.995237371	2.97E-03	2.76E-02
NFKBIB	-2.638464023	0.243612338	8.915126948	2.766024059	6.02E-03	4.50E-02
MRPL54	-2.385028155	0.243921104	8.547213149	2.861325374	4.51E-03	3.68E-02
C13orf34	-1.9782781	0.244086039	7.784117045	3.00830162	2.84E-03	2.69E-02
AP2S1	-0.634176981	0.244116234	10.3701224	3.452251502	6.34E-04	9.51E-03
TRAPPC3	-0.176154435	0.244178247	10.43748263	3.591871829	3.82E-04	6.75E-03
TAF11	0.748172568	0.24424513	9.424055682	3.859519603	1.39E-04	3.30E-03
MED26	-0.02084543	0.244355519	9.148592045	3.638096548	3.22E-04	6.04E-03
NUDT19	-1.659187256	0.244567857	9.025364448	3.119032097	1.99E-03	2.08E-02
GLRX5	-0.609424114	0.244744156	10.47411429	3.459929451	6.17E-04	9.34E-03
CKLF	-2.482365169	0.244793831	8.907781981	2.825083563	5.04E-03	3.97E-02
TMEM126A	-1.935085969	0.244837662	8.076483766	3.02351287	2.71E-03	2.60E-02
MYL6	-1.357243165	0.244898052	14.17591461	3.220523344	1.42E-03	1.66E-02
STIL	-2.678154554	0.244909091	8.882735065	2.750815456	6.30E-03	4.64E-02
TOMM6	-1.421431503	0.24522013	11.32554903	3.199199811	1.52E-03	1.75E-02
C19orf63	-1.398211098	0.245225	11.85663393	3.206928959	1.48E-03	1.72E-02
SNX22	-1.014382176	0.245399026	7.444491721	3.33226758	9.67E-04	1.27E-02
CNIH	-1.057785293	0.245482792	10.74438166	3.318315268	1.01E-03	1.32E-02
NUDT15	-1.060608079	0.245530519	9.312569805	3.317405973	1.02E-03	1.32E-02
GLRX2	-1.081353436	0.245771104	6.751491396	3.31071622	1.04E-03	1.33E-02
CIB1	-1.398660369	0.245782792	10.56454919	3.20677958	1.48E-03	1.72E-02
FH	0.399443605	0.246007143	10.27506916	3.760579841	2.03E-04	4.34E-03
SLC39A7	1.830232762	0.246068831	11.09062597	4.152939873	4.26E-05	1.48E-03
MRPS14	1.867035725	0.246087013	9.028822078	4.162593395	4.09E-05	1.45E-03

POLA2	0.043208347	0.24616461	9.353908279	3.657005108	3.00E-04	5.73E-03
POLR2I	-1.549207265	0.246463636	10.15287857	3.156352355	1.76E-03	1.92E-02
CNIH4	1.518821217	0.246794156	9.645787338	4.070440315	5.97E-05	1.81E-03
ACOT7	-2.363226388	0.246836039	9.013395942	2.869383478	4.40E-03	3.62E-02
C8orf38	-0.886627462	0.246897727	8.237257955	3.373024594	8.39E-04	1.16E-02
C13orf37	-0.941946618	0.247643831	9.098456981	3.355432707	8.92E-04	1.20E-02
CCDC99	0.268722654	0.247768506	8.458429058	3.722877996	2.34E-04	4.79E-03
C12orf62	-1.112116037	0.247928896	9.125115747	3.30077303	1.08E-03	1.37E-02
C1orf151	0.87745516	0.248167208	9.837525812	3.895620283	1.20E-04	3.00E-03
ZNF823	-1.24066436	0.248364935	7.203658442	3.258918284	1.24E-03	1.50E-02
SLC25A4	-0.543760891	0.248505519	9.639837175	3.480221438	5.74E-04	8.94E-03
RHEB	0.288673467	0.248929221	10.72363084	3.728654533	2.29E-04	4.72E-03
PDRG1	0.067324136	0.249177597	9.199923864	3.664100932	2.92E-04	5.64E-03
BRI3	0.24587767	0.249206169	10.53129854	3.716253444	2.40E-04	4.88E-03
TUBA1B	0.524464853	0.249422078	14.90636104	3.796319131	1.77E-04	3.94E-03
C12orf45	-1.465807184	0.249596429	7.589916396	3.184380216	1.60E-03	1.80E-02
ROBLD3	-0.876170412	0.249719481	9.657935065	3.376340467	8.29E-04	1.15E-02
DTYMK	-0.37823367	0.249830844	9.458310877	3.530897422	4.78E-04	7.88E-03
SDHB	1.274887071	0.249846429	10.82437451	4.004758068	7.79E-05	2.20E-03
LSR	-2.605479142	0.250631818	12.51675682	2.778603025	5.80E-03	4.37E-02
MRPL16	1.999759236	0.250893831	9.611507955	4.197243075	3.54E-05	1.29E-03
NR1H3	-0.930989822	0.250916234	8.821529545	3.358923826	8.81E-04	1.19E-02
SUGT1	0.645670454	0.251168831	9.141531818	3.8306786	1.55E-04	3.59E-03
C7orf28B	-1.208921321	0.251212013	9.790080682	3.269300009	1.20E-03	1.47E-02
PGP	-1.156443248	0.251278571	9.416160714	3.286396294	1.13E-03	1.42E-02
PIAS2	-0.663457665	0.251377273	8.502465909	3.4431487	6.55E-04	9.73E-03
GPI	-1.179083416	0.251443182	12.99797159	3.279030758	1.16E-03	1.43E-02
PIGT	0.654622744	0.251580519	12.60361753	3.833205332	1.53E-04	3.58E-03
FKBP8	1.940872851	0.251748701	12.89680357	4.181901291	3.78E-05	1.36E-03
LOC152217	-1.43513226	0.251907468	9.387458929	3.194631188	1.55E-03	1.77E-02
C14orf109	-0.782065382	0.252442857	8.613233766	3.406046157	7.47E-04	1.07E-02
METRNL	-1.759721303	0.252455844	9.479043506	3.08454754	2.22E-03	2.26E-02
NRL	-1.354033594	0.252572403	5.181463474	3.221586115	1.41E-03	1.66E-02
HYI	-1.422970327	0.252691558	8.39656461	3.198686981	1.52E-03	1.75E-02
SLC37A1	-2.592534471	0.252721104	8.821598864	2.783524798	5.71E-03	4.33E-02
CHCHD6	-1.458722818	0.252782792	7.606214773	3.186750409	1.59E-03	1.79E-02
ASPSCR1	-0.392643013	0.25293961	9.245426948	3.526512651	4.85E-04	7.96E-03
MRPL43	1.224730202	0.253389935	10.19622873	3.991132706	8.23E-05	2.28E-03
POMP	-0.168803892	0.253477273	10.61195162	3.594071848	3.79E-04	6.71E-03
PHPT1	-2.065121	0.253506494	10.41464935	2.977496389	3.14E-03	2.88E-02
ZBTB8OS	-0.32482823	0.253697727	7.625024838	3.547105264	4.50E-04	7.56E-03
C2orf28	-0.29506206	0.253717532	11.81311526	3.556109438	4.36E-04	7.41E-03
RNF181	0.236721846	0.254138961	10.5656526	3.713595431	2.43E-04	4.92E-03
CNPY3	0.783949173	0.254171753	11.05389562	3.869540261	1.33E-04	3.21E-03
MAGEB18	-2.186862317	0.254296104	0.369338961	2.933799246	3.60E-03	3.17E-02

PSMB5	0.374875069	0.254591558	11.2282289	3.753520206	2.08E-04	4.42E-03
TAF10	-0.484740068	0.25468961	11.11450195	3.49836811	5.37E-04	8.56E-03
SELT	0.746220481	0.254758766	11.05495016	3.858972166	1.39E-04	3.30E-03
DNAJC19	1.290449109	0.254811688	9.306794156	4.008977123	7.66E-05	2.18E-03
AURKAIP1	-0.08385175	0.254864935	10.86058571	3.619409098	3.45E-04	6.29E-03
NIF3L1	3.868550796	0.254899675	9.638961526	4.660897532	4.70E-06	3.33E-04
EMG1	-2.08280506	0.255224026	9.327217857	2.971186587	3.20E-03	2.92E-02
SSR3	3.215212504	0.255297078	11.88070308	4.503556612	9.50E-06	5.36E-04
LIPT2	-2.564580818	0.255594805	6.713718182	2.794125156	5.53E-03	4.25E-02
MRPS28	-0.642732786	0.255670455	8.51280276	3.449593959	6.40E-04	9.58E-03
GMPPA	7.632919393	0.256137662	9.711446104	5.491913858	8.35E-08	1.99E-05
DBF4	-0.417507053	0.25618539	8.2410375	3.518934698	4.99E-04	8.10E-03
MRPL55	0.800244248	0.256213636	9.43330487	3.874096577	1.31E-04	3.17E-03
PSMB2	1.873856655	0.256550325	11.79335049	4.164380357	4.06E-05	1.44E-03
MMACHC	2.035100788	0.256732468	8.127601948	4.206426816	3.41E-05	1.27E-03
CRIPT	1.565461154	0.256835714	9.47998539	4.082890913	5.68E-05	1.75E-03
TRIP13	-2.738286308	0.257052597	9.364578896	2.727620711	6.75E-03	4.86E-02
ASNS	-1.815464886	0.257076299	9.652428409	3.065270035	2.37E-03	2.37E-02
MREG	-2.34973029	0.257109416	8.657178084	2.874361031	4.33E-03	3.60E-02
LGMN	-0.375197605	0.25713539	11.09067679	3.53182066	4.76E-04	7.87E-03
TAF13	-1.264585049	0.257142532	6.896024513	3.251074514	1.28E-03	1.53E-02
DYNLL1	4.026957015	0.257149351	12.23045065	4.698357534	3.96E-06	2.97E-04
EBNA1BP2	2.074091818	0.257206818	10.92509237	4.216538302	3.27E-05	1.23E-03
COMMD10	-0.608106927	0.257502922	8.552443019	3.460337582	6.16E-04	9.34E-03
OAZ1	-0.39168678	0.257620455	13.25753231	3.526803789	4.85E-04	7.96E-03
FIBP	2.89930393	0.257656169	11.08123912	4.425736951	1.34E-05	6.71E-04
MRPL21	-0.859118086	0.25781039	9.647365584	3.381741207	8.13E-04	1.13E-02
ERGIC3	1.733810455	0.257826623	12.92188214	4.127552765	4.73E-05	1.57E-03
MAD2L2	-2.255673993	0.257921429	9.611900974	2.908826603	3.89E-03	3.34E-02
RPP38	3.848410497	0.257968182	9.280881494	4.656116121	4.80E-06	3.38E-04
UROS	-0.385115829	0.258208442	9.72995487	3.528803802	4.81E-04	7.92E-03
MRPS18A	1.029285264	0.258309416	10.04744432	3.937635339	1.02E-04	2.66E-03
MRS2	1.483401241	0.258427597	9.909340422	4.060962107	6.21E-05	1.86E-03
HM13	4.633995988	0.258596429	12.13459172	4.839596908	2.06E-06	1.87E-04
PTMA	4.609048326	0.25888961	14.57424935	4.833862054	2.12E-06	1.89E-04
SPCS1	1.692163971	0.259035065	11.24559805	4.116544508	4.95E-05	1.61E-03
GALK1	-1.213610922	0.25967987	8.396637338	3.267768183	1.21E-03	1.48E-02
MED19	1.943138968	0.259929221	8.699818506	4.182492611	3.77E-05	1.36E-03
PSMA2	2.141681375	0.260010065	11.16671023	4.234015455	3.04E-05	1.17E-03
MEMO1	3.372805026	0.260281818	8.6808	4.541941797	8.02E-06	4.80E-04
CLIC1	2.235614578	0.260366883	12.6827711	4.258198914	2.74E-05	1.10E-03
ZNF791	-0.933466905	0.260441558	8.438669481	3.358134858	8.84E-04	1.20E-02
CCDC159	-1.497869594	0.260573701	7.27414789	3.173632527	1.66E-03	1.85E-02
H2AFZ	-1.060583544	0.260678571	11.26201591	3.317413877	1.02E-03	1.32E-02
RNASEH2C	0.161055974	0.260901948	9.772563961	3.691562243	2.64E-04	5.24E-03

SLC35A2	-1.121236165	0.261055844	10.33221039	3.29781984	1.09E-03	1.38E-02
C11orf48	0.611917661	0.261057143	9.521251948	3.821138466	1.61E-04	3.68E-03
BRP44L	-0.802488235	0.261575974	8.793054221	3.399619779	7.64E-04	1.08E-02
MRPL51	-1.030766798	0.261702922	10.81604107	3.327006981	9.84E-04	1.29E-02
ILF2	4.644316542	0.261863312	12.49567192	4.841967654	2.04E-06	1.87E-04
PARL	2.842953005	0.261956169	9.918851461	4.411729611	1.42E-05	7.06E-04
PFDN4	-0.936498129	0.262007468	8.992695942	3.357169157	8.86E-04	1.20E-02
POLR2G	0.781214216	0.262060065	10.26154237	3.868775056	1.34E-04	3.22E-03
RHBDD2	-0.656614384	0.262288636	11.87224821	3.445278132	6.50E-04	9.67E-03
PFDN2	1.668684862	0.262335065	9.514018182	4.110326777	5.07E-05	1.61E-03
EIF3K	-2.48003118	0.262478247	11.79659756	2.825957736	5.02E-03	3.96E-02
SNRNP25	-0.599637445	0.262711364	8.662594643	3.46296079	6.10E-04	9.31E-03
MRPS17	1.849393335	0.26271461	8.390815747	4.157968234	4.17E-05	1.46E-03
CEBPG	-0.877671116	0.262795455	9.958152273	3.375864788	8.30E-04	1.15E-02
PSMB6	1.6766665	0.262832792	10.38591899	4.112441412	5.03E-05	1.61E-03
DDIT3	-2.027629769	0.262872078	8.858173701	2.990832057	3.01E-03	2.79E-02
IER3IP1	-0.028773983	0.263015909	9.815690422	3.635749801	3.25E-04	6.09E-03
C19orf2	-2.392856585	0.263466234	11.12898247	2.85842668	4.55E-03	3.70E-02
NDUFB8	-0.020986644	0.263915909	10.2001638	3.638054763	3.22E-04	6.04E-03
MRPS21	-2.281881844	0.263992532	10.58972029	2.899262035	4.01E-03	3.41E-02
SELK	1.161412786	0.264349026	8.925145292	3.973872329	8.82E-05	2.40E-03
ELOVL1	1.348947241	0.264383442	11.05287679	4.024801175	7.19E-05	2.07E-03
ZNF101	0.462404865	0.264597403	6.9314	3.778616677	1.89E-04	4.13E-03
PSTK	-1.568657453	0.264677597	6.380132955	3.149782309	1.79E-03	1.94E-02
SIVA1	-1.840705076	0.265173377	10.25688799	3.056503835	2.44E-03	2.42E-02
PLD3	-2.415420296	0.265434416	12.70194448	2.850056229	4.67E-03	3.77E-02
HEBP2	-1.66870473	0.265617208	10.35962549	3.115782801	2.01E-03	2.10E-02
FANCE	-0.829295631	0.265800649	7.869187987	3.391167307	7.87E-04	1.11E-02
GTF3C6	1.370609227	0.266189286	9.125093994	4.030646722	7.02E-05	2.05E-03
HSP90B1	3.908062714	0.266190584	13.97462256	4.670265587	4.50E-06	3.24E-04
ZNF879	-1.017987479	0.266228247	6.810336201	3.331110689	9.71E-04	1.27E-02
MYNN	1.863062283	0.266538312	9.432252922	4.161552109	4.11E-05	1.45E-03
ACADS	-1.211939517	0.266882143	8.186431981	3.268314213	1.20E-03	1.48E-02
MEA1	0.590383318	0.267110714	10.80082614	3.81504055	1.65E-04	3.76E-03
C21orf45	1.749678963	0.267393182	8.703681656	4.131740348	4.65E-05	1.56E-03
EIF2B3	4.645231214	0.26760974	8.611491234	4.842177717	2.04E-06	1.87E-04
DAD1	-0.110424764	0.267847403	11.67159253	3.611501111	3.55E-04	6.41E-03
DNAJB9	0.809207298	0.267963312	9.197129708	3.876600699	1.30E-04	3.14E-03
SDHAF2	6.243657398	0.268078571	9.897972403	5.198224878	3.68E-07	5.72E-05
CCDC56	-0.827855706	0.268263961	9.989378084	3.391621816	7.86E-04	1.11E-02
MRI1	-2.771768718	0.268358442	9.681562338	2.714623808	7.01E-03	5.00E-02
KEAP1	1.094050435	0.268582468	11.07638929	3.955435007	9.49E-05	2.53E-03
C12orf48	-1.641927142	0.268678247	8.083828084	3.124916681	1.95E-03	2.05E-02
MRPL41	-1.996857964	0.268975	9.356658929	3.001735928	2.90E-03	2.73E-02
AHCY	0.830364508	0.269095455	12.6261724	3.882505873	1.27E-04	3.10E-03

C10orf35	-0.285568761	0.269235714	7.255347727	3.558976732	4.31E-04	7.35E-03
C13orf27	-1.823704122	0.269251299	7.043620455	3.062411046	2.39E-03	2.39E-02
GPX4	-0.508692605	0.26935	11.90756656	3.491014086	5.52E-04	8.72E-03
TBCA	-0.441977685	0.269717532	10.21611656	3.51146196	5.12E-04	8.26E-03
TMSB10	-1.105862019	0.269749675	14.43730536	3.302796727	1.07E-03	1.36E-02
GPT2	-2.461679931	0.269806494	10.11203896	2.832822107	4.92E-03	3.91E-02
TFPT	0.003068683	0.269844481	8.759120942	3.64516644	3.14E-04	5.92E-03
C19orf60	-0.612032335	0.270039286	9.168681981	3.459121163	6.19E-04	9.35E-03
CHST7	-2.70305263	0.270068831	5.727712987	2.741234104	6.48E-03	4.73E-02
DEM1	-0.361947395	0.270180195	7.461533604	3.535847326	4.69E-04	7.78E-03
LHPP	-1.440048296	0.27041526	9.285458929	3.192990411	1.55E-03	1.77E-02
INO80C	-1.324833795	0.270494805	7.762447403	3.231239948	1.37E-03	1.62E-02
PKN1	-1.615836823	0.270762987	12.28805942	3.133792154	1.89E-03	2.01E-02
PSMC3	5.681698407	0.270772403	10.87913295	5.075449592	6.71E-07	8.39E-05
C16orf61	0.414832087	0.271317532	8.047261364	3.764995507	2.00E-04	4.29E-03
RDBP	0.911088048	0.271395455	10.83179838	3.904962458	1.16E-04	2.92E-03
SHISA4	-2.58152855	0.271624026	8.635881494	2.78770294	5.64E-03	4.30E-02
ANKRD39	2.604463368	0.271978571	7.717797727	4.352009994	1.84E-05	8.46E-04
B3GALNT1	-2.243699945	0.272098377	9.641321916	2.913186664	3.84E-03	3.31E-02
LOC729678	-2.024908976	0.272111364	8.868610227	2.991797667	3.00E-03	2.78E-02
PSMA5	1.751338157	0.272185065	10.73953409	4.132177979	4.64E-05	1.56E-03
PSMG1	0.987394539	0.272209416	9.262603409	3.926083658	1.07E-04	2.75E-03
TBCE	2.081487892	0.272351299	8.603382792	4.218453889	3.24E-05	1.23E-03
RNFT1	-0.448655453	0.27241461	7.754120292	3.509420189	5.16E-04	8.32E-03
DONSON	-0.875140995	0.272504221	8.057889773	3.376666727	8.28E-04	1.15E-02
ATG4A	0.858389913	0.272505195	7.568762338	3.890315557	1.23E-04	3.06E-03
MCOLN1	1.134958828	0.272681169	8.967437987	3.966640974	9.08E-05	2.44E-03
QTRT1	-1.382404641	0.272823701	9.425673539	3.212180373	1.46E-03	1.70E-02
NDUFAB1	0.380196143	0.272907468	10.13924594	3.755050211	2.07E-04	4.41E-03
ZMPSTE24	0.487651324	0.272963636	10.67909675	3.785827231	1.84E-04	4.04E-03
SDHAF1	0.253224465	0.273041234	8.527244643	3.718385029	2.38E-04	4.85E-03
COQ3	-1.105160122	0.273092857	7.000916558	3.303023777	1.07E-03	1.36E-02
CENPQ	-0.568670921	0.273110065	7.178476461	3.472536331	5.90E-04	9.11E-03
GRN	0.765990092	0.273142208	13.37708409	3.864513044	1.36E-04	3.25E-03
MIF	-2.224731766	0.273196753	13.98798669	2.920080884	3.76E-03	3.26E-02
CLLU1OS	-2.3485415	0.273346753	0.54477987	2.874799083	4.33E-03	3.59E-02
HSPA5	4.924646742	0.273756169	13.58975601	4.905988819	1.51E-06	1.53E-04
SRPRB	2.725820302	0.274531494	9.758804708	4.382488003	1.61E-05	7.72E-04
COX7C	-0.601239984	0.274741234	10.9395388	3.462464585	6.11E-04	9.31E-03
MRPS11	1.912163086	0.274855844	9.57875	4.17440332	3.90E-05	1.40E-03
ERI3	5.381069472	0.275268182	11.1452776	5.008735623	9.26E-07	1.07E-04
ARG2	-0.496415221	0.275671429	7.548967532	3.494785328	5.44E-04	8.62E-03
MXD3	-0.299377155	0.276117532	8.467871104	3.554805434	4.38E-04	7.43E-03
ZNF136	-1.026224349	0.276188312	8.128444805	3.328466195	9.79E-04	1.28E-02
ATOX1	0.170094907	0.27670487	9.574745292	3.694200596	2.61E-04	5.20E-03

CYCS	-0.192909395	0.276851623	11.57261737	3.586852444	3.89E-04	6.84E-03
ZNF93	-2.642150742	0.276894805	7.84492987	2.764614733	6.04E-03	4.52E-02
KRT10	-0.164715529	0.277326299	9.266361201	3.595294961	3.77E-04	6.70E-03
C4orf33	-1.682116192	0.277394156	7.492142532	3.111198691	2.04E-03	2.12E-02
DYNLT1	0.311425071	0.27753539	10.04740081	3.73523208	2.24E-04	4.64E-03
CD99	-2.425148992	0.277831169	10.85303182	2.846439989	4.72E-03	3.80E-02
C1orf212	3.269107791	0.278129545	9.553788149	4.516715977	8.97E-06	5.14E-04
C1orf50	2.17290664	0.278380519	7.74812013	4.24206806	2.94E-05	1.15E-03
COMMD1	0.948850694	0.278406818	8.997207305	3.915427782	1.11E-04	2.84E-03
ZNRD1	-0.313601086	0.278420779	8.582872078	3.550503903	4.45E-04	7.50E-03
BRI3BP	-0.582429855	0.278562987	7.657594481	3.46828477	5.99E-04	9.20E-03
LSM7	0.075187339	0.278985065	9.250519805	3.666411887	2.90E-04	5.63E-03
DRAP1	3.407576471	0.279425	10.74430666	4.550373193	7.72E-06	4.68E-04
TRMT112	0.427382053	0.279468831	11.00581948	3.768593192	1.97E-04	4.25E-03
SPAG5	-2.635313653	0.279524675	9.017148052	2.767227816	6.00E-03	4.49E-02
FTL	-0.876846752	0.279580195	15.35684789	3.376126095	8.30E-04	1.15E-02
GALE	-2.253391475	0.279773701	9.237081656	2.909658203	3.88E-03	3.34E-02
CST3	-1.049988305	0.279941558	13.35871039	3.320825688	1.01E-03	1.31E-02
LOC388955	0.41280303	0.279978571	9.249358117	3.764413547	2.00E-04	4.29E-03
CENPN	0.010242135	0.280126299	8.616881981	3.647284733	3.11E-04	5.89E-03
PSMB4	4.278142363	0.280164286	12.37603019	4.757236694	3.02E-06	2.46E-04
MRPL36	-1.530045273	0.280380844	8.882486526	3.16281257	1.72E-03	1.89E-02
C7orf50	-0.266016377	0.28058474	10.00098653	3.564875533	4.22E-04	7.22E-03
C6orf1	-0.625006474	0.280717532	8.931524351	3.455097884	6.27E-04	9.45E-03
SSSCA1	0.362417611	0.280737013	9.323262013	3.749936007	2.11E-04	4.45E-03
ACN9	-0.339339969	0.280906169	8.843763474	3.542707886	4.58E-04	7.62E-03
FAM158A	-1.38839017	0.28103961	7.437662013	3.21019273	1.47E-03	1.71E-02
TSPAN15	-1.628697359	0.281327597	10.05963847	3.129420168	1.92E-03	2.04E-02
MRPS24	1.779344484	0.28143539	10.09796964	4.139558737	4.50E-05	1.53E-03
CTSC	-1.877869854	0.281515909	11.21281899	3.043552944	2.54E-03	2.49E-02
RPL37A	-2.343387169	0.281812338	14.74320942	2.87669765	4.30E-03	3.58E-02
ORMDL2	0.083408722	0.281881818	9.563928571	3.668826688	2.87E-04	5.58E-03
SERPINH1	-0.206925739	0.282032143	12.96104919	3.582648531	3.95E-04	6.91E-03
SF3B5	0.558057469	0.282040909	10.57040032	3.805870162	1.71E-04	3.84E-03
C19orf70	-1.492006882	0.28213474	9.378151786	3.175600314	1.65E-03	1.84E-02
HN1	-1.570828055	0.282180519	10.4883	3.149048311	1.80E-03	1.95E-02
COX17	-0.15875784	0.282280195	8.445021916	3.597076638	3.75E-04	6.67E-03
SLC35B1	2.55591546	0.282717208	9.662614448	4.33976481	1.94E-05	8.76E-04
FKBP2	1.772978597	0.282729221	10.25726396	4.137882108	4.53E-05	1.54E-03
C5orf34	-1.678980518	0.282888312	6.886644156	3.112271048	2.03E-03	2.11E-02
ZFPL1	8.885223359	0.283011039	10.32123994	5.746074241	2.20E-08	7.35E-06
C7orf55	-1.672570197	0.283227597	8.597875487	3.114462212	2.02E-03	2.10E-02
CYB5A	-1.869573106	0.283231494	9.452654708	3.046448613	2.52E-03	2.47E-02
MAP6D1	-2.052405937	0.283531494	6.239854708	2.982025443	3.09E-03	2.85E-02
RPL27	-2.581574489	0.283831169	13.91946234	2.787685512	5.64E-03	4.30E-02

FUNDC1	1.56893598	0.283937987	8.127927435	4.083817165	5.66E-05	1.74E-03
C20orf196	-0.685865418	0.284387013	6.920538961	3.436167527	6.71E-04	9.89E-03
LYRM4	-1.253358188	0.284435065	9.356098052	3.254758076	1.26E-03	1.52E-02
SUV39H2	0.320069837	0.284503571	8.281975812	3.737728541	2.21E-04	4.61E-03
YIF1B	-0.718906705	0.284544481	10.23391899	3.425849495	6.96E-04	1.02E-02
TMEM223	1.065937799	0.284869156	9.295873539	3.947717574	9.79E-05	2.58E-03
NDUFAF2	-1.433271738	0.284944805	7.036460065	3.195251951	1.54E-03	1.76E-02
TCEB1	1.53447119	0.285065909	9.331570617	4.074621889	5.87E-05	1.79E-03
C1orf85	1.258158221	0.285080844	10.74397029	4.000218223	7.94E-05	2.21E-03
ZNHIT1	1.479442502	0.285692208	11.08784351	4.059901539	6.24E-05	1.86E-03
EXOSC1	1.916406339	0.285785065	8.841750325	4.17551226	3.88E-05	1.39E-03
FAM118B	2.683553078	0.285840584	8.499016396	4.371894059	1.69E-05	7.97E-04
LSMD1	-0.796812067	0.28585487	9.992017695	3.401407003	7.59E-04	1.08E-02
H2AFX	-1.150102056	0.286103247	11.36361721	3.288456527	1.12E-03	1.41E-02
SKA3	-2.211900101	0.286216234	8.122380844	2.92473601	3.70E-03	3.22E-02
WDR53	1.318412334	0.286247403	7.49066461	4.016548297	7.43E-05	2.13E-03
SCAND1	1.699008715	0.286416558	10.65420893	4.118355554	4.91E-05	1.61E-03
SLC39A3	3.830441626	0.286962987	9.118369156	4.651846636	4.90E-06	3.42E-04
TMEM106C	-0.495840328	0.286967208	10.99135244	3.494961826	5.44E-04	8.62E-03
ATP6V0E1	0.842885834	0.287372078	11.62681786	3.885996873	1.25E-04	3.08E-03
HYLS1	-2.076761755	0.287473377	7.732974351	2.973344301	3.18E-03	2.91E-02
BLVRA	-0.312969258	0.287475	9.008979708	3.550695079	4.44E-04	7.50E-03
PHYH	-2.032827543	0.287706494	8.940908442	2.988986549	3.03E-03	2.80E-02
RPN1	7.898194239	0.287742857	13.47504675	5.546537195	6.29E-08	1.68E-05
S100A11	-2.627912784	0.287923052	13.49494464	2.770053733	5.95E-03	4.46E-02
GCSH	-2.512101561	0.287978571	4.308074351	2.813923568	5.21E-03	4.08E-02
C19orf50	4.652069007	0.288039935	10.84655114	4.843747831	2.02E-06	1.87E-04
LARP6	-1.628804948	0.288231494	8.726228084	3.129383569	1.92E-03	2.04E-02
SERTAD1	-1.917917256	0.288313312	8.284016396	3.029539335	2.66E-03	2.57E-02
NHP2	0.530967844	0.288399351	10.98309448	3.798169758	1.76E-04	3.92E-03
EBP	-2.039311915	0.28841461	9.598648864	2.98668272	3.05E-03	2.82E-02
HYOU1	2.204366469	0.288425649	12.26719205	4.250167491	2.84E-05	1.13E-03
PDIA5	-1.774003565	0.288541883	9.728394318	3.07961917	2.26E-03	2.29E-02
SEC61B	2.050768116	0.288701299	10.3032526	4.210492389	3.35E-05	1.25E-03
MAPK13	-2.512527417	0.289144805	9.231557468	2.813763441	5.21E-03	4.08E-02
E2F8	-2.623011238	0.289380519	8.074752597	2.771923812	5.91E-03	4.44E-02
TOMM5	1.265251654	0.289411039	10.58471851	4.00214379	7.87E-05	2.20E-03
RPN2	7.837028095	0.289650325	13.00971737	5.533981223	6.71E-08	1.70E-05
TMEM206	0.025541925	0.289678571	7.754515909	3.65179895	3.06E-04	5.82E-03
ZNF20	-0.094287321	0.290246753	7.498118831	3.616305416	3.49E-04	6.34E-03
IMPA2	-1.713024202	0.290294156	9.336252922	3.100609991	2.11E-03	2.17E-02
ARF5	2.571392643	0.290484416	11.53840519	4.343671902	1.91E-05	8.66E-04
FAM58A	0.796245215	0.290531169	8.228368831	3.872978843	1.31E-04	3.17E-03
PRKACA	2.54204364	0.290818182	11.43303701	4.336260347	1.97E-05	8.84E-04
C17orf53	-1.079825501	0.290834091	7.530038474	3.311209361	1.04E-03	1.33E-02

BNIP1	2.424258762	0.291119481	7.272335714	4.306402947	2.24E-05	9.42E-04
CRELD2	-0.775585981	0.291203571	8.617192045	3.408082663	7.41E-04	1.06E-02
TMEM180	-2.300525977	0.291203896	6.716474026	2.892439661	4.10E-03	3.46E-02
TMEM117	-2.368973411	0.291308442	6.997537987	2.867261413	4.43E-03	3.63E-02
MRPL17	2.926968711	0.291541558	9.566938961	4.432599484	1.30E-05	6.56E-04
ATP5H	-0.194515342	0.291680195	10.53076672	3.586371003	3.90E-04	6.84E-03
SOD1	1.728596535	0.291978247	11.60798003	4.126176023	4.75E-05	1.58E-03
OST4	0.291421518	0.292062987	12.72455487	3.729449562	2.28E-04	4.71E-03
CASP6	0.45007721	0.292380195	8.640695942	3.775091275	1.92E-04	4.17E-03
MED8	6.106976443	0.292787013	9.64969026	5.16858761	4.26E-07	6.33E-05
CDC6	-1.632216714	0.292858766	8.197443019	3.128222754	1.93E-03	2.04E-02
PSMA7	2.875514063	0.293229221	11.60224838	4.419828168	1.37E-05	6.85E-04
ACOT6	-2.539748277	0.293252922	1.570861526	2.80351005	5.38E-03	4.17E-02
TSSK6	-1.584345765	0.293298377	5.659574513	3.144473629	1.83E-03	1.97E-02
DPM3	-2.584245399	0.293836039	9.320195292	2.786672104	5.66E-03	4.31E-02
LYPLA1	2.651858815	0.295013636	10.83753279	4.363935321	1.75E-05	8.19E-04
PPCS	2.248664772	0.295262338	10.32307857	4.261549124	2.70E-05	1.09E-03
ETV2	-2.223298265	0.295275	4.778529708	2.920601283	3.75E-03	3.25E-02
GPX1	0.635118222	0.295382468	12.89026656	3.827698355	1.57E-04	3.62E-03
ACTL6A	4.123366359	0.296180844	10.76895666	4.72103103	3.57E-06	2.76E-04
NDUFB6	-0.034177459	0.296191883	9.660465422	3.63414965	3.27E-04	6.10E-03
FBXO22OS	-1.445052316	0.296249026	5.119364773	3.191319459	1.56E-03	1.78E-02
STXBP2	0.796008229	0.296474351	10.97683263	3.872912596	1.31E-04	3.17E-03
SAC3D1	2.158796147	0.297002597	8.514112338	4.238430818	2.98E-05	1.16E-03
BOLA1	-0.734401696	0.297125325	8.190300325	3.421000865	7.08E-04	1.03E-02
FAU	0.507813155	0.297212338	12.73898344	3.791576641	1.80E-04	3.97E-03
ARL2	0.420159352	0.29741461	10.58792808	3.76652305	1.98E-04	4.27E-03
OIP5	-1.462983774	0.297425974	6.763335065	3.185325032	1.59E-03	1.80E-02
COX7B	-2.019883374	0.29765974	11.37489091	2.993580484	2.98E-03	2.77E-02
CSRP3	-1.239105331	0.297778896	0.485546591	3.259428892	1.24E-03	1.50E-02
GOT1	1.731775032	0.297853247	9.933444156	4.127015358	4.74E-05	1.57E-03
NDUFA12	3.006589379	0.298391234	9.909907955	4.452298515	1.19E-05	6.23E-04
RAB11B	2.44754159	0.298517857	12.14435828	4.312319382	2.18E-05	9.30E-04
ATP5EP2	-2.326838482	0.298599026	4.989530032	2.882785265	4.22E-03	3.54E-02
HAUS1	-0.45887203	0.298611688	8.007272727	3.506294286	5.22E-04	8.36E-03
TOR3A	4.656023532	0.298762013	9.816925162	4.844655682	2.02E-06	1.87E-04
SAAL1	2.721100781	0.298860065	7.449537825	4.381306209	1.62E-05	7.74E-04
CARM1	2.437497222	0.29887987	11.41660682	4.309767871	2.20E-05	9.33E-04
OSTC	2.800337051	0.299330844	10.57457127	4.401110474	1.49E-05	7.23E-04
PRR11	-1.606510505	0.299535714	7.37326461	3.136959101	1.87E-03	2.00E-02
MAGOH	2.183492883	0.299547078	9.536176136	4.244795049	2.90E-05	1.14E-03
PRAF2	-1.153318061	0.299681169	10.60241526	3.287411807	1.13E-03	1.41E-02
RPS6KB2	5.688453241	0.299690909	10.29379545	5.076940113	6.66E-07	8.39E-05
DDOST	10.13306256	0.300121104	12.73825536	5.990690422	5.84E-09	2.46E-06
SIL1	1.61023931	0.300227597	9.333341071	4.09481267	5.41E-05	1.69E-03

PRELID1	1.564012843	0.300248377	11.2660112	4.082504795	5.69E-05	1.75E-03
YBX1	1.492853886	0.300297727	13.66125925	4.063493522	6.15E-05	1.85E-03
C6orf226	-2.58849754	0.300621104	6.617885877	2.785058016	5.68E-03	4.32E-02
CHCHD1	2.255877397	0.300681169	9.261633442	4.26339973	2.68E-05	1.09E-03
ATP5E	1.348752611	0.300751299	11.34361916	4.024748619	7.19E-05	2.07E-03
C6orf153	3.774512224	0.300807468	9.590884253	4.638535835	5.20E-06	3.58E-04
FAM32A	4.803951633	0.301017857	10.96477711	4.878512433	1.72E-06	1.69E-04
SRA1	3.340328439	0.301762338	9.717271429	4.534054546	8.30E-06	4.92E-04
ATP1B3	1.111345947	0.302311364	10.76072192	3.960176218	9.31E-05	2.49E-03
PSENEN	-0.463732561	0.302472403	11.05506088	3.504806239	5.25E-04	8.40E-03
MAGOHB	0.545690102	0.302959091	8.533097078	3.802356411	1.73E-04	3.87E-03
NR2C2AP	0.287196474	0.303	9.166913636	3.728227165	2.30E-04	4.73E-03
ERH	1.642592856	0.303292857	11.02362305	4.103407241	5.22E-05	1.65E-03
CLPP	1.714521317	0.303572078	9.967178896	4.122457398	4.83E-05	1.59E-03
DPCD	0.510201084	0.303615584	8.856036364	3.792257066	1.80E-04	3.97E-03
ARMC6	3.692039137	0.303666883	9.804382143	4.618847346	5.68E-06	3.84E-04
C7orf68	1.210507536	0.303684416	8.647444156	3.987261442	8.36E-05	2.31E-03
PCNA	0.544342825	0.303727273	11.31083961	3.801973452	1.73E-04	3.87E-03
KRTCAP2	2.386561609	0.303794156	11.29260942	4.29680843	2.33E-05	9.74E-04
RRM2	-2.531264494	0.30382013	9.859748377	2.80670948	5.32E-03	4.14E-02
POP7	5.264253645	0.303879221	9.422494156	4.982609621	1.05E-06	1.19E-04
C11orf82	-1.478196088	0.304086688	8.026060877	3.180231335	1.62E-03	1.82E-02
IQCG	-2.694751835	0.304186364	8.616458766	2.744431974	6.42E-03	4.70E-02
FBXO16	-2.703293042	0.304403896	6.719202597	2.741141433	6.48E-03	4.73E-02
HYAL3	-2.148611852	0.304778571	7.417104221	2.947594455	3.45E-03	3.08E-02
POLE2	-2.22220109	0.304854545	6.540327273	2.920999528	3.75E-03	3.25E-02
ZFAND2A	-0.523204215	0.30531526	7.734068669	3.486551712	5.61E-04	8.79E-03
TMEM14A	2.053041036	0.305443182	9.15885211	4.211081909	3.34E-05	1.25E-03
SUB1	3.010870831	0.305473377	11.85228604	4.453355633	1.18E-05	6.23E-04
HAX1	5.343915428	0.305715584	11.12968636	5.000438575	9.63E-07	1.11E-04
GTPBP8	3.509179977	0.305736039	7.493048539	4.574932642	6.92E-06	4.35E-04
SSR2	4.155347025	0.305844805	12.70891396	4.728531561	3.45E-06	2.69E-04
NDUFAF4	0.848874174	0.306976299	8.791992695	3.887665447	1.24E-04	3.06E-03
NDUFV2	3.00214855	0.307153896	10.08844838	4.451201813	1.20E-05	6.24E-04
UCHL3	2.219248383	0.307457143	8.377992208	4.25399413	2.79E-05	1.12E-03
MRPL14	1.885412583	0.30756039	10.09021981	4.167406249	4.01E-05	1.43E-03
ZNF414	1.68506696	0.308496753	9.183138636	4.114665963	4.99E-05	1.61E-03
LACTB2	-1.050892143	0.308602273	9.23127776	3.320534767	1.01E-03	1.31E-02
UQCR11	-0.049730894	0.308773052	10.37725276	3.629540151	3.32E-04	6.17E-03
AIMP2	2.49016138	0.308965584	9.366730195	4.323131061	2.08E-05	9.07E-04
PPIB	2.758707184	0.309326299	13.12800536	4.390715306	1.56E-05	7.46E-04
CMC1	0.014757053	0.309577273	8.105106818	3.648617394	3.10E-04	5.87E-03
NPC2	-0.92417709	0.309831169	12.52952532	3.361092844	8.74E-04	1.19E-02
MYCBP	0.257996186	0.310174026	9.787625974	3.71976889	2.37E-04	4.83E-03
C14orf156	2.131555846	0.310374026	9.34252987	4.231401293	3.07E-05	1.18E-03

ALG3	4.702895327	0.310868506	11.34731997	4.855405143	1.92E-06	1.81E-04
CTSL1	1.104317279	0.31096039	10.83757565	3.958250068	9.39E-05	2.51E-03
PPAN	1.085968377	0.311026299	9.383038474	3.953217723	9.58E-05	2.54E-03
FADS3	2.576051553	0.311053896	9.630623052	4.3448474	1.90E-05	8.65E-04
DCTPP1	0.958343783	0.311167532	10.67948117	3.918054677	1.10E-04	2.82E-03
NUF2	-2.135859121	0.311443506	8.311621104	2.95218027	3.40E-03	3.06E-02
HBXIP	3.479080983	0.311498377	10.85489594	4.567669115	7.15E-06	4.45E-04
ZWINT	-0.908291827	0.311687013	9.313172727	3.366145318	8.59E-04	1.18E-02
SHFM1	3.537169243	0.311928247	10.51416737	4.581678078	6.72E-06	4.25E-04
TSEN15	4.813351652	0.312026299	9.835001461	4.880657082	1.70E-06	1.68E-04
HOOK2	-1.132850102	0.312427922	10.03217305	3.294055568	1.10E-03	1.39E-02
MGST3	0.206736666	0.312991234	10.69933847	3.704878319	2.51E-04	5.04E-03
JTB	6.457030196	0.313040909	11.89461591	5.244212649	2.93E-07	4.85E-05
PPP4C	7.905147382	0.3133	11.44246169	5.547963052	6.24E-08	1.68E-05
NDUFA9	1.984516112	0.313634416	10.63995097	4.193276536	3.60E-05	1.31E-03
ATP5J	1.5637468	0.313753247	11.44527662	4.082433865	5.69E-05	1.75E-03
P4HB	4.151491879	0.31385487	13.87126055	4.727627943	3.47E-06	2.69E-04
UXT	0.070829911	0.313945455	10.63965519	3.665131426	2.91E-04	5.63E-03
SLC25A33	1.053011261	0.314634416	8.06721526	3.944164442	9.93E-05	2.61E-03
TP53TG1	-0.403729745	0.314811688	7.519628571	3.523135537	4.91E-04	8.00E-03
BCL2L12	2.24474701	0.314837013	8.962610065	4.260543612	2.72E-05	1.09E-03
LOC440957	-2.039108222	0.314931818	7.354437338	2.986755116	3.05E-03	2.82E-02
FOXM1	-2.010159769	0.315190584	11.01405633	2.997027067	2.95E-03	2.75E-02
SCNM1	4.485066207	0.315259416	9.497552435	4.80527496	2.42E-06	2.09E-04
IFI27L1	-2.072792383	0.31545974	6.846253896	2.974760732	3.16E-03	2.90E-02
ORC6L	-0.918531491	0.315833117	7.807917857	3.362889292	8.69E-04	1.18E-02
APOO	1.77448296	0.316160065	8.189437825	4.138278377	4.52E-05	1.54E-03
ATPIF1	1.685691293	0.316331169	11.20818896	4.114831251	4.98E-05	1.61E-03
MRPL2	2.631739932	0.316523052	9.919939448	4.358876647	1.79E-05	8.31E-04
PSEN2	4.759448198	0.316587013	8.354234416	4.868347897	1.80E-06	1.75E-04
SSBP1	4.156795604	0.316723701	10.38863782	4.728871059	3.45E-06	2.69E-04
NMB	-2.54929367	0.316907143	7.492453571	2.799906116	5.44E-03	4.20E-02
C2orf7	-1.50287627	0.317010065	8.272886201	3.17195116	1.67E-03	1.85E-02
NUCB2	0.426878143	0.317390909	9.643501299	3.768448796	1.97E-04	4.25E-03
ZNF653	2.00537191	0.317478247	7.707254708	4.198702757	3.52E-05	1.29E-03
POC1A	0.337708288	0.317532143	8.385446591	3.742817526	2.17E-04	4.54E-03
NXT1	4.0978626	0.317845779	9.430977435	4.715042198	3.67E-06	2.79E-04
RAD54L	-1.930704449	0.31866039	8.222897078	3.025051919	2.70E-03	2.59E-02
TM7SF2	-1.98089504	0.318730519	10.18132175	3.00737767	2.85E-03	2.69E-02
NOP10	0.430214977	0.319247078	10.28074821	3.769404871	1.96E-04	4.24E-03
CDC45	-2.658600799	0.319438961	7.659117532	2.758318013	6.16E-03	4.57E-02
C21orf119	-0.429756649	0.32044026	7.157313636	3.515195795	5.06E-04	8.17E-03
CETN4P	-2.732040313	0.320850325	1.491922565	2.730038711	6.70E-03	4.83E-02
C11orf31	3.116971517	0.321253247	10.28785195	4.479482888	1.06E-05	5.72E-04
TMEM125	-0.154560785	0.321310714	9.506726136	3.598331303	3.73E-04	6.66E-03

C17orf61	-2.063051723	0.321873701	8.776765422	2.978233899	3.13E-03	2.88E-02
GYG1	2.389364085	0.321971429	10.00951039	4.297522355	2.32E-05	9.73E-04
CDK5	3.471143451	0.321980195	8.757353084	4.565751947	7.21E-06	4.47E-04
RAD51	-0.208074466	0.321992532	7.776297565	3.582303793	3.96E-04	6.91E-03
IER2	-2.166960692	0.322040584	11.68134172	2.940984478	3.52E-03	3.13E-02
SEPHS2	6.702785779	0.322629545	10.53890828	5.296768667	2.25E-07	4.16E-05
ARPM1	-0.592284128	0.322915909	6.348807955	3.465236809	6.05E-04	9.27E-03
RNF5	3.680069783	0.322957143	11.21527078	4.615983911	5.76E-06	3.87E-04
NUDT5	3.276906577	0.323395779	9.924062825	4.518617405	8.89E-06	5.14E-04
RPL26L1	1.751425357	0.323822078	7.508716234	4.132200978	4.64E-05	1.56E-03
TXNDC17	2.484077781	0.323953247	9.269378571	4.321589248	2.09E-05	9.07E-04
EIF3G	1.159903888	0.324087013	11.7198026	3.97346018	8.83E-05	2.40E-03
ALKBH7	-1.084880474	0.32431461	10.3099664	3.309577608	1.05E-03	1.34E-02
FAHD2B	-2.209816789	0.324368506	7.181660227	2.925491143	3.70E-03	3.22E-02
SEPP1	-2.744183345	0.324798377	12.55287646	2.72533593	6.79E-03	4.88E-02
C11orf51	1.446126622	0.324889935	9.20842224	4.050966188	6.47E-05	1.92E-03
EPCAM	-1.649320538	0.324900649	12.76319448	3.122397282	1.96E-03	2.07E-02
CCDC23	1.406760928	0.325133766	8.595924675	4.040385479	6.75E-05	1.98E-03
CEP55	-1.913435883	0.325551623	9.309103734	3.031110507	2.64E-03	2.57E-02
JDP2	2.828241509	0.32560487	9.648101136	4.408066304	1.44E-05	7.11E-04
PPIL5	1.44186935	0.325653247	7.550029221	4.049823112	6.50E-05	1.92E-03
LOC145783	-0.782303632	0.325674351	5.770706656	3.405971253	7.47E-04	1.07E-02
FAM125A	1.059941427	0.326206818	10.21802224	3.946069702	9.85E-05	2.59E-03
CHCHD2	2.836812182	0.327319805	12.08335211	4.41020081	1.43E-05	7.08E-04
TBC1D7	1.974895091	0.327515909	8.817326786	4.190771255	3.64E-05	1.32E-03
PDCD10	3.673427973	0.327748701	10.08720812	4.614394323	5.80E-06	3.89E-04
GSTO1	2.189552038	0.32834026	9.834485714	4.246355173	2.88E-05	1.14E-03
BUB1	-1.308607585	0.328677597	9.55126737	3.236592941	1.34E-03	1.60E-02
LSM2	1.934371514	0.328713961	10.26517062	4.180204419	3.80E-05	1.37E-03
ZNF799	-2.762304244	0.329662987	5.798997078	2.718303637	6.93E-03	4.96E-02
LOC388789	1.57966829	0.329830844	9.372616071	4.086676792	5.59E-05	1.73E-03
OAZ3	-0.369279632	0.329928896	3.880104058	3.533619621	4.73E-04	7.83E-03
SYNGR2	-0.513945819	0.330182468	12.41008734	3.48939931	5.55E-04	8.74E-03
HIST1H2AL	-2.705585236	0.330294481	1.936812175	2.740257715	6.50E-03	4.74E-02
ZNF90	-2.012048747	0.330350649	9.895627922	2.9963578	2.95E-03	2.76E-02
ATP5G3	1.969668334	0.330409416	12.11518198	4.189409668	3.66E-05	1.33E-03
SLC25A5	1.50800362	0.330425649	12.68146347	4.067547676	6.04E-05	1.83E-03
PRR19	0.934859982	0.331382792	6.504438149	3.911553421	1.13E-04	2.87E-03
FBXO6	-1.425583649	0.331485714	8.404441558	3.197815889	1.53E-03	1.75E-02
POLR2J	4.052440113	0.331618506	10.63001769	4.704359779	3.86E-06	2.91E-04
S100A13	-1.675092926	0.331629221	11.19409123	3.11360007	2.02E-03	2.11E-02
FBXL12	4.102710218	0.331762987	9.028958117	4.716181027	3.65E-06	2.79E-04
CC2D1A	0.370473342	0.332283766	10.56173604	3.752254118	2.09E-04	4.43E-03
STMN1	-1.143577257	0.332311364	12.19509529	3.290575161	1.12E-03	1.40E-02
C3orf21	0.40758734	0.332472403	9.683160877	3.762917241	2.01E-04	4.31E-03

NENF	-0.435314348	0.332659091	10.01355292	3.51349823	5.09E-04	8.22E-03
RPS26	-2.2291954	0.332707792	10.59510974	2.918459905	3.78E-03	3.27E-02
SPC25	-1.972151277	0.333175974	7.759607468	3.010463742	2.82E-03	2.67E-02
ECT2	-0.247876667	0.33357013	10.61398961	3.570340142	4.14E-04	7.11E-03
THOC4	1.808708	0.33515974	10.24411364	4.147284601	4.36E-05	1.50E-03
PSMG3	3.264744656	0.335421104	9.026241721	4.515651893	9.01E-06	5.15E-04
PLSCR1	-1.831061412	0.335525325	11.08249123	3.059855971	2.41E-03	2.41E-02
HIGD2A	0.385691437	0.33572987	11.31337338	3.756629716	2.06E-04	4.39E-03
NDUFC1	1.088100225	0.336043506	9.639956169	3.953802697	9.55E-05	2.54E-03
APOA1BP	4.226591211	0.336056494	11.68191721	4.745204049	3.20E-06	2.54E-04
TIMM8B	-0.034569689	0.33634026	9.42232013	3.634033472	3.27E-04	6.10E-03
IL20RB	-2.309507121	0.336349351	4.400426623	2.889147792	4.14E-03	3.49E-02
NCAPH	-1.401138204	0.336952597	8.718189286	3.2059556	1.49E-03	1.73E-02
FAM195A	0.305564932	0.337093506	9.241818182	3.733538907	2.25E-04	4.66E-03
MGST2	1.375936333	0.337549351	9.849223377	4.032083093	6.98E-05	2.04E-03
MRPL52	1.278309983	0.337670779	9.395287987	4.005686402	7.76E-05	2.19E-03
RABAC1	0.571021325	0.338047727	10.81060763	3.809550229	1.68E-04	3.80E-03
BOLA3	2.516128065	0.338646429	8.378736851	4.329706555	2.02E-05	8.91E-04
TIMM10	3.239838945	0.338674026	8.743332468	4.509573689	9.25E-06	5.26E-04
FAM162A	2.674667049	0.338734416	10.55916916	4.369663976	1.70E-05	8.03E-04
PGLS	1.592824916	0.339831818	11.32021266	4.090179942	5.51E-05	1.71E-03
PLP2	-2.156514174	0.340075325	11.50616948	2.944749443	3.48E-03	3.10E-02
MRPL15	4.991958846	0.340362013	10.59330828	4.921255842	1.41E-06	1.44E-04
SILV	-1.535193303	0.340423052	5.694379058	3.161078192	1.73E-03	1.89E-02
CENPK	-1.220776502	0.340555844	7.153396753	3.265426305	1.22E-03	1.49E-02
UBE2L6	-1.620043348	0.340829221	11.54394318	3.132362759	1.90E-03	2.02E-02
TRMT1	0.311918405	0.341878571	9.870889935	3.735374587	2.23E-04	4.64E-03
YIF1A	4.431345181	0.342347403	10.80834318	4.792843029	2.57E-06	2.17E-04
HSPE1	3.500429829	0.342378247	11.09908653	4.572822078	6.99E-06	4.37E-04
TPX2	-1.343035418	0.342600649	11.17040747	3.225225417	1.39E-03	1.64E-02
CDK1	0.090088147	0.342787987	10.08152386	3.670787513	2.85E-04	5.56E-03
FAM176B	-1.123253759	0.342797727	9.073945617	3.297166191	1.09E-03	1.38E-02
SLC25A39	7.048832089	0.34311461	11.99271964	5.370050322	1.56E-07	3.33E-05
BOLA2	3.994670687	0.343294481	10.5555888	4.690743372	4.10E-06	3.04E-04
FKBPL	5.51958304	0.343707468	7.561226461	5.039566163	7.98E-07	9.53E-05
PPIL1	4.244839879	0.343724675	9.918821429	4.749466494	3.13E-06	2.52E-04
MAP4K2	3.211387425	0.343778896	8.649806981	4.502621387	9.54E-06	5.37E-04
RPP21	3.908578271	0.343949026	9.251862825	4.670387715	4.50E-06	3.24E-04
DGAT2	-2.58071138	0.344201623	7.968204708	2.788012922	5.63E-03	4.30E-02
TMEM161A	3.900138476	0.344646429	9.79622711	4.668388095	4.54E-06	3.25E-04
MRPL12	1.127156754	0.344923701	10.66633458	3.964505964	9.15E-05	2.45E-03
LPAR2	3.007720928	0.345018506	9.324501461	4.452577923	1.19E-05	6.23E-04
MLF1IP	-1.089436672	0.345129545	8.325906331	3.308106219	1.05E-03	1.34E-02
C2orf79	0.617157945	0.345251623	9.631669318	3.822621032	1.60E-04	3.68E-03
CKS1B	1.271094947	0.345537987	10.29399172	4.003729373	7.82E-05	2.20E-03

RAG1AP1	2.798101793	0.345540909	10.45098539	4.400552868	1.49E-05	7.23E-04
GNG5	6.834282679	0.345916883	11.32747922	5.324713498	1.96E-07	3.83E-05
NDUFS3	7.810073452	0.346131169	10.77963766	5.528440719	6.91E-08	1.70E-05
OCEL1	1.269361609	0.346325974	8.561058442	4.003259089	7.84E-05	2.20E-03
NDUFB2	2.24891193	0.346328571	10.27314286	4.261612551	2.70E-05	1.09E-03
BAK1	3.322405495	0.346519156	9.770494643	4.52969667	8.47E-06	4.99E-04
CSTB	1.853509531	0.346577922	11.70488442	4.159047753	4.15E-05	1.46E-03
EXO1	-0.797155795	0.347622727	7.787088636	3.4012988	7.59E-04	1.08E-02
ISOC2	1.20795994	0.347673052	10.12978328	3.986567656	8.38E-05	2.31E-03
NPM3	0.278827864	0.347687662	8.468029545	3.725804867	2.32E-04	4.76E-03
SEC11C	0.916791816	0.347963961	8.824926786	3.906544789	1.15E-04	2.91E-03
PEPD	2.623863837	0.34801526	10.58409984	4.356894881	1.80E-05	8.35E-04
PRDX5	6.198524122	0.348052922	11.94330179	5.188453989	3.86E-07	5.91E-05
PDCD2L	3.574052416	0.348176623	8.223903896	4.590553817	6.45E-06	4.13E-04
DBI	1.452009329	0.348275649	10.25986445	4.052545217	6.42E-05	1.91E-03
LOC728554	-1.544936353	0.348426948	9.129025162	3.157793307	1.75E-03	1.91E-02
ANAPC11	1.804963198	0.348480519	10.44337987	4.146300016	4.38E-05	1.50E-03
SLC2A1	-0.869935498	0.348543831	12.86048231	3.37831609	8.23E-04	1.14E-02
RPS10	0.62009223	0.348994156	12.80579513	3.823450964	1.59E-04	3.68E-03
ZNF433	-0.077781287	0.349667208	6.702962175	3.621213417	3.43E-04	6.28E-03
NDUFB4	4.65813724	0.349706494	11.43977597	4.845140873	2.01E-06	1.87E-04
PSMD8	3.142523388	0.35027987	12.12132175	4.48575516	1.03E-05	5.64E-04
RAD51AP1	-0.148421006	0.350654545	8.556161688	3.600166003	3.71E-04	6.63E-03
NDUFS6	1.612713772	0.351313636	10.28831136	4.095470568	5.39E-05	1.69E-03
ZNF627	3.612035201	0.352521429	9.411694481	4.599678646	6.19E-06	4.02E-04
BRP44	5.815671914	0.35295	9.681961364	5.104943928	5.81E-07	7.80E-05
ADA	1.728230876	0.353749675	7.7040625	4.126079455	4.76E-05	1.58E-03
TEKT5	-2.549346902	0.353982792	1.477923864	2.799886006	5.44E-03	4.20E-02
CYBA	-0.601427114	0.354711688	12.2042487	3.462406638	6.11E-04	9.31E-03
EDEM2	8.620249725	0.354996429	10.10739692	5.693060247	2.92E-08	9.35E-06
PLK1	-0.0399887	0.355109416	10.25198977	3.632428025	3.29E-04	6.11E-03
SNRPG	3.792676894	0.355729545	9.137292045	4.642862514	5.10E-06	3.53E-04
CCDC130	2.35700791	0.355994805	9.438005844	4.289273307	2.40E-05	9.95E-04
TMEM147	5.202090384	0.356002273	10.94923685	4.968659441	1.12E-06	1.25E-04
GMNN	0.207017944	0.356239935	8.689616721	3.704960177	2.51E-04	5.04E-03
C17orf37	-0.020416354	0.356648052	10.09784221	3.638223509	3.22E-04	6.04E-03
RPL18A	-2.159203026	0.356880195	10.03668555	2.943780805	3.49E-03	3.10E-02
CCDC58	5.62048154	0.356880195	8.553442695	5.061924676	7.16E-07	8.75E-05
MRPL22	4.596508525	0.357043182	9.207616396	4.830977268	2.15E-06	1.90E-04
HINT1	2.126564145	0.357051948	11.77079221	4.230112032	3.09E-05	1.19E-03
KIF18A	0.183192754	0.357705844	7.909469805	3.698020649	2.57E-04	5.15E-03
RPA3	2.282600836	0.358706169	8.434945942	4.270250214	2.61E-05	1.07E-03
GLRX3	6.540544183	0.358750974	9.956837175	5.262121285	2.68E-07	4.69E-05
C1orf31	3.654533982	0.358809091	8.73932013	4.60986982	5.92E-06	3.89E-04
TIMM44	5.410277092	0.359495779	9.413761526	5.015249984	8.97E-07	1.05E-04

C11orf83	3.611187916	0.359580844	9.049244968	4.599475269	6.20E-06	4.02E-04
CDK2AP2	2.94616076	0.360496753	11.36116916	4.437354808	1.27E-05	6.47E-04
GINS2	-0.609615984	0.360635065	7.932759091	3.459869997	6.17E-04	9.34E-03
MYEOV2	1.743805982	0.36082987	9.463666234	4.130190953	4.68E-05	1.56E-03
NDUFA1	0.688802948	0.361551299	10.97039058	3.842838655	1.48E-04	3.48E-03
COX6A1	3.001264277	0.361904221	11.50598847	4.450983406	1.20E-05	6.24E-04
COX8A	3.133608579	0.362062662	12.10755081	4.483567693	1.04E-05	5.68E-04
PSMB8	-1.954463414	0.362506818	10.86942808	3.016697513	2.77E-03	2.64E-02
PSMB3	4.724969819	0.362822727	11.24474383	4.86046063	1.87E-06	1.78E-04
ATP6V1F	8.57019499	0.363002273	11.20890244	5.683001199	3.08E-08	9.57E-06
UCP1	-2.681549201	0.363031818	1.013184091	2.749510986	6.32E-03	4.65E-02
CENPL	5.791977908	0.363860714	8.160668019	5.099738097	5.96E-07	7.85E-05
PODXL2	-2.539978573	0.363977597	9.67059789	2.803423151	5.38E-03	4.17E-02
ATP5O	4.745171239	0.364009416	11.57614886	4.865083224	1.83E-06	1.76E-04
NUDT1	2.342309506	0.364809416	7.734157305	4.285521391	2.44E-05	1.01E-03
KIF20A	-1.130111053	0.364819481	9.668495455	3.2949437	1.10E-03	1.38E-02
KCNK17	-2.404383796	0.365639935	3.105163474	2.854153338	4.61E-03	3.74E-02
MSX2P1	-1.972135082	0.36577987	0.966096429	3.010469456	2.82E-03	2.67E-02
RFC4	3.597462191	0.366462338	9.446901299	4.596179542	6.29E-06	4.05E-04
C1orf122	2.140919081	0.366510714	10.24575925	4.233818699	3.04E-05	1.17E-03
ZNF626	-2.45026048	0.366641558	8.326861688	2.837085663	4.86E-03	3.87E-02
SNRPB	4.674904477	0.366778247	12.40602744	4.848988235	1.97E-06	1.86E-04
PRDX4	3.090047291	0.366918182	11.01419286	4.472865433	1.09E-05	5.87E-04
NEIL3	-1.701012892	0.366966558	6.442709903	3.104728937	2.08E-03	2.15E-02
CDCA3	0.280953557	0.367347403	8.85176461	3.726420286	2.31E-04	4.75E-03
UBTD1	3.12208991	0.367904545	9.557891234	4.480739925	1.05E-05	5.72E-04
DEPDC1	-1.692875328	0.367910065	8.678339448	3.107516581	2.06E-03	2.14E-02
ROPN1	-0.706491085	0.368117208	0.964721591	3.429729969	6.87E-04	1.01E-02
LOC730101	-2.653363909	0.368272727	6.913938961	2.76032406	6.12E-03	4.55E-02
AURKA	0.855198182	0.368518506	9.387391071	3.88942685	1.23E-04	3.06E-03
TIGD3	-2.700066204	0.368671104	3.324449188	2.742385029	6.46E-03	4.72E-02
CUTA	6.966299039	0.368907468	11.95569529	5.352647543	1.70E-07	3.45E-05
C19orf42	6.178010309	0.369095455	10.44454448	5.18400789	3.94E-07	6.00E-05
TROAP	-1.21649814	0.369627597	9.343898214	3.266824761	1.21E-03	1.48E-02
ZNF709	-0.967232005	0.369932792	7.744938474	3.347363248	9.17E-04	1.23E-02
ICAM3	2.211803837	0.370177922	8.475348052	4.252080269	2.81E-05	1.12E-03
LOC729991	3.910611278	0.370270779	8.69443539	4.67086928	4.49E-06	3.24E-04
NDUFB3	6.354985315	0.370874351	9.736337825	5.222261135	3.26E-07	5.28E-05
SEC61G	5.632130819	0.372085714	9.673903896	5.064500741	7.07E-07	8.70E-05
NDUFC2	-0.077533813	0.372217857	12.22070698	3.621286956	3.43E-04	6.28E-03
PNP	-0.573162063	0.372362662	9.71428263	3.47114908	5.92E-04	9.13E-03
ACBD7	-2.266754304	0.372652273	8.006743019	2.904786469	3.94E-03	3.37E-02
SAR1B	4.624710861	0.372751299	8.725522403	4.837463163	2.08E-06	1.88E-04
COX5A	1.505985613	0.372827922	11.28470682	4.067007855	6.06E-05	1.83E-03
E2F1	0.494768834	0.37287987	9.652151623	3.787857787	1.83E-04	4.02E-03

COX7A2	1.697288593	0.373649026	11.1779901	4.117900495	4.92E-05	1.61E-03
GSTP1	6.305466125	0.373834091	13.48731769	5.211581079	3.44E-07	5.46E-05
C1orf210	-1.422657043	0.374718831	9.003420455	3.198791393	1.52E-03	1.75E-02
MAD2L1	0.657264505	0.374804221	8.816570942	3.833950665	1.53E-04	3.57E-03
GPX7	0.27010312	0.374967857	9.182550812	3.723277958	2.34E-04	4.79E-03
MRPS6	3.168218895	0.375050974	10.19888068	4.492054982	1.00E-05	5.53E-04
DNAJB1	5.636872753	0.376471104	12.13412192	5.065549034	7.04E-07	8.70E-05
NDUFB1	2.95690627	0.377354221	9.047102435	4.440015338	1.26E-05	6.43E-04
NDUFS8	5.954544918	0.377371104	11.85100698	5.135366912	5.01E-07	7.00E-05
NDUFB5	5.010242144	0.377669805	11.10577841	4.925395728	1.38E-06	1.42E-04
NME1	2.835785207	0.378576623	11.00949416	4.409945092	1.43E-05	7.08E-04
C19orf12	0.7222422	0.378709091	9.676087662	3.852242044	1.42E-04	3.36E-03
TIMP1	-0.654913625	0.379812013	13.14893588	3.445807171	6.49E-04	9.66E-03
C10orf125	-0.577239049	0.380020455	7.322546591	3.469889313	5.95E-04	9.17E-03
SNRPF	5.248998476	0.380466234	9.662142857	4.979189238	1.07E-06	1.20E-04
C6orf115	1.874847493	0.381061039	9.047503247	4.164639882	4.06E-05	1.44E-03
CDCA5	1.807900118	0.381093506	9.467813636	4.14707221	4.36E-05	1.50E-03
DIAPH3	-1.444721731	0.381129221	7.559493831	3.191429874	1.56E-03	1.78E-02
SNRPC	7.594288821	0.381547727	10.62253295	5.483922446	8.70E-08	2.05E-05
AP1M2	-0.920822316	0.381975	11.1287849	3.362160451	8.71E-04	1.19E-02
CHAC2	1.506432576	0.382769481	6.234779545	4.067127424	6.05E-05	1.83E-03
RPL22L1	-0.031333376	0.383411364	9.206280357	3.634991959	3.26E-04	6.10E-03
LMAN2	8.653044706	0.383628247	11.61797906	5.699643034	2.82E-08	9.18E-06
KPNA2	1.750452174	0.383784416	11.62373961	4.131944297	4.64E-05	1.56E-03
COX5B	5.049971305	0.383904221	12.37208133	4.93438144	1.32E-06	1.39E-04
C11orf10	6.754486637	0.384219481	10.74736429	5.307770277	2.13E-07	4.01E-05
HHIPL1	-2.600846444	0.384278571	6.138921753	2.780365402	5.76E-03	4.35E-02
A1BG	-2.555124049	0.385794156	6.396466558	2.797702648	5.47E-03	4.22E-02
CCDC124	8.58700221	0.385863312	10.7352914	5.686380387	3.02E-08	9.54E-06
C14orf2	3.590096844	0.386224026	10.62774253	4.594410181	6.34E-06	4.07E-04
DLEU2	0.509774921	0.386575	6.061168669	3.792135642	1.80E-04	3.97E-03
C19orf40	2.998642478	0.387101623	6.571409253	4.450335791	1.20E-05	6.24E-04
NDUFA2	3.730761103	0.387335714	10.21201721	4.628100318	5.45E-06	3.72E-04
ATP5L	3.651119877	0.38837013	11.66045	4.609051842	5.94E-06	3.90E-04
NEU1	4.426146982	0.388837662	10.80704026	4.791638615	2.58E-06	2.17E-04
MRPL47	10.12944256	0.389283117	9.780341558	5.989992211	5.86E-09	2.46E-06
MRPS12	1.235680784	0.389554545	10.18014351	3.994111046	8.13E-05	2.26E-03
PDE6D	10.86362304	0.390421104	8.893104708	6.130324204	2.69E-09	1.25E-06
RDM1	-2.086295558	0.390607143	4.395613961	2.969939659	3.21E-03	2.93E-02
DLGAP5	-0.196560518	0.390673052	8.692190422	3.5857578	3.91E-04	6.85E-03
TIMM17B	2.181318416	0.391083117	10.43575	4.244235038	2.91E-05	1.14E-03
HIST1H1A	-1.757159638	0.391139935	1.463210227	3.085430712	2.22E-03	2.25E-02
MARCH2	6.495422126	0.391208117	9.251684578	5.252451655	2.81E-07	4.69E-05
TTK	0.422653501	0.391292208	8.929812987	3.767238029	1.98E-04	4.27E-03
TK1	-0.812635202	0.391581494	10.41727711	3.396422692	7.73E-04	1.09E-02

TLCD1	0.983205602	0.391588312	7.806128571	3.924926843	1.07E-04	2.76E-03
AURKB	-0.915751767	0.391630844	8.604854383	3.363773482	8.66E-04	1.18E-02
MND1	-0.645891561	0.391996104	6.426062987	3.448612325	6.42E-04	9.60E-03
C6orf125	6.894201864	0.39206039	9.966308117	5.33740705	1.84E-07	3.65E-05
POLR2L	4.376542457	0.392287338	11.11830666	4.78013227	2.72E-06	2.26E-04
C6orf150	-1.918998886	0.392512013	6.673721591	3.02916	2.66E-03	2.57E-02
TMSL3	1.133060541	0.392713312	14.6508625	3.966121609	9.10E-05	2.44E-03
GPR19	-2.284824625	0.392755195	5.633587987	2.898186204	4.02E-03	3.42E-02
CDKN2D	-0.596063759	0.392958117	7.851839448	3.464067099	6.08E-04	9.30E-03
HSD17B10	7.808019213	0.393550325	10.42979789	5.528018287	6.92E-08	1.70E-05
BFSP1	-2.664899285	0.393564935	4.41067013	2.755903473	6.20E-03	4.59E-02
C19orf10	5.572732612	0.393809091	10.56453961	5.051354171	7.54E-07	9.16E-05
KIFC1	1.143911703	0.394403247	9.555820455	3.969089624	8.99E-05	2.43E-03
HMGA1	2.193608173	0.394762987	13.36649773	4.247399273	2.87E-05	1.14E-03
HLA-H	-0.582777927	0.395001623	9.620254708	3.468177152	5.99E-04	9.20E-03
REEP2	-2.196110155	0.39538961	7.493095455	2.930454773	3.64E-03	3.19E-02
PPIH	6.816364874	0.39608961	9.396824675	5.320912859	1.99E-07	3.86E-05
C1orf59	-1.166208338	0.396208117	7.927824838	3.283221297	1.14E-03	1.42E-02
C12orf75	-0.685682595	0.396386039	8.997337825	3.436224539	6.71E-04	9.89E-03
DDA1	9.951249884	0.396794156	10.59159448	5.955543879	7.08E-09	2.91E-06
GEMIN8P4	1.605140363	0.396894481	5.198643994	4.093456688	5.44E-05	1.70E-03
BCAS4	1.471499173	0.397143182	8.603845617	4.057772729	6.29E-05	1.87E-03
HSH2D	-2.598863088	0.39715974	8.227768182	2.781119589	5.75E-03	4.35E-02
NDUFB11	4.606575062	0.397191558	11.38384123	4.833293195	2.12E-06	1.89E-04
CCDC109B	-1.629392934	0.397236688	8.386817695	3.129183542	1.92E-03	2.04E-02
ZNF563	2.530748498	0.398253247	5.903964286	4.33340499	1.99E-05	8.87E-04
ECHDC3	-0.51637794	0.398282792	8.666263474	3.488651472	5.56E-04	8.75E-03
MRPL11	7.035042132	0.398593831	10.33965406	5.367145826	1.58E-07	3.34E-05
C20orf24	9.235695677	0.398655844	11.17768117	5.81560014	1.52E-08	5.29E-06
HTATIP2	-0.29698597	0.399605844	8.864554221	3.555528095	4.37E-04	7.42E-03
MELK	-0.802553941	0.399807792	8.799983117	3.399599085	7.64E-04	1.08E-02
C1orf135	1.236035866	0.399840584	6.953496266	3.994207588	8.13E-05	2.26E-03
MAGIX	-0.941934134	0.400357468	7.366543669	3.355436686	8.92E-04	1.20E-02
ILVBL	5.130243902	0.401705195	10.51875519	4.952494837	1.21E-06	1.30E-04
NDUFA4	3.698981727	0.402448052	11.78161039	4.620507522	5.64E-06	3.82E-04
PFDN6	6.448382885	0.404047727	9.077198539	5.242355432	2.95E-07	4.85E-05
NDC80	0.117674351	0.404316883	8.134938961	3.678875666	2.77E-04	5.44E-03
KHDC1	-2.026231585	0.404533766	5.330191558	2.99132831	3.00E-03	2.79E-02
ISG20	-1.787948194	0.405232792	7.836225487	3.074800158	2.30E-03	2.32E-02
MAPK8IP1	-1.496754811	0.40564513	10.24547192	3.174006785	1.66E-03	1.85E-02
PLBD1	-1.674493997	0.406813961	8.800889448	3.113804774	2.02E-03	2.11E-02
FAM46D	-2.529063267	0.406909091	1.226986364	2.807539049	5.31E-03	4.14E-02
SPINT2	0.361544517	0.407531494	13.73977484	3.749684688	2.12E-04	4.45E-03
ATP6V0B	6.004810812	0.407731169	11.66894026	5.14634158	4.75E-07	6.72E-05
ADAM21P1	-1.03950154	0.408412338	1.42446461	3.324199366	9.94E-04	1.29E-02

C20orf7	8.483883239	0.40844513	7.435625812	5.665622237	3.37E-08	1.03E-05
LSM10	5.499056007	0.408922403	9.17753263	5.03500727	8.16E-07	9.68E-05
LOC647121	-1.319524976	0.408958442	5.37721039	3.232992226	1.36E-03	1.61E-02
DDX49	13.36823005	0.409119805	10.23684562	6.591612825	1.89E-10	1.34E-07
HLA-B	-2.620500331	0.409568831	14.08217597	2.772881331	5.90E-03	4.43E-02
C19orf52	11.15491794	0.410127597	8.235607305	6.185316073	1.97E-09	9.65E-07
ZNF99	-2.07936508	0.410233117	1.649789935	2.972414986	3.19E-03	2.92E-02
TMED9	13.537355	0.410627922	11.4286789	6.621883998	1.58E-10	1.16E-07
GYLTL1B	-1.174978758	0.411579545	10.06147289	3.28036727	1.16E-03	1.43E-02
GSTM3	-2.706106598	0.41165487	8.223618344	2.740056675	6.50E-03	4.74E-02
UBA52	5.931252585	0.411890909	13.86156169	5.130274789	5.14E-07	7.13E-05
DCAF15	4.743089322	0.413055844	10.32713247	4.864607002	1.84E-06	1.76E-04
PRKCSH	9.338523521	0.413062338	13.26557987	5.835873715	1.36E-08	5.13E-06
GIPC1	7.160400775	0.413940909	12.03988344	5.393502077	1.38E-07	3.02E-05
NDUFA11	3.191179205	0.414051623	10.91482516	4.497677695	9.75E-06	5.44E-04
DNAJB11	14.06343988	0.414225974	10.64447922	6.715399545	9.08E-11	7.45E-08
LAGE3	2.990080866	0.414366558	9.460835227	4.4482204	1.21E-05	6.28E-04
TAP1	-1.951515319	0.414581494	11.18432516	3.017735339	2.76E-03	2.63E-02
FAM64A	0.119588956	0.414842208	7.767743182	3.679436418	2.76E-04	5.43E-03
C6orf126	-1.654834403	0.415347727	1.031532305	3.120517119	1.98E-03	2.07E-02
IMMP1L	6.513314933	0.41554513	6.430032305	5.256287837	2.76E-07	4.69E-05
NR2F6	3.736416122	0.416477922	12.09591429	4.629450298	5.42E-06	3.71E-04
PGLYRP2	-0.819761171	0.418146104	1.101267208	3.394175789	7.79E-04	1.10E-02
SH2D2A	-0.266063469	0.418713636	6.695822403	3.564861337	4.22E-04	7.22E-03
CCNA2	1.413195576	0.418755844	9.612487662	4.042116679	6.70E-05	1.97E-03
FAM46B	-2.573110415	0.419133442	6.717594643	2.790894682	5.59E-03	4.28E-02
MCM10	-0.401898891	0.419420455	7.807065422	3.523693436	4.90E-04	7.99E-03
CLCN4	-2.117924395	0.419481169	7.454854221	2.958618161	3.33E-03	3.01E-02
COX6B1	4.641521223	0.41991039	12.37099481	4.841325636	2.05E-06	1.87E-04
PDIA6	15.74409574	0.420097727	13.02317289	7.007994974	1.54E-11	1.66E-08
USE1	5.991582185	0.420686039	9.192620292	5.143455241	4.82E-07	6.77E-05
ASB2	-1.126157952	0.420718182	5.199926623	3.296225094	1.09E-03	1.38E-02
USMG5	3.446200438	0.420862662	9.947247565	4.559722859	7.41E-06	4.58E-04
CCNB2	2.288731246	0.422543831	9.739084253	4.271820359	2.59E-05	1.06E-03
CKS2	1.20041461	0.422769805	9.906041396	3.984512201	8.45E-05	2.32E-03
TUBA1C	12.41555603	0.42327987	12.89621396	6.419116601	5.19E-10	3.13E-07
C1orf51	1.096713751	0.423472078	7.698045779	3.956165434	9.46E-05	2.52E-03
MAN2B1	7.492445511	0.423658442	11.35763442	5.462808698	9.69E-08	2.24E-05
ATP5G1	2.787808224	0.424538636	10.31665308	4.397984237	1.51E-05	7.28E-04
SDF2L1	2.797351801	0.426297078	8.677722565	4.400365761	1.49E-05	7.23E-04
C1orf14	-0.545549617	0.42738474	0.710437175	3.479670111	5.75E-04	8.94E-03
ZNF878	-1.409356735	0.428061039	2.724783766	3.203221192	1.50E-03	1.74E-02
CCNI2	-2.546428165	0.42820487	3.958425812	2.800988471	5.42E-03	4.19E-02
ZNF695	-0.405797606	0.428360065	6.283515097	3.52250532	4.92E-04	8.02E-03
NACC1	5.040381027	0.429055519	12.15532516	4.932213638	1.33E-06	1.39E-04

ZNF564	7.826878536	0.429541234	8.374484903	5.531895518	6.79E-08	1.70E-05
CHAC1	-0.676987916	0.429818182	7.573594805	3.438934893	6.65E-04	9.85E-03
KCNE2	1.808986261	0.430424675	3.94336039	4.147357753	4.36E-05	1.50E-03
PLIN2	0.172138969	0.430515909	10.06545731	3.694796994	2.60E-04	5.19E-03
GPR89B	-2.448765481	0.430662662	6.452195617	2.837643385	4.85E-03	3.87E-02
ATG4D	6.165216241	0.431006169	9.114681006	5.181233335	4.00E-07	6.04E-05
NDUFA3	3.291639297	0.43133539	10.30179821	4.5222075	8.75E-06	5.10E-04
TRAPPC5	6.437686805	0.432358442	10.30679481	5.240057437	2.99E-07	4.87E-05
KIF2C	1.531591703	0.432505195	9.545242208	4.073852796	5.89E-05	1.79E-03
MANF	14.63275605	0.433673377	10.38866591	6.815534357	4.97E-11	4.25E-08
CDCA8	1.682085498	0.435260714	9.438992695	4.113876556	5.00E-05	1.61E-03
HLA-F	-2.080086037	0.436974675	9.352890584	2.972157575	3.19E-03	2.92E-02
CDKN3	0.849219178	0.437122078	8.022665584	3.887761558	1.24E-04	3.06E-03
CNFN	-2.744886852	0.438965584	6.784707468	2.725063238	6.80E-03	4.88E-02
H2BFWT	-1.849711785	0.440237013	0.905951623	3.053369984	2.46E-03	2.43E-02
HLA-A	1.129522085	0.440432143	13.3840414	3.965153337	9.13E-05	2.45E-03
PIR	-0.562081115	0.44073539	7.306070292	3.474570906	5.85E-04	9.05E-03
TMED1	11.25031141	0.441669805	9.180564123	6.203243044	1.78E-09	8.99E-07
DSCR9	0.448807161	0.442038961	3.210622727	3.774727902	1.92E-04	4.17E-03
C7orf13	-0.017654131	0.442347078	8.073299513	3.63904074	3.21E-04	6.04E-03
HJURP	2.944527256	0.442597727	8.9950625	4.436950239	1.27E-05	6.47E-04
ITPKA	-2.332920736	0.443726299	3.912785877	2.880549256	4.25E-03	3.55E-02
C19orf62	11.24514144	0.445446429	11.00722451	6.202272493	1.79E-09	8.99E-07
MRPL4	6.677964171	0.445681169	11.2645724	5.291480073	2.31E-07	4.20E-05
EIF5A2	-0.218125624	0.446355519	6.891606331	3.579286102	4.00E-04	6.97E-03
TECR	6.979009946	0.44737013	11.61775	5.355330774	1.68E-07	3.45E-05
ECSIT	5.817425893	0.44753961	9.848886039	5.10532912	5.80E-07	7.80E-05
MX2	-1.408820312	0.448357143	10.22828052	3.203399734	1.50E-03	1.74E-02
UQCRQ	4.041027737	0.448836688	11.00360666	4.701672543	3.90E-06	2.93E-04
ZNF492	-1.440014087	0.449249675	5.175459253	3.193001831	1.55E-03	1.77E-02
KIAA0101	1.466383255	0.449375	8.714153084	4.056401135	6.32E-05	1.88E-03
FDX1L	7.685319945	0.449488312	8.390725974	5.502738716	7.89E-08	1.91E-05
AGMAT	-0.246434881	0.449856169	5.887356006	3.570774152	4.13E-04	7.11E-03
CRB3	1.170946693	0.450606818	8.619295617	3.976475584	8.73E-05	2.38E-03
FAM54A	2.284726877	0.450668506	6.546088799	4.270794802	2.60E-05	1.07E-03
C18orf56	-1.579182943	0.451790584	4.674746591	3.146221572	1.82E-03	1.96E-02
PIN1	11.43877031	0.45225974	10.09198961	6.238542955	1.46E-09	7.69E-07
ZNF440	1.999930211	0.454286039	8.53657289	4.197287547	3.54E-05	1.29E-03
B3GNT4	-1.908719072	0.454512013	4.125957305	3.032763397	2.63E-03	2.55E-02
LOC643719	-2.072554838	0.454663312	4.375205032	2.974845477	3.16E-03	2.90E-02
SSR4	3.320717676	0.457038636	11.67015698	4.529286098	8.48E-06	4.99E-04
RAX	-2.673095752	0.458095455	1.132928247	2.752758325	6.26E-03	4.62E-02
PACSIN1	-1.94189807	0.45918539	7.752964123	3.021118594	2.73E-03	2.61E-02
ATP5J2	6.012862783	0.459582143	11.94466899	5.148097768	4.71E-07	6.71E-05
MRPS15	7.363240893	0.46	10.67885974	5.435926214	1.11E-07	2.51E-05

PDCD5	11.81042361	0.460376299	9.819248539	6.307712259	9.86E-10	5.62E-07
ELOF1	15.89525314	0.462327922	10.17263019	7.033879262	1.31E-11	1.49E-08
CCNB1	5.100783014	0.463906818	10.3011164	4.945853559	1.25E-06	1.34E-04
GNGT1	-2.655769545	0.46569513	2.883931331	2.759402727	6.14E-03	4.56E-02
LOC728875	-1.108386776	0.46915974	6.200829221	3.301979896	1.07E-03	1.36E-02
CD320	5.823668746	0.469319156	10.01320763	5.106699916	5.76E-07	7.80E-05
HNF4A	-1.507804187	0.47231461	0.754107955	3.170295429	1.68E-03	1.86E-02
C1orf161	-1.519355071	0.472406818	1.169845617	3.166411277	1.70E-03	1.87E-02
UBE2S	4.208856824	0.472993506	9.521307143	4.741058575	3.26E-06	2.58E-04
ABHD8	8.022443977	0.473717208	9.608085877	5.571972041	5.51E-08	1.57E-05
DMRTA2	-2.428509462	0.473927922	1.47426461	2.845189862	4.74E-03	3.81E-02
PDIA4	15.00308789	0.47539513	12.87190731	6.88009915	3.36E-11	3.14E-08
RFXANK	9.870705837	0.47564026	10.85744156	5.939921624	7.72E-09	3.05E-06
MESP1	-2.175032919	0.477024026	6.397366558	2.938072101	3.55E-03	3.14E-02
IFI30	0.576018076	0.477628571	11.5062	3.810967803	1.67E-04	3.80E-03
ART5	-1.614697242	0.478100325	4.050844318	3.134179283	1.89E-03	2.01E-02
APOBEC3A	-2.514443642	0.478481494	4.296312825	2.813042808	5.22E-03	4.08E-02
LYPD3	-2.660680158	0.479551948	6.93252987	2.757521108	6.17E-03	4.57E-02
LSM4	12.3705547	0.48096526	11.65633198	6.41088242	5.45E-10	3.19E-07
DHPS	12.74971973	0.481131169	10.08478766	6.480013628	3.64E-10	2.27E-07
BCL2L14	-1.91702936	0.481944156	5.282285065	3.029850692	2.65E-03	2.57E-02
FAM83D	3.995914712	0.483042532	9.459851136	4.69103695	4.10E-06	3.04E-04
MGC72080	3.7914594	0.483921753	7.94944789	4.642572625	5.11E-06	3.53E-04
ROMO1	7.999203327	0.483938636	10.47331607	5.567221668	5.65E-08	1.57E-05
PPIC	2.691031109	0.484099675	8.924509578	4.37377001	1.67E-05	7.92E-04
TSPAN13	1.447904952	0.484244805	9.28664513	4.051443583	6.45E-05	1.91E-03
HAUS8	9.232486854	0.485503247	7.850898377	5.814966585	1.52E-08	5.29E-06
RGS9BP	-1.782777646	0.485643831	3.552232955	3.076587832	2.28E-03	2.31E-02
SERPINI1	-0.663347441	0.48801461	6.242948214	3.443183008	6.55E-04	9.73E-03
TUBB8	-1.736298962	0.488157792	2.752178896	3.092613924	2.17E-03	2.21E-02
UBE2T	5.810047231	0.488573377	9.040693831	5.103708527	5.85E-07	7.80E-05
CABYR	0.840257298	0.489186039	6.710911851	3.88526426	1.25E-04	3.08E-03
PRR16	-2.370368885	0.489362338	4.466524675	2.866745916	4.43E-03	3.64E-02
CSRP2	-0.400751701	0.489741558	7.957701299	3.524042967	4.90E-04	7.99E-03
C19orf39	11.68481592	0.491062338	6.714214935	6.284400215	1.13E-09	6.25E-07
HLA-C	0.554630469	0.492685714	13.88931883	3.804896796	1.71E-04	3.85E-03
SKA1	2.931728796	0.495170455	7.364597565	4.433779334	1.29E-05	6.54E-04
FABP3	-1.839329242	0.495647403	7.519108117	3.056982286	2.43E-03	2.42E-02
PPP1R14A	-2.174804681	0.497422078	7.816816883	2.938154484	3.55E-03	3.14E-02
APOL4	-2.508711979	0.497966883	7.925493182	2.815197787	5.19E-03	4.07E-02
CSTA	-1.747251465	0.498387013	5.768655844	3.088844468	2.19E-03	2.24E-02
NDUFS5	9.294327159	0.500647403	11.24444383	5.827166805	1.42E-08	5.13E-06
MPV17L2	15.01379592	0.502402597	8.687235065	6.88195949	3.32E-11	3.14E-08
SELM	-2.155229498	0.502984091	9.58327776	2.945212131	3.47E-03	3.09E-02
TMEM37	0.38365189	0.503908766	6.879339448	3.756043563	2.06E-04	4.40E-03

POP4	5.307093142	0.50405	9.882191234	4.992204133	1.00E-06	1.15E-04
LASS4	1.683814373	0.509106169	9.379099838	4.11433433	4.99E-05	1.61E-03
COPE	22.41665312	0.509307468	12.17471477	8.094987249	1.35E-14	3.47E-11
PTTG1	3.907054998	0.51010974	9.63688539	4.670026865	4.51E-06	3.24E-04
AKR1B1	5.811060173	0.511471104	10.42732841	5.103931027	5.84E-07	7.80E-05
NHLRC1	2.41612901	0.51383539	7.394368344	4.304335394	2.25E-05	9.47E-04
HES6	0.374929724	0.51533474	7.878930357	3.753535924	2.08E-04	4.42E-03
FBXW9	8.041857373	0.516253247	8.850909091	5.575937613	5.40E-08	1.57E-05
LRRC31	-1.33207895	0.517308766	0.753762825	3.228847115	1.38E-03	1.63E-02
LOC113230	-0.90044019	0.517893831	8.366606006	3.368640021	8.52E-04	1.17E-02
YIPF2	17.41870963	0.521385714	11.04534416	7.291106173	2.62E-12	3.85E-09
CENPA	3.383272532	0.522767857	8.056237175	4.544481393	7.93E-06	4.77E-04
RAD23A	21.79267519	0.523311688	12.12474481	7.997551784	2.61E-14	5.95E-11
PSMB9	-1.130652761	0.523835714	9.714023052	3.29476807	1.10E-03	1.38E-02
DEPDC1B	3.058326738	0.523866558	7.675023539	4.465058145	1.13E-05	6.03E-04
ASCL2	-1.782520047	0.52388539	6.410484903	3.07667687	2.28E-03	2.31E-02
BGLAP	6.270308344	0.527061688	6.188759416	5.203987359	3.57E-07	5.60E-05
BATF2	-0.093404467	0.52896461	7.372465422	3.616568083	3.49E-04	6.34E-03
TMEM205	9.905279645	0.529209091	10.99255714	5.946631482	7.44E-09	2.99E-06
TNFSF11	-2.600922403	0.529944156	2.325035065	2.780336514	5.77E-03	4.35E-02
STX10	12.97768232	0.530494481	8.484569318	6.521311386	2.86E-10	1.96E-07
ZNF98	-2.354320619	0.530542208	4.489752273	2.872668968	4.35E-03	3.61E-02
TCL1B	-0.509866741	0.530917857	0.995114773	3.49065323	5.52E-04	8.72E-03
BIRC5	3.657415707	0.53119026	9.304375649	4.610560149	5.90E-06	3.89E-04
NDUFA13	12.92088697	0.531728247	11.72022516	6.511040681	3.04E-10	2.00E-07
TMEM38A	3.558340741	0.531894481	7.526506981	4.586774707	6.56E-06	4.17E-04
FZD9	0.276085882	0.532561364	4.146103409	3.725010887	2.32E-04	4.77E-03
C6orf129	9.327449136	0.533264935	9.078653247	5.833692973	1.37E-08	5.13E-06
CDKN2BAS	-2.648320902	0.534557143	4.088437662	2.762254529	6.09E-03	4.53E-02
WFDC2	-0.679790494	0.536044805	14.17204383	3.438061473	6.67E-04	9.86E-03
NDUFA7	8.990516721	0.53667013	10.10084286	5.767032272	1.97E-08	6.73E-06
ACPP	-2.703968602	0.537234091	7.428315097	2.74088101	6.49E-03	4.73E-02
RPL39L	5.144485134	0.537947403	9.016589286	4.955702491	1.19E-06	1.29E-04
UQCRFS1	10.65040252	0.538306169	11.62200308	6.089828425	3.37E-09	1.54E-06
TTC39A	-1.079710645	0.539215584	8.154627273	3.311246428	1.04E-03	1.33E-02
IGFBP2	1.646984892	0.541741558	13.82191234	4.104572726	5.20E-05	1.65E-03
KLHL35	0.038736228	0.544227273	7.074561688	3.655687849	3.02E-04	5.74E-03
DEFB4A	-1.099609372	0.544888636	1.000200812	3.304818829	1.06E-03	1.35E-02
ACP5	2.249362265	0.547169805	9.436619318	4.261728117	2.70E-05	1.09E-03
SECTM1	-2.186130562	0.548946753	9.130627922	2.934063731	3.60E-03	3.17E-02
NANOS3	3.162964961	0.551824026	3.565931494	4.490767491	1.01E-05	5.55E-04
MAST1	-0.119388251	0.55325487	7.274871591	3.608830046	3.59E-04	6.46E-03
CXorf59	-1.973369321	0.554032468	1.826971429	3.010034017	2.83E-03	2.67E-02
ABCC6P2	-0.093797898	0.554051623	3.842708929	3.616451031	3.49E-04	6.34E-03
CR1L	-1.44889212	0.557353896	2.265945779	3.19003671	1.57E-03	1.78E-02

FARSA	23.31574663	0.559760065	10.85915795	8.234091148	5.25E-15	1.54E-11
MRPL34	16.08890132	0.561095779	10.31525244	7.066940964	1.07E-11	1.29E-08
HOXC5	-2.018244051	0.561134416	6.266778247	2.994161812	2.98E-03	2.77E-02
IFI6	-1.944491885	0.562704221	13.31088847	3.020206466	2.74E-03	2.62E-02
SLPI	-2.249687075	0.564083117	13.37911299	2.911007366	3.87E-03	3.33E-02
KLF1	6.989735719	0.565362013	2.43902711	5.35759409	1.66E-07	3.45E-05
CENPW	0.389976047	0.57014513	8.176019968	3.757860817	2.05E-04	4.38E-03
CDC25C	6.063346313	0.57096526	7.371718994	5.159097177	4.46E-07	6.49E-05
BHLHA15	1.350415118	0.57279513	4.241024838	4.025197526	7.18E-05	2.07E-03
PLEKHF1	2.201532922	0.572817857	9.088114773	4.249438546	2.85E-05	1.13E-03
GDF5	-1.907422229	0.575735065	4.198956494	3.033217696	2.63E-03	2.55E-02
WDR83	16.77554942	0.576896104	7.569413636	7.183301871	5.17E-12	6.64E-09
CDC20	6.723380308	0.577508442	10.51501136	5.301153311	2.20E-07	4.11E-05
MICB	0.048061707	0.581240584	7.381855357	3.65843417	2.99E-04	5.72E-03
FOLR1	-1.94901322	0.58194513	11.84553231	3.018615896	2.75E-03	2.63E-02
ISG15	-0.991621197	0.588827922	11.11131721	3.339562671	9.43E-04	1.25E-02
DAND5	-1.395405048	0.590771753	2.732398214	3.207861805	1.48E-03	1.72E-02
CLCN1	1.744606725	0.592026948	1.552811526	4.130402233	4.67E-05	1.56E-03
CRIP3	-0.984420656	0.592358442	3.307746104	3.34186743	9.35E-04	1.24E-02
SYT13	-2.58529962	0.592361688	7.83704513	2.786272011	5.66E-03	4.31E-02
UBE2C	6.679045899	0.592644156	10.62352792	5.291710641	2.31E-07	4.20E-05
GDF3	-1.78928671	0.592938961	2.230908442	3.074337218	2.30E-03	2.32E-02
UBB	-0.953092498	0.593315909	12.83839302	3.351877883	9.03E-04	1.21E-02
ACOT4	5.723069055	0.594258442	6.608188312	5.084572679	6.42E-07	8.24E-05
C12orf56	-1.132833448	0.595650974	5.319330682	3.294060969	1.10E-03	1.39E-02
BST2	-0.141740327	0.595842532	12.55009399	3.602161362	3.68E-04	6.60E-03
MYBL2	2.952244336	0.596323052	10.73155633	4.43886124	1.26E-05	6.45E-04
C19orf43	27.71638807	0.596979545	11.96962419	8.895359792	5.11E-17	2.10E-13
C19orf56	21.21948026	0.597462987	11.37064058	7.907370252	4.77E-14	9.80E-11
XG	-2.407912866	0.598321104	4.323027435	2.852843838	4.63E-03	3.75E-02
LDHC	-2.703778139	0.60065974	3.398741558	2.740954434	6.48E-03	4.73E-02
GCDH	18.74282345	0.604848052	9.150570779	7.509657612	6.49E-13	1.03E-09
AKR1B15	-1.505530727	0.60554026	1.339538961	3.17105939	1.67E-03	1.85E-02
ALPL	-1.287396751	0.610023377	10.67611429	3.243577937	1.31E-03	1.57E-02
GZMB	-2.746570924	0.612724675	5.128292208	2.724410355	6.81E-03	4.89E-02
LOC284749	-2.529448269	0.613765584	3.584192532	2.807393971	5.31E-03	4.14E-02
TRY6	-2.002291268	0.615069481	2.382570455	2.99981337	2.92E-03	2.74E-02
NPTX2	-2.229852765	0.616026299	7.831000812	2.918221109	3.78E-03	3.27E-02
NMU	-1.236886766	0.622815584	7.316382468	3.260155379	1.24E-03	1.50E-02
GGH	6.661680596	0.623943506	9.225953571	5.288008252	2.35E-07	4.24E-05
UBL5	16.7917247	0.62564513	10.9900914	7.186026948	5.08E-12	6.64E-09
C15orf48	-0.650226002	0.626797078	7.19236737	3.447264917	6.45E-04	9.64E-03
CSAG2	-2.263235055	0.628661039	1.721898701	2.906070243	3.93E-03	3.36E-02
C19orf53	19.64616254	0.63067013	11.52668766	7.656308084	2.50E-13	4.28E-10
CCL8	-1.078437442	0.631200649	6.094580844	3.311657298	1.04E-03	1.33E-02

C19orf57	6.019621454	0.631237662	7.130120779	5.149571493	4.67E-07	6.71E-05
CCL7	-1.695962755	0.63399026	3.342499026	3.106459213	2.07E-03	2.14E-02
NAP1L6	-2.667275286	0.636168831	2.064347403	2.754992105	6.22E-03	4.60E-02
HSPC072	-0.241136757	0.636839935	2.421916071	3.572368593	4.11E-04	7.07E-03
LAMP3	0.0540478	0.638553896	8.704141883	3.660196061	2.97E-04	5.69E-03
DDX39	15.69507911	0.640182468	10.79575097	6.99958668	1.62E-11	1.66E-08
NDUFB7	13.58689128	0.64250487	11.38486542	6.63073094	1.50E-10	1.14E-07
HLA-G	-2.309468191	0.645769805	4.189704383	2.889162069	4.14E-03	3.49E-02
DEFB126	-1.013813726	0.645921429	1.597021104	3.332449953	9.66E-04	1.27E-02
NRG4	-2.016319651	0.647414286	4.336426623	2.994844097	2.97E-03	2.77E-02
CXCL10	-2.308827813	0.649369805	9.578942695	2.889396904	4.13E-03	3.49E-02
MGC87042	1.673450176	0.654018831	3.69398474	4.111589404	5.05E-05	1.61E-03
DNASE2	37.74349466	0.654828896	10.80995016	10.30962714	1.34E-21	1.38E-17
IFI27	-1.076653684	0.655732468	12.66453831	3.312232848	1.04E-03	1.33E-02
HOXA6	-1.757707007	0.656737662	2.078379221	3.085242018	2.22E-03	2.25E-02
ASNA1	30.72239016	0.656747078	11.36537679	9.33108223	2.16E-18	1.48E-14
C1orf182	4.637667396	0.667563312	3.988983604	4.840440385	2.06E-06	1.87E-04
ASF1B	10.54767092	0.668006494	10.04400844	6.070242409	3.76E-09	1.68E-06
MAT1A	-1.519945781	0.670322727	6.122627597	3.166212523	1.70E-03	1.87E-02
ADAM21	1.200335043	0.673217857	3.243056981	3.984490521	8.45E-05	2.32E-03
IGFBPL1	-1.576152186	0.676637013	3.049662013	3.147247253	1.81E-03	1.95E-02
GADD45GIP1	25.6310532	0.679421104	10.47543133	8.585806975	4.59E-16	1.57E-12
ECE2	14.72555073	0.681200974	8.997945942	6.831754016	4.51E-11	4.02E-08
DSCR6	-1.038898527	0.682728896	4.102309253	3.324393264	9.93E-04	1.29E-02
LOC389458	-0.173451127	0.683774675	2.510754221	3.592681073	3.81E-04	6.73E-03
SLC22A16	-0.310804571	0.687222727	2.154188636	3.551349989	4.43E-04	7.49E-03
MESP2	2.095059743	0.687682143	4.468517045	4.221967001	3.19E-05	1.22E-03
LOC440356	-1.286268263	0.690272078	3.009306818	3.243949169	1.31E-03	1.56E-02
GATA2	-0.861285607	0.690694156	6.004501623	3.381055162	8.15E-04	1.13E-02
PRDX2	21.0560502	0.692630519	12.56800162	7.881535552	5.67E-14	1.06E-10
GIPC2	-1.367523063	0.700969805	3.852358929	3.217117202	1.43E-03	1.68E-02
CNTD2	-0.999743386	0.70116039	4.819043831	3.336961136	9.51E-04	1.26E-02
RTBDN	-0.681793149	0.701354221	2.072351136	3.437437223	6.68E-04	9.88E-03
LYG2	-0.11614529	0.704326299	2.717191071	3.609796638	3.58E-04	6.45E-03
INA	-2.654412196	0.706678896	5.926906981	2.759922613	6.13E-03	4.56E-02
LRRIQ4	5.676847054	0.714155519	1.999553084	5.074378866	6.74E-07	8.39E-05
CCNE1	3.524419994	0.717277273	9.355839935	4.578606568	6.81E-06	4.30E-04
CACNA1A	4.374186547	0.718651299	7.771184091	4.779585199	2.73E-06	2.26E-04
FOLR3	-2.714461522	0.719544156	4.789448052	2.736833049	6.56E-03	4.77E-02
ZDHHC19	0.174196456	0.727983766	2.790350974	3.69539722	2.60E-04	5.19E-03
GAL	-1.962116942	0.734252597	4.514037987	3.014001653	2.79E-03	2.65E-02
SLC30A2	-2.232478015	0.740151948	5.529958442	2.917267272	3.79E-03	3.28E-02
KAZALD1	0.57544276	0.742421104	6.926717695	3.81080461	1.67E-04	3.80E-03
SPC24	12.8970014	0.748647727	6.766198539	6.506717661	3.12E-10	2.00E-07
HOXA9	-2.707886191	0.748681494	3.539316071	2.739370347	6.52E-03	4.75E-02

KCNK9	-1.981974143	0.749234091	4.458435227	3.006996599	2.86E-03	2.69E-02
GPR160	4.495237176	0.74929026	8.540239286	4.807625593	2.39E-06	2.07E-04
LOC389493	-1.412044678	0.750080844	5.904368344	3.202326407	1.51E-03	1.74E-02
PRSS3	1.423847756	0.751286039	3.338601461	4.044981119	6.62E-05	1.95E-03
APOA1	-2.328614651	0.755342532	9.706210227	2.882132465	4.23E-03	3.54E-02
KBTBD12	-2.032992589	0.759767532	5.025886364	2.988927931	3.03E-03	2.80E-02
GSC	-1.606438401	0.760629221	3.839603571	3.136983574	1.87E-03	2.00E-02
USH1C	-1.520652382	0.762914935	2.425587987	3.16597476	1.70E-03	1.87E-02
HOXB13	-2.768613667	0.774524351	1.69616737	2.715851033	6.98E-03	4.98E-02
CXCL5	-2.02963001	0.780817532	4.730942532	2.990121984	3.01E-03	2.79E-02
CHRDL2	2.441305177	0.783311039	2.103374351	4.31073534	2.19E-05	9.33E-04
ORM1	-1.222353341	0.783855844	1.681385065	3.264910747	1.22E-03	1.49E-02
HOXA7	-1.885117017	0.797509416	4.952080682	3.041021474	2.56E-03	2.50E-02
GDF15	4.099710167	0.797932792	7.654304708	4.715476266	3.66E-06	2.79E-04
RNASEH2A	27.74844854	0.798781494	9.676129708	8.900069831	4.94E-17	2.10E-13
KRT13	-2.327370154	0.804172403	3.132964773	2.882589873	4.22E-03	3.54E-02
CALR	129.7043158	0.805101623	14.6246112	21.74628034	4.00E-64	8.21E-60
SYCE2	11.49396613	0.806792532	5.497109903	6.248852599	1.38E-09	7.45E-07
GFRA3	0.733626788	0.816652597	4.250421753	3.855438747	1.41E-04	3.34E-03
ELOVL3	2.510950145	0.824350649	4.679903896	4.328396062	2.03E-05	8.95E-04
HOXA10	-2.294774846	0.849777597	4.472009578	2.894545771	4.07E-03	3.45E-02
IGF2BP3	-2.138563501	0.853415909	7.332346916	2.951208351	3.41E-03	3.06E-02
HTR3A	-2.561147457	0.880128896	7.752794968	2.795424497	5.51E-03	4.24E-02
CDKN2A	-0.185669758	0.889781818	10.71338701	3.589022056	3.86E-04	6.79E-03
ALG1L	1.715511502	0.899904221	4.856031981	4.1227191	4.82E-05	1.59E-03
PCDHB5	-0.547328311	0.900390584	7.129642045	3.479121797	5.76E-04	8.95E-03
XAGE2	-2.439821957	0.905222727	3.059097727	2.840977679	4.80E-03	3.85E-02
SLC15A1	2.695944346	0.928787013	4.097357143	4.375002167	1.67E-05	7.90E-04
FXYD4	0.325815594	0.944040909	2.536476299	3.739386979	2.20E-04	4.59E-03
LGALS7	3.937963385	0.971962662	3.291486526	4.677344106	4.36E-06	3.20E-04
MS4A15	1.098389491	0.97633961	4.248193182	3.956624951	9.45E-05	2.52E-03
C3orf55	1.751696434	1.010922403	4.048933279	4.132272473	4.64E-05	1.56E-03
PRSS1	2.168704444	1.012262987	6.143309416	4.240985155	2.95E-05	1.15E-03
GTSF1	-1.710933826	1.012951623	3.666719968	3.101327196	2.11E-03	2.17E-02
CT45A5	-2.599331029	1.013278571	2.572936039	2.780941668	5.75E-03	4.35E-02
PHOX2A	-1.853566556	1.022118831	6.162759416	3.052027807	2.47E-03	2.44E-02
PDCL2	2.267098314	1.03699513	1.832971591	4.266277368	2.65E-05	1.08E-03
PPP1R1C	2.461154336	1.03875974	3.438998701	4.315775224	2.15E-05	9.20E-04
MAGEA11	-2.568694503	1.070240584	2.813913799	2.792567594	5.56E-03	4.27E-02
PLAC1	1.857486479	1.072007468	2.995390747	4.160090515	4.13E-05	1.45E-03
RHCG	3.427571468	1.083291883	3.695945942	4.555215444	7.56E-06	4.63E-04
SST	-2.600287426	1.095100325	8.131533279	2.780577992	5.76E-03	4.35E-02
CT45A2	-1.352868581	1.10051461	2.441436526	3.2219718	1.41E-03	1.66E-02
C10orf99	0.767049465	1.109505844	3.629890584	3.864809754	1.36E-04	3.25E-03
AGR2	0.649180187	1.124702597	5.924978571	3.831669382	1.54E-04	3.59E-03

MMP1	1.860875708	1.132491883	5.185156331	4.160978994	4.12E-05	1.45E-03
ABP1	-0.411945721	1.187475	7.294890747	3.520630958	4.96E-04	8.06E-03
LGALS7B	3.274492335	1.217231818	3.354543831	4.518028861	8.91E-06	5.14E-04
CLDN22	0.071735286	1.27420974	1.664272403	3.66539751	2.91E-04	5.63E-03
FABP6	1.079438217	1.343200974	4.322792695	3.951425379	9.64E-05	2.56E-03
TKTL1	-0.143720962	1.504493506	3.552188312	3.601569899	3.69E-04	6.61E-03

Supplemental Table 7. **List of genes in boxplot significantly overrepresented in *CALR*^{Hi} versus *CALR*^{Lo} HGSC samples from TCGA public database**

Connection	Gene symbol	log2(FC)	P-value
Activated DC	IDO1	1.08649026	1.67E-03
Activated DC	LAMP3	1.100985714	2.50E-05
Antigen processing	CD8A	0.523277922	4.78E-02
Antigen processing	TAPBP	0.294956494	2.38E-02
Antigen processing	TAP2	0.375432468	3.56E-02
Antigen processing	MR1	0.455369481	1.44E-03
Antigen processing	HLA-A	0.801637013	3.26E-06
Antigen processing	HLA-B	0.818880519	1.91E-04
Antigen processing	TAP1	0.85977987	3.15E-05
Antigen processing	HLA-C	0.866167532	1.08E-05
Antigen processing	PSMB9	1.069485714	3.60E-06
B cells function	CD38	0.942938312	2.72E-04
B cells function	CD79B	0.490076623	2.45E-02
B cells function	ADA	0.505817532	9.26E-05
B cells function	BLK	0.741601948	7.19E-03
Complement	C2	0.486945455	3.50E-02
Complement	C1R	0.521176623	1.14E-02
Complement	C3	0.647036364	4.30E-02
Cytotoxicity	GZMM	0.468430519	4.78E-02
Cytotoxicity	CD8A	0.523277922	4.78E-02
Cytotoxicity	KLRF1	0.591595455	3.48E-02
Cytotoxicity	GZMH	0.657107792	1.87E-02
Cytotoxicity	KLRD1	0.690942857	3.63E-03
Cytotoxicity	GZMA	0.691885714	2.46E-02
Cytotoxicity	NKG7	0.773351948	9.70E-03
Cytotoxicity	CD8B	0.979196104	8.05E-04
Cytotoxicity	GNLY	1.047596753	3.19E-03
Cytotoxicity	GZMB	1.148668831	6.11E-04
ER stress	ATF6	0.157575325	1.90E-02
ER stress	XBP1	0.319206494	9.26E-03
ER stress	DDIT3	0.375333766	7.79E-03
ER stress	HSP90B1	0.42846039	4.10E-08
ER stress	HSPA5	0.482238312	5.60E-08
Chemokines T-cell function	IRF1	0.360668831	4.19E-02
Chemokines T-cell function	CXCL9	0.846008442	3.48E-02
Chemokines T-cell function	CXCL11	1.107820779	1.01E-03
NK cytotoxicity	KLRF1	0.591595455	3.48E-02
NK cytotoxicity	KLRD1	0.690942857	3.63E-03
NK cytotoxicity	GZMB	1.148668831	6.11E-04

NK cytotoxicity	IFNB1	0.330520779	5.00E-02
NK cytotoxicity	KLRG1	0.391533766	1.92E-02
NK cytotoxicity	IL15RA	0.583824675	7.93E-03
T cell activation	CD40	0.575080519	1.29E-03
T cell activation	CCL5	0.593720779	3.61E-02
T phenotype	PDCD1	0.523820779	4.73E-02
T phenotype	BATF	0.546657143	1.91E-02
T phenotype	CXCL13	0.960177273	1.54E-02
T_H1 phenotype	LTA	0.476931818	2.42E-02
T_H1 phenotype	IL12RB2	0.658057143	3.51E-03
T_H1 phenotype	IFNG	0.682583766	2.54E-03
T_H1 phenotype	CD38	0.942938312	2.72E-04
